# Individual and combined effects of the *GSTM1*, *GSTT1*, and *GSTP1* polymorphisms on leukemia risk: An updated meta-analysis

**DOI:** 10.3389/fgene.2022.976673

**Published:** 2022-10-31

**Authors:** Yan Zhao, Di Wang, Cheng-Yu Zhang, Yan-Ju Liu, Xiao-Hui Wang, Meng-Ying Shi, Wei Wang, Xu-Liang Shen, Xiao-Feng He

**Affiliations:** ^1^ Heping Hospital Affiliated to Changzhi Medical College, Changzhi, Shanxi, China; ^2^ Beijing Zhendong Guangming Pharmaceutical Research Institute, Beijing, China; ^3^ Department of Hematology, Heping Hospital Affiliated to Changzhi Medical College, Changzhi, Shanxi, China; ^4^ Institute of Evidence-Based Medicine, Heping Hospital Affiliated to Changzhi Medical College, Changzhi, Shanxi, China; ^5^ Department of Epidemiology, School of Public Health to Southern Medical University, Guangzhou, China

**Keywords:** glutathione S-transferases, *GSTM1*, *GSTT1*, *GSTP1*, leukemia

## Abstract

**Background:** Several meta-analyses have analyzed the association of GSTM1 present/null, GSTT1 present/null, and GSTP1 IIe105Val polymorphisms with leukemia risk. However, the results of these meta-analyses have been conflicting. Moreover, they did not evaluate the combined effects of the three aforementioned gene polymorphisms. Furthermore, they did not appraise the credibility of the positive results. Finally, many new studies have been published. Therefore, an updated meta-analysis was conducted.

**Objectives:** To further explore the relationship of the three aforementioned gene polymorphisms with leukemia risk.

**Methods:** The crude odds ratios (ORs) and 95% confidence intervals (CIs) were applied to evaluate the association of the individual and combined effects of the three aforementioned genes. Moreover, the false-positive report probability (FPRP) and Bayesian false discovery probability (BFDP) were applied to verify the credibility of these statistically significant associations.

**Results:** Overall, the individual GSTM1, GSTT1, and GSTP1 IIe105Val polymorphisms added leukemia risk. On combining GSTM1 and GSTT1, GSTM1 and GSTP1, and GSTT1 and GSTP1 polymorphisms, positive results were also observed. However, no significant association was observed between the combined effects of these three polymorphisms with leukemia risk in the overall analysis. Moreover, when only selecting Hardy–Weinberg equilibrium (HWE) and medium- and high-quality studies, we came to similar results. However, when the FPRP and BFDP values were applied to evaluate the credibility of positive results, the significant association was only observed for the GSTT1 null genotype with leukemia risk in Asians (BFDP = 0.367, FPRP = 0.009).

**Conclusion:** This study strongly suggests a significant increase in the risk of leukemia in Asians for the GSTT1 null genotype.

## Introduction

Leukemia is a cancer of hematology, characterized by abnormal hematopoietic function and malignant cloning of white blood cells. Leukemia includes acute myeloid leukemia (AML), acute lymphoblastic leukemia (ALL), chronic myeloid leukemia (CML), and chronic lymphoblastic leukemia (CLL) (Ouerhani et al., 2011). Over the past few decades, we have made giant progress in the early diagnosis of diseases and treatment, yet the number of new cases of leukemia are still increasing, and the death cases also continue to increase. Therefore, leukemia has become one huge threat to human health (Ferlay et al., 2015). As we all know, leukemia is deemed to be a complex disease, which is determined by hereditary and environmental factors (Arruda et al., 2001; Krajinovic et al., 2001). Although previous studies showed that chemicals, ionizing radiation, and viral infections were the potential pathogenic factors of leukemia (Maia Rda and Wünsch Filho, 2013; Schüz and Erdmann, 2016), there were great individual differences in disease susceptibility when these patients were exposed to the aforementioned carcinogenic agents. Therefore, research studies on hereditary factors that affect leukemia may improve our further understanding of the pathogenesis of leukemia; in addition, they might provide new evidence for the treatment of leukemia.

Glutathione S-transferase (GST) is a kind of phase II enzyme which includes M1, P1, and T1; the main functions of the three aforementioned genes were the metabolism of xenobiotics, reactive oxygen species, and carcinogens for detoxification and metabolism (Strange et al., 2001). A partial gene deletion of *GSTM1* and *GSTT1* (null genotypes) can result in the complete absence of *GSTM1* and *GSTT1* enzyme activities; the former is located on chromosome 1 (1p13.3) and the latter is situated at chromosome 22 (22q11.2) (Pearson et al., 1993; Webb et al., 1996; Strange and Fryer, 1999). *GSTP1* gene polymorphism is a single-nucleotide polymorphism, whose polymorphism lies in exon 5 codon 105, when substitution of A with G leads to change in isoleucine (IIe) to valine (Val), thereby giving rise to decreased enzymatic activity (Harries et al., 1997; Ryberg et al., 1997). Previous research studies have indicated that the complete deletion of *GSTM1*, *GSTT1,* or *GSTP1* polymorphisms can bring about diminished gene expression and enzymatic activity (Strange et al., 1998; Strange et al., 2001; Hollman et al., 2016). The *GSTM1* and *GSTT1* showed a high degree of polymorphism, one of the polymorphisms being the entire deletion of the gene that results in the lapse of enzymatic activity (Alves et al., 2002).

Several meta-analyses analyzed the association of *GSTM1* present/null, *GSTT1* present/null, and *GSTP1* IIe105Val polymorphisms with leukemia risk. However, results of these meta-analyses were conflicting. Moreover, they did not evaluate the combined effects of the three aforementioned gene polymorphisms. Furthermore, they did not appraise the credibility of the positive results. Finally, many new studies have been published. Therefore, an updated meta-analysis was conducted.

## Materials and methods

### Search strategy

Five databases including PubMed, Embase, Web of Science, CNKI, and WanFang were applied to search the literature (deadline, 26 May 2022). The following retrieval strategy was employed: (glutathione S-transferase M1 OR GSTM1 OR glutathione S-transferase T1 OR GSTT1 OR glutathione S-transferase P1 OR GSTP1) AND (polymorphism OR genotype OR mutation OR variant OR allele) AND (leukemia OR leukaemia). Furthermore, if necessary, we contacted the corresponding authors by e-mail.

### Inclusion and exclusion criteria

The studies that met the following criteria were included: 1) case-control or cohort study, 2) genotype data or odds ratio (OR) with 95% confidence interval (CI) provided, and 3) investigation of the association of the three aforementioned gene polymorphisms with the risk of leukemia. Studies such as overlapping data, case reports, editorials, reviews, letters, and meta-analyses were excluded.

### Data extraction and quality assessment

Information was extracted and checked by two researchers from all selected studies. Any disagreement was solved through discussion. Extracted information in shown in Supplementary Tables S1–S3. Quality assessment was conducted by two authors independently (Supplementary Table S4). For *GSTM1* and *GSTT1* null genotypes, we considered studies that scored ≥10 as high quality; for *GSTP1* IIe105Val, studies scoring ≥12 were deemed as high quality.

### Statistical analysis

We used crude ORs and 95% CIs to estimate the associations between GST (M1, T1, and P1 IIe105Val) polymorphisms and leukemia risk. The Q statistic and I^2^ value were carried out to evaluate heterogeneity (Higgins et al., 2003). Only a random-effect model was used because the pooled results were same when I^2^ = 0% using random-effect and fixed-effect models (Der Simonian and Laird, 2015). We performed ORs with the corresponding 95% CIs following the genetic models. In GSTM1 and GSTT1 null genotypes, we used null vs. present model to calculate the pooled ORs with their 95% CIs. In GSTP1 IIe105Val, five genetic models were used (Val/Val vs. IIe/IIe, IIe/Val vs. IIe/IIe, Val/Val vs. IIe/IIe + IIe/Val, Val/Val + IIe/Val vs. IIe/IIe, and Val vs. IIe). In the combination of GSTM1 present/null and GSTT1 present/null, we applied the following six genetic models: model 1: M1 present/T1 null vs. M1 present/T1 present, model 2: M1 null/T1 present vs. M1 present/T1 present, model 3: M1 null/T1 null vs. M1 present/T1 present, model 4: All one risk genotypes vs. M1 present/T1 present, model 5: All risk genotypes vs. M1 present/T1 present, and model 6: M1 null/T1 null vs. M1 present/T1 present + M1 present/T1 null + M1 null/T1 present in the analysis of the data. The combination of GSTM1 present/null and GSTP1 IIe105Val was also used for the six genetic models, model 1: M1 null/P1 IIe/IIe vs. M1 present/P1 IIe/IIe, model 2: M1 present/P1 Val* vs. M1 present/P1 IIe/IIe, model 3: (M1 null/P1 IIe/IIe + M1 present/P1 Val*) vs. M1 present/P1 IIe/IIe, model 4: M1 null/P1 Val* vs. M1 present/P1 IIe/IIe, model 5: All risk genotypes vs. M1 present/P1 IIe/IIe, and model 6: M1 null/P1 Val* vs. (M1 present/P1 IIe/IIe + M1 null/P1 IIe/IIe + M1 Present/P1 Val*). There were six genetic models used in the combination of GSTT1 present/null and GSTP1 IIe105Val: model 1: T1 null/P1 IIe/IIe vs. T1 present/P1 IIe/IIe, model 2: T1 present/P1 Val* vs. T1 present/P1 IIe/IIe, model 3: = (T1 null/P1 IIe/IIe + T1 present/P1 Val*) vs. T1 present/P1 IIe/IIe, model 4: T1 null/P1 Val* vs. T1 present/P1 IIe/IIe, model 5: All risk genotypes vs. T1 present/P1 IIe/IIe, and model 6: T1 null/P1 Val* vs. (T1 present/P1 IIe/IIe + T1 null/P1 IIe/IIe + T1 Present/P1 Val*). In the combination of GSTM1 present/null, GSTT1 present/null, and GSTP1 IIe105Val, the following genetic models were employed: model 1: M1 null/T1 present/P1 IIe/IIe vs. M1 present/T1 present/P1 IIe/IIe, model 2: M1 present/T1 null/P1 IIe/IIe vs. M1 present/T1 present/P1 IIe/IIe, model 3: M1 present/T1 present/P1 Val 1 vs. M1 present/T1 present/P1 IIe/IIe, model 4: all one high-risk genotype vs. M1 present/T1 present/P1 IIe/IIe, model 5: M1 null/T1 null/P1 IIe/IIe vs. M1 present/T1 present/P1 IIe/IIe, model 6: M1 null/T1 present/P1 Val 1 vs. M1 present/T1 present/P1 IIe/IIe, model 7: M1 present/T1 null/P1 Val1 vs. M1 present/T1 present/P1 IIe/IIe, model 8: all two high-risk genotype vs. M1 present/T1 present/P1 IIe/IIe, model 9: M1 null/T1 null/P1 Val 1 vs. M1 present/T1 present/P1 IIe/IIe, and model 10: M1 null/T1 null/P1 Val 1 vs. M1 present/T1 present/P1 IIe/IIe + all one high-risk genotype + all two high-risk genotypes. Moreover, a metaregression analysis was used to explore sources of heterogeneity (Baker et al., 2009). Sensitivity analysis was conducted by excluding low-quality and Hardy–Weinberg disequilibrium (HWD) in control studies. The Hardy–Weinberg equilibrium (HWE) was checked using Chi-square goodness-of-fit test, which was deemed as HWE in controls if *p* ≥ 0.05. Begg’s funnel plot (Begg and Mazumdar, 1994) and Egger’s test (Egger et al., 1997) were carried out to verify publication bias. Furthermore, we applied the FPRP (Wacholder et al., 2004), BFDP (Wakefield, 2007), and Venice criteria (Ioannidis et al., 2008) to appraise the credibility of statistically significant associations. All statistical analyses were performed using Stata 12.0 software in the current study.

## Results

### Search results and study characteristics

**FIGURE 1:**
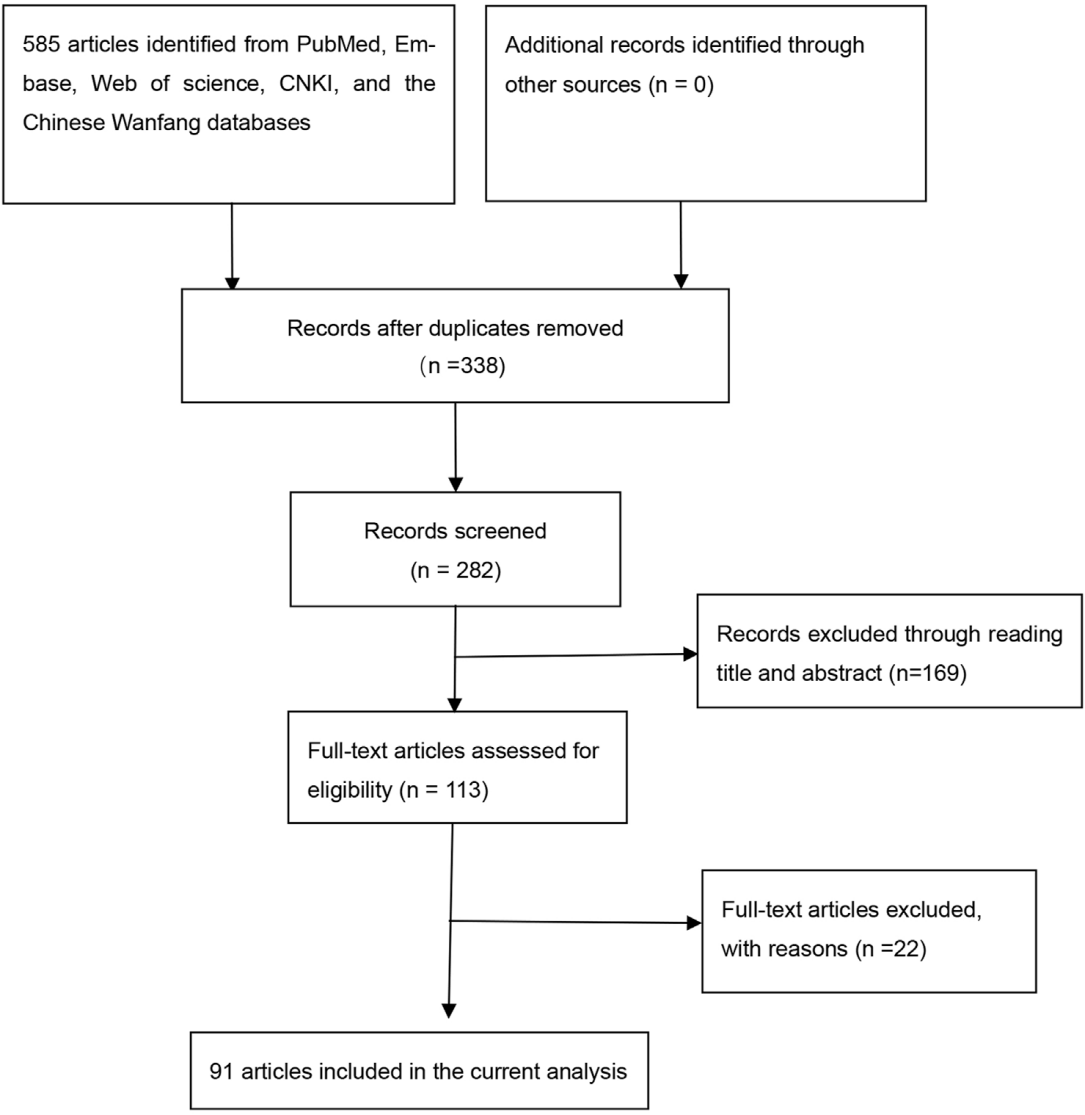
Flow diagram for the literature search.

Overall, 91 articles (Supplemental References 1–91) were eligible (Figure 1), and Supplementary Tables S1–S3 show the characteristics and scores of each study. Multiple eligible studies were included in one article. Therefore, there were 98 eligible studies (13,477 leukemia cases and 22,523 controls, Table 1) on the *GSTM1* present/null polymorphism, 89 eligible studies (12,357 leukemia cases and 20,636 controls, Table 2) on the *GSTT1* present/null polymorphism, 34 studies (5,391 leukemia cases and 8,729 controls, Table 3) on the GSTP1 IIe105Val polymorphism, 25 studies (3,522 leukemia cases and 4,974 controls, Table 4) belonging to the combined effects of the *GSTM1* and *GSTT1* polymorphisms, six studies (737 leukemia cases and 995 controls, Table 5) describing the combined *GSTM1* and *GSTP1* effects, five studies (645 leukemia cases and 845 controls, Table 6) on the combined *GSTT1* and *GSTP1* effects, and seven studies (1,036 leukemia cases and 1,418 controls, Table 7) belonging to the combined effects of the three aforementioned polymorphisms with leukemia risk.

**TABLE 1:** Meta-analysis of the association of GSTM1 polymorphism with risk of leukemia.

Variable	n	Cases/Controls	Test of association	Test of heterogeneity		Model
			OR (95%CI)	P_h_	I^2^ (%)	
Overall	98	13477/22523	1.28 (1.17–1.40)	<0.001	68.3	Random-effect
Ethnicity						
Indian	14	1600/2465	1.25 (0.89–1.77)	<0.001	84.6	Random-effect
Asian	24	3265/6028	1.50 (1.29–1.73)	0.002	51.2	Random-effect
Caucasian	47	7466/11124	1.17 (1.07–1.28)	<0.001	46.0	Random-effect
African	6	662/886	1.99 (1.30–3.94)	0.006	69.0	Random-effect
Age group						
Adults	37	5811/9440	1.26 (1.11–1.43)	<0.001	65.6	Random-effect
Children	31	4377/7321	1.42 (1.23–1.64)	<0.001	64.4	Random-effect
Adults and Children	25	2688/5205	1.10 (0.89–1.37)	<0.001	76.6	Random-effect
Type of control						
HC	65	7,442/11989	1.29 (1.15–1.44)	<0.001	66.6	Random-effect
NBDC	32	5978/10282	1.29 (1.13–1.48)	<0.001	71.9	Random-effect
Matching						
Yes	23	3819/5389	1.36 (1.12–1.65)	<0.001	77.7	Random-effect
No	75	9658/17134	1.25 (1.14–1.38)	<0.001	63.7	Random-effect
Type of leukemia						
AML	33	5530/10043	1.20 (1.04–1.38)	<0.001	71.1	Random-effect
ALL	41	5082/7895	1.44 (1.25–1.65)	<0.001	66.8	Random-effect
CML	20	2079/3426	1.17 (0.93–1.46)	<0.001	71.0	Random-effect
Sensitivity analysis						
Quality score≥10						
Overall	54	9420/15146	1.18 (1.07–1.30)	<0.001	65.6	Random-effect
Ethnicity						
Indian	10	1133/1690	1.04 (0.71–1.52)	<0.001	81.6	Random-effect
Asian	11	2323/4122	1.17 (1.05–1.31)	0.870	0.0	Random-effect
Caucasian	25	5293/7774	1.17 (1.06–1.30)	0.008	45.5	Random-effect
African	5	628/683	2.01 (1.23–3.30)	0.003	75.1	Random-effect
Age group						
Adults	28	5011/7,863	1.31 (1.15–1.50)	<0.001	63.2	Random-effect
Children	10	2282/3652	1.21 (1.06–1.39)	0.196	27.0	Random-effect
Adults and Children	14	1892/3455	0.90 (0.71–1.14)	<0.001	74.0	Random-effect
Type of control						
HC	36	5433/7693	1.21 (1.05–1.39)	<0.001	69.4	Random-effect
NBDC	18	4114/7581	1.14 (0.99–1.30)	0.002	56.8	Random-effect
Matching						
Yes	21	3525/4887	1.26 (1.05–1.52)	<0.001	73.5	Random-effect
No	33	6023/10387	1.13 (1.02–1.27)	<0.001	57.7	Random-effect
Type of leukemia						
AML	21	4598/8072	1.12 (0.97–1.28)	<0.001	63.7	Random-effect
ALL	16	2626/3754	1.22 (1.01–1.46)	<0.001	63.7	Random-effect
CML	14	1551/2417	1.23 (0.92–1.65)	<0.001	76.3	Random-effect

HC, healthy control; NBDC, nonblood disease control; AML, acute myeloid leukemia; ALL, acute lymphoblastic leukemia; and CML, chronic myeloid leukemia.

**TABLE 2:** Meta-analysis of the association of GSTT1 polymorphism with -risk of leukemia.

Variable	n	Cases/Controls	Test of association	Test of heterogeneity		Model
			OR (95%CI)	P_h_	I^2^ (%)	
Overall	89	12357/20636	1.46 (1.32–1.60)	<0.001	62.5	Random-effect
Ethnicity						
Indian	14	1600/2465	1.74 (1.27–2.38)	<0.001	71.9	Random-effect
Asian	24	3265/6028	1.30 (1.16–1.46)	0.140	24.2	Random-effect
Caucasian	38	6346/9237	1.37 (1.17–1.59)	<0.001	65.0	Random-effect
African	6	662/886	2.08 (1.32–3.26)	0.011	66.5	Random-effect
Age group						
Adults	37	5811/9440	1.55 (1.32–1.82)	<0.001	69.6	Random-effect
Children	27	3521/6123	1.24 (1.09–1.43)	0.028	37.2	Random-effect
Adults and Children	20	2424/4516	1.59 (1.27–1.99)	<0.001	67.1	Random-effect
Type of control						
HC	57	6522/10286	1.45 (1.28–1.66)	<0.001	63.7	Random-effect
NBDC	31	5778/10105	1.46 (1.26–1.69)	<0.001	62.7	Random-effect
Matching						
Yes	23	8272/14543	1.80 (1.44–2.24)	<0.001	74.8	Random-effect
No	66	4085/6093	1.35 (1.22–1.49)	<0.001	51.7	Random-effect
Type of leukemia						
AML	30	4851/9092	1.41 (1.19–1.66)	<0.001	67.7	Random-effect
ALL	37	4665/7,215	1.33 (1.16–1.53)	<0.001	53.0	Random-effect
CML	19	2068/3298	1.88 (1.47–2.41)	<0.001	64.5	Random-effect
Sensitivity analysis						
Quality score≥10						
Overall	52	8710/14300	1.52 (1.34–1.72)	<0.001	66.9	Random-effect
Ethnicity						
Indian	11	1225/1840	1.53 (1.08–2.17)	<0.001	69.6	Random-effect
Asian	11	2323/4122	1.15 (1.01–1.31)	0.239	21.5	Random-effect
Caucasian	22	4363/6650	1.64 (1.37–1.96)	<0.001	64.5	Random-effect
African	5	628/683	2.12 (1.26–3.58)	0.007	71.9	Random-effect
Age group						
Adults	28	5011/7,863	1.58 (1.33–1.89)	<0.001	71.3	Random-effect
Children	8	1552/2705	1.35 (1.00–1.82)	0.005	65.1	Random-effect
Adults and Children	14	1784/3428	1.45 (1.14–1.83)	0.001	61.5	Random-effect
Type of control						
HC	34	4704/6746	1.56 (1.31–1.86)	<0.001	71.0	Random-effect
NBDC	18	4006/7,554	1.45 (1.23–1.72)	0.002	56.7	Random-effect
Matching						
Yes	21	3525/4887	1.73 (1.37–2.17)	<0.001	73.6	Random-effect
No	31	5185/9413	1.41 (1.23–1.62)	<0.001	59.2	Random-effect
Type of leukemia						
AML	19	3937/7,249	1.35 (1.12–1.63)	<0.001	68.3	Random-effect
ALL	16	2449/3603	1.49 (1.19–1.88)	<0.001	64.2	Random-effect
CML	14	1551/2417	1.93 (1.44–2.59)	<0.001	64.9	Random-effect

HC, healthy control; NBDC, nonblood disease control; AML, acute myeloid leukemia; ALL, acute lymphoblastic leukemia; and CML, chronic myeloid leukemia.

**TABLE 3:** Meta-analysis of the association of GSTP1 polymorphism with risk of leukemia.

Variable	n (Cases/Controls)	Val/Val vs. lle/lle		lle/Val vs. lle/lle		Val/Val vs. lle/lle + lle/Val		Val/Val + lle/Val vs. lle/lle		Val vs. lle	
		OR (95%CI)	P_h_/I^2^(%)	OR (95%CI)	P_h_/I^2^(%)	OR (95%CI)	P_h_/I^2^(%)	OR (95%CI)	P_h_/I^2^(%)	OR (95%CI)	P_h_/I^2^(%)
Overall	34(5391/8729)	1.77 (1.40–2.24)	0.000/59.8	1.24 (1.08–1.43)	0.000/67.7	1.59 (1.29–1.95)	0.100/50.9	1.32 (1.15–1.53)	0.000/72.6	1.31 (1.16–1.47)	0.000/75.0
Ethnicity											
Indian	10(1392/2094)	3.01 (1.60–5.66)	0.000/76.8	1.28 (1.08–1.53)	0.167/30.3	2.65 (1.47–4.79)	0.000/74.8	1.45 (1.17–1.80)	0.013/57.2	1.47 (1.19–1.80)	0.000/72.1
Asian	10(1895/3338)	1.27 (0.98–1.66)	0.381/6.5	1.25 (0.91–1.72)	0.000/78.8	1.22(0.96–1.55)	0.799/0.0	1.30 (0.96–1.76)	0.000/80.1	1.26 (1.00–1.60)	0.000/78.1
Caucasian	12(1791/2976)	1.49 (1.10–2.01)	0.073/40.2	1.28 (0.98–1.68)	0.000/73.8	1.31 (1.04–1.65)	0.294/15.3	1.32 (1.02–1.72)	0.000/75.6	1.28 (1.05–1.55)	0.000/74.0
Age group											
Adults	14(1392/2094)	1.39 (1.06–1.82)	0.102/34.1	1.17 (0.95–1.43)	0.000/67.3	1.27 (1.01–1.61)	0.233/20.2	1.20 (0.99–1.46)	0.000/68.1	1.18 (1.02–1.37)	0.000/64.6
Children	8(1392/2094)	1.68 (1.10–2.58)	0.115/39.6	1.14 (0.88–1.46)	0.038/52.8	1.60 (1.11–2.32)	0.223/25.8	1.23 (0.94–1.60)	0.012/61.3	1.26 (1.00–1.58)	0.004/66.3
Adults and Children	9(1392/2094)	3.25 (1.61–6.53)	0.000/76.8	1.64 (1.16–2.31)	0.000/73.7	2.65 (1.41–5.02)	0.000/72.9	1.82 (1.29–2.57)	0.000/77.3	1.72 (1.29–2.30)	0.000/80.4
Type of control											
HC	21(2699/3569)	2.38 (1.66–3.41)	0.000/61.2	1.27 (1.05–1.54)	0.000/65.2	2.12 (1.53–2.94)	0.001/55.3	1.39 (1.15–1.69)	0.000/69.6	1.40 (1.19–1.63)	0.000/71.5
NBDC	13 (2692/5160)	1.19 (0.99–1.44)	0.395/5.1	1.19 (0.96–1.48)	0.000/71.0	1.14 (0.96–1.36)	0.836/0.0	1.22 (0.99–1.50)	0.000/73.9	1.17 (1.00–1.38)	0.000/72.9
Matching											
Yes	14 (2510/3287)	1.37 (1.07–1.76)	0.203/23.1	1.07 (0.95–1.20)	0.665/0.0	1.30 (1.03–1.64)	0.244/19.2	1.12 (1.00–1.24)	0.594/0.0	1.13 (1.03–1.24)	0.384/6.1
No	20(2881/5442)	2.13 (1.49–3.06)	0.000/69.0	1.36 (1.08–1.71)	0.000/78.4	1.86 (1.36–2.54)	0.000/60.6	1.47 (1.17–1.86)	0.000/81.7	1.44 (1.19–1.74)	0.000/82.8
Type of leukemia											
AML	13 (2225/4667)	1.57 (1.10–2.24)	0.000/66.4	1.37 (1.02–1.84)	0.000/83.1	1.37 (1.01–1.84)	0.008/55.1	1.42 (1.06–1.89)	0.000/84.8	1.34 (1.07–1.68)	0.000/85.1
ALL	12 (1540/2445)	1.90 (1.28–2.81)	0.018/52.1	1.15 (0.96–1.38)	0.103/36.0	1.77 (1.25–2.53)	0.051/44.0	1.26 (1.03–1.53)	0.020/51.4	1.29 (1.08–1.53)	0.003/61.0
CML	6 (926/810)	1.29 (1.08–1.53)	0.009/67.3	1.13 (0.84–1.53)	0.068/51.3	2.13 (1.08–4.24)	0.028/60.1	1.23 (0.86–1.76)	0.007/68.7	1.27 (0.92–1.74)	0.001/75.3
Sensitivity analysis											
HWE											
Overall	24 (3781/6111)	1.58 (1.27–1.95)	0.118/26.3	1.18 (1.02–1.37)	0.000/59.0	1.45 (1.21–1.74)	0.361/7.3	1.25 (1.07–1.45)	0.000/64.1	1.24 (1.10–1.40)	0.000/64.4
Ethnicity											
Indian	7 (842/1350)	1.83 (1.11–3.03)	0.033/56.3	1.24 (1.01–1.51)	0.333/12.7	1.67 (1.05–2.64)	0.055/51.3	1.34 (1.06–1.69)	0.110/42.1	1.33 (1.06–1.66)	0.017/61.2
Asian	6 (1376/2603)	1.14 (0.79–1.64)	0.728/0.0	1.04 (0.83–1.30)	0.129/41.4	1.15 (0.80–1.64)	0.799/0.0	1.06 (0.85–1.32)	0.113/43.9	1.06 (0.89–1.26)	0.165/36.3
Caucasian	9 (1250/1837)	1.70 (1.23–2.34)	0.257/21.0	1.30 (0.94–1.80)	0.000/75.8	1.50 (1.14–1.95)	0.524/0.0	1.39 (1.01–1.90)	0.000/76.4	1.36 (1.08–1.70)	0.000/72.8
Age group											
Adults	10 (1835/3530)	1.39 (1.07–1.81)	0.493/0.0	1.20 (0.93–1.56)	0.000/72.1	1.31 (1.02–1.69)	0.717/0.0	1.24 (0.97–1.60)	0.000/72.8	1.22 (1.01–1.46)	0.001/67.7
Children	8 (1124/1633)	1.68 (1.10–2.58)	0.115/39.6	1.14 (0.88–1.46)	0.038/52.8	1.60 (1.11–2.32)	0.223/25.8	1.23 (0.94–1.60)	0.012/61.3	1.26 (1.00–1.58)	0.004/66.3
Adults and Children	5 (622/848)	1.81 (0.95–3.44)	0.038/60.6	1.31 (0.99–1.72)	0.195/33.9	1.59 (0.91–2.79)	0.084/51.3	1.39 (1.01–1.92)	0.054/57.0	1.34 (1.00–1.80)	0.013/68.2
Type of control											
HC	17 (2099/2775)	1.86 (1.38–2.50)	0.083/34.2	1.21 (0.98–1.50)	0.000/65.8	1.71 (1.33–2.21)	0.266/16.0	1.31 (1.05–1.62)	0.000/68.9	1.31 (1.11–1.55)	0.000/68.1
NBDC	7 (1682/3336)	1.21 (0.93–1.59)	0.930/0.0	1.08 (0.93–1.26)	0.289/18.4	1.16 (0.89–1.50)	0.985/0.0	1.11 (0.95–1.29)	0.223/27.0	1.09 (0.97–1.22)	0.296/17.6
Matching											
Yes	9 (1546/1696)	1.51 (1.11–2.05)	0.787/0.0	1.12 (0.96–1.30)	0.929/0.0	1.43 (1.07–1.93)	0.742/0.0	1.17 (1.01–1.35)	0.908/0.0	1.18 (1.05–1.32)	0.796/0.0
No	15 (2235/4415)	1.66 (1.21–2.27)	0.023/47.1	1.24 (0.98–1.56)	0.000/73.6	1.50 (1.15–1.94)	0.142/28.8	1.31 (1.03–1.66)	0.000/76.9	1.29 (1.07–1.55)	0.000/76.6
Type of leukemia											
AML	7 (1141/2762)	1.18 (0.86–1.62)	0.424/0.0	1.17 (0.80–1.69)	0.000/80.0	1.13 (0.83–1.54)	0.717/0.0	1.18 (0.83–1.69)	0.000/80.1	1.14 (0.89–1.48)	0.000/75.5
ALL	10 (1345/2047)	1.60 (1.15–2.22)	0.197/26.8	1.10 (0.91–1.31)	0.180/28.8	1.53 (1.14–2.06)	0.280/17.8	1.18 (0.97–1.43)	0.075/42.4	1.21 (1.03–1.43)	0.025/52.6
Quality score≥12											
Overall	18 (3430/5975)	1.62 (1.25–2.11)	0.018/45.7	1.16 (0.99–1.36)	0.001/58.6	1.49 (1.18–1.89)	0.067/35.6	1.23 (1.05–1.44)	0.000/64.0	1.23 (1.09–1.40)	0.000/65.5
Ethnicity											
Caucasian	6 (1064/2009)	1.54 (1.09–2.17)	0.220/28.7	1.41 (0.98–2.04)	0.001/76.9	1.28 (1.01–1.64)	0.489/0.0	1.48 (1.04–2.10)	0.001/77.2	1.38 (1.08–1.77)	0.002/73.1
Indian	7 (1010/1448)	2.15 (1.22–3.76)	0.013/62.6	1.17(0.95–1.43)	0.231/25.9	1.97 (1.16–3.35)	0.019/60.3	1.29 (1.03–1.63)	0.079/47.1	1.32 (1.07–1.64)	0.016/61.6
Age group											
Adults	11 (1392/2094)	1.42 (1.07–1.88)	0.134/33.1	1.19 (0.97–1.46)	0.001/67.4	1.29 (1.02–1.64)	0.279/17.3	1.24 (1.01–1.51)	0.000/68.9	1.21 (1.04–1.41)	0.001/65.5
Type of control											
HC	13 (1856/2372)	1.94 (1.37–2.76)	0.044/44.2	1.21 (0.97–1.50)	0.002/62.1	1.77 (1.29–2.44)	0.088/36.9	1.30 (1.05–1.62)	0.001/64.8	1.31 (1.10–1.55)	0.001/64.0
NBDC	5 (1574/3603)	1.14 (0.89–1.46)	0.735/0.0	1.05 (0.88–1.27)	0.152/40.4	1.12 (0.88–1.42)	0.901/0.0	1.08 (0.90–1.30)	0.112/46.6	1.07 (0.93–1.23)	0.150/40.8
Matching											
Yes	11 (2226/2966)	1.45 (1.11–1.90)	0.283/16.9	1.06 (0.94–1.20)	0.942/0.0	1.41 (1.08–1.83)	0.263/18.9	1.12 (0.99–1.25)	0.919/0.0	1.14 (1.04–1.25)	0.762/0.0
No	7 (1204/3009)	1.81 (1.07–3.06)	0.006/67.1	1.34 (0.90–2.00)	0.000/83.3	1.58 (1.02–2.46)	0.034/55.9	1.43 (0.96–2.15)	0.000/85.6	1.37 (1.00–1.89)	0.000/85.5
Type of leukemia											
AML	7 (1461/3684)	1.11 (0.88–1.41)	0.470/0.0	1.21 (0.88–1.65)	0.000/78.4	1.08 (0.86–1.35)	0.880/0.0	1.21 (0.89–1.64)	0.000/79.2	1.14 (0.92–1.43)	0.000/75.5
CML	5 (855/743)	3.17 (1.89–5.32)	0.308/16.7	1.18 (0.85–1.64)	0.052/57.5	2.80 (1.79–4.39)	0.489/0.0	1.35 (0.94–1.94)	0.014/68.0	1.41 (1.05–1.89)	0.013/68.3
HWE and Quality score≥12											
Overall	16 (2750/4705)	1.63 (1.24–2.13)	0.081/35.2	1.20 (1.00–1.44)	0.001/61.8	1.49 (1.18–1.88)	0.223/20.2	1.27 (1.06–1.53)	0.000/66.8	1.26 (1.08–1.46)	0.000/67.7
Ethnicity											
Indian	6 (750/1200)	1.91 (1.07–3.40)	0.020/62.6	1.23 (0.97–1.55)	0.236/26.5	1.74 (1.03–2.96)	0.037/57.8	1.34 (1.02–1.76)	0.066/51.7	1.34 (1.04–1.74)	0.009/67.4
Caucasian	5 (644/987)	1.87 (1.28–2.74)	0.649/0.0	1.55 (1.02–2.34)	0.007/71.9	1.59 (1.11–2.30)	0.734/0.0	1.63 (1.12–2.37)	0.012/68.7	1.50 (1.17–1.91)	0.057/56.4
Age group											
Adults	9 (1735/3430)	1.38 (1.05–1.82)	0.408/3.2	1.27 (0.97–1.66)	0.000/72.2	1.28 (0.98–1.66)	0.705/0.0	1.30 (1.00–1.70)	0.000/73.9	1.24 (1.02–1.52)	0.001/70.4
Type of control											
HC	12 (1596/2124)	1.83 (1.29–2.58)	0.072/40.3	1.23 (0.97–1.57)	0.002/63.5	1.66 (1.23–2.25)	0.169/28.2	1.33 (1.05–1.68)	0.001/66.7	1.32 (1.09–1.59)	0.001/66.8
Matching											
Yes	9 (1546/1696)	1.51 (1.11–2.05)	0.787/0.0	1.12 (0.96–1.30)	0.929/0.0	1.43 (1.07–1.93)	0.742/0.0	1.17 (1.01–1.35)	0.908/0.0	1.18 (1.05–1.32)	0.796/0.0
No	7 (1204/3009)	1.81 (1.07–3.06)	0.006/67.1	1.34 (0.90–2.00)	0.000/83.3	1.58 (1.02–2.46)	0.034/55.9	1.43 (0.96–2.15)	0.000/85.6	1.37 (1.00–1.89)	0.000/85.5
Type of leukemia											
AML	6 (1041/2662)	1.16 (0.82–1.64)	0.355/9.5	1.27 (0.84–1.90)	0.000/81.5	1.08 (0.78–1.48)	0.792/0.0	1.26 (0.85–1.88)	0.000/82.4	1.18 (0.88–1.57)	0.000/79.3

HC, healthy control; NBDC, nonblood disease control; AML, acute myeloid leukemia; ALL, acute lymphoblastic leukemia; and CML, chronic myeloid leukemia.

**TABLE 4:** Meta-analysis of the combined effects of GSTM1 present/null and GSTT1 present/null on leukemia risk.

Variable	N (Case/Control)	Model 1		Model 2		Model 3		Model 4		Model 5		Model 6	
		OR (95%CI)	P_h_/I^2^(%)	OR (95%CI)	P_h_/I^2^(%)	OR (95%CI)	P_h_/I^2^(%)	OR (95%CI)	P_h_/I^2^(%)	OR (95%CI)	P_h_/I^2^(%)	OR (95%CI)	P_h_/I^2^(%)
Overall	25 (3522/4974)	1.66 (1.37–2.00)	0.077/30.3	1.11 (0.93–1.33)	0.000/60.4	2.44 (1.86–3.21)	0.002/51.2	1.29 (1.11–1.50)	0.001/52.2	1.44 (1.25–1.66)	0.002/51.5	2.16 (1.65–2.81)	0.000/55.4
Ethnicity													
Indian	5 (555/829)	1.92 (1.18–3.12)	0.075/52.9	0.87 (0.54–1.40)	0.017/66.6	3.16 (1.90–5.25)	0.519/0.0	1.18 (0.76–1.85)	0.006/72.4	1.32 (0.83–2.10)	0.002/76.1	2.83 (1.73–4.64)	0.759/0.0
Asian	5 (1000/1148)	1.43 (1.04–1.97)	0.274/22.1	1.34 (0.99–1.81)	0.146/41.4	2.47 (1.55–3.95)	0.051/57.5	1.35 (1.02–1.80)	0.120/45.3	1.57 (1.20–2.05)	0.129/44.0	2.05 (1.40–3.00)	0.090/50.3
Caucasian	10 (1506/1916)	1.65 (1.14–2.39)	0.087/40.6	1.15 (0.92–1.43)	0.101/38.6	1.98 (1.16–3.37)	0.005/61.5	1.30 (1.05–1.60)	0.082/41.3	1.37 (1.17–1.61)	0.317/13.7	1.71 (0.94–3.09)	0.000/71.8
Age group													
Adults	15 (2424/2884)	1.44 (1.18–1.76)	0.600/0.0	1.27(1.04–1.54)	0.013/50.7	2.51 (1.71–3.68)	0.001/60.0	1.34 (1.15–1.57)	0.087/35.3	1.50 (1.29–1.74)	0.104/33.0	2.26 (1.53–3.33)	0.000/65.8
Adults and children	5 (488/1112)	1.63 (0.87–3.07)	0.014/68.0	0.75 (0.50–1.12)	0.122/45.0	2.05 (0.96–4.37)	0.063/55.1	0.99 (0.66–1.49)	0.044/59.2	1.10 (0.71–1.71)	0.016/67,2	1.94 (1.04–4.36)	0.131/43.6
Type of control													
HC	15(1693/2058)	1.73 (1.31–2.30)	0.060/39.2	1.02 (0.76–1.38)	0.000/69.8	2.59 (1.71–3.93)	0.012/51.1	1.27 (1.00–1.62)	0.000/63.9	1.45 (1.16–1.80)	0.001/60.3	2.33 (1.52–3.58)	0.002/59.7
NBDC	9 (1772/2671)	1.60 (1.22–2.10)	0.215/25.7	1.29 (1.11–1.50)	0.466/0.0	2.31 (1.56–3.43)	0.021/55.7	1.36 (1.18–1.57)	0.421/1.6	1.49 (1.25–1.78)	0.147/33.9	1.86 (1.33–2.61)	0.059/46.7
Matching													
Yes	11 (1958/2382)	1.60 (1.29–1.99)	0.493/0.0	1.13 (0.85–1.50)	0.000/70.1	2.57 (1.61–4.12)	0.002/64.2	1.31 (1.09–1.58)	0.075/41.0	1.46 (1.24–1.73)	0.129/33.8	2.33 (1.44–3.76)	0.000/69.6
No	14 (1564/2592)	1.67 (1.24–2.27)	0.023/48.0	1.09 (0.86–1.40)	0.013/51.6	2.38 (1.70–3.33)	0.076/37.6	1.28 (1.00–1.63)	0.002/60.7	1.43 (1.13–1.80)	0.001/62.1	2.07 (1.52–2.81)	0.084/36.5
Type of leukemia													
AML	6 (1176/1859)	1.47 (0.96–2.26)	0.084/48.5	1.24 (0.97–1.58)	0.202/31.2	2.15 (1.35–3.43)	0.049/55.1	1.29 (0.99–1.69)	0.088/47.8	1.41 (1.09–1.82)	0.095/46.7	1.85 (1.22–2.80)	0.069/51.1
ALL	7 (670/1060)	2.15 (1.43–3.23)	0.125/39.9	1.19 (0.77–1.85)	0.008/65.2	2.79 (1.47–5.30)	0.052/52.0	1.52 (1.13–2.05)	0.094/44.5	1.66 (1.25–2.20)	0.106/42.7	2.23 (1.20–4.14)	0.036/55.4
CML	11 (1234/1613)	1.54 (1.18–2.01)	0.375/7.2	1.01 (0.72–1.42)	0.000/69.7	2.58 (1.57–4.24)	0.024/51.4	1.19 (0.92–1.56)	0.004/61.4	1.37 (1.06–1.77)	0.003/62.5	2.41 (1.45–4.00)	0.012/55.8
Sensitivity analysis													
Quality score													
≥10													
Overall	21 (3105/4266)	1.56 (1.28–1.91)	0.132/26.3	1.12 (0.92–1.37)	0.000/61.6	2.41 (1.76–3.29)	0.001/55.3	1.27 (1.09–1.48)	0.007/48.3	1.42 (1.22–1.65)	0.003/51.7	2.17 (1.61–2.94)	0.001/57.8
Ethnicity													
Indian	5 (555/829)	1.92 (1.18–3.12)	0.075/52.9	0.87 (0.54–1.40)	0.017/66.6	3.16 (1.90–5.25)	0.519/0.0	1.18 (0.76–1.85)	0.006/72.4	1.32 (0.83–2.10)	0.002/76.1	2.83 (1.73–4.64)	0.759/0.0
Caucasian	8 (1121/1352)	1.38 (0.96–1.98)	0.260/21.4	1.21 (0.95–1.53)	0.100/41.7	1.80 (0.96–3.37)	0.008/63.6	1.28 (1.04–1.57)	0.160/33.6	1.34 (1.13–1.58)	0.341/11.5	1.63 (0.83–3.21)	0.001/71.5
Age group													
Adults	14 (2317/2754)	1.43 (1.16–1.76)	0.527/0.0	1.31 (1.08–1.60)	0.020/48.8	2.40 (1.61–3.58)	0.002/59.9	1.37 (1.16–1.61)	0.089/35.8	1.51 (1.29–1.77)	0.078/37.4	2.13 (1.43–3.18)	0.000/64.7
Adults and children	5 (488/1112)	1.63 (0.87–3.07)	0.014/68.0	0.75 (0.50–1.12)	0.122/45.0	2.05 (0.96–4.37)	0.063/55.1	0.99 (0.66–1.49)	0.044/59.2	1.10 (0.71–1.71)	0.016/67,2	1.94 (1.04–4.36)	0.131/43.6
Type of Control													
HC	13 (1539/1826)	1.63 (1.21–2.18)	0.103/34.9	1.06 (0.76–1.47)	0.000/73.5	2.56 (1.60–4.10)	0.009/55.0	1.25 (0.97–1.61)	0.001/64.6	1.41 (1.11–1.80)	0.001/63.6	2.39 (1.50–3.80)	0.004/58.7
NBDC	8 (1566/2440)	1.47 (1.12–1.94)	0.306/15.7	1.24 (1.06–1.45)	0.693/0.0	2.17 (1.43–3.29)	0.030/54.9	1.30 (1.12–1.50)	0.724/0.0	1.41 (1.19–1.66)	0.294/17.2	1.85 (1.27–2.69)	0.045/51.3
Matching													
Yes	11 (1958/2382)	1.60 (1.29–1.99)	0.493/0.0	1.13 (0.85–1.50)	0.000/70.1	2.57 (1.61–4.12)	0.002/64.2	1.31 (1.09–1.58)	0.078/41.0	1.46 (1.24–1.73)	0.129/33.8	2.33 (1.44–3.76)	0.000/69.6
No	10 (1147/1884)	1.51 (1.04–2.19)	0.044/48.0	1.11 (0.84–1.47)	0.032/50.8	2.26 (1.45–3.53)	0.051/46.7	1.22 (0.93–1.61)	0.012/57.2	1.37 (1.03–1.82)	0.002/65.1	2.04 (1.40–2.98)	0.107/37.7
Type of leukemia													
ALL	6 (623/958)	1.92 (1.28–2.86)	0.213/29.6	1.22 (0.76–1.96)	0.004/70.7	3.10 (1.48–6.49)	0.036/58.1	1.43 (1.07–1.91)	0.144/39.3	1.59 (1.18–2.14)	0.100/45.8	2.66 (1.38–5.15)	0.053/54.1
CML	10 (1127/1483)	1.55 (1.15–2.09)	0.292/16.4	1.04 (0.73–1.51)	0.000/71.8	2.39 (1.37–4.16)	0.020/54.3	1.22 (0.91–1.64)	0.003/64.6	1.38 (1.03–1.83)	0.002/66.2	2.21 (1.26–3.87)	0.012/57.7

Model 1, M1 present/T1 null vs. M1 present/T1 present; Model 2, M1 null/T1 present vs. M1 present/T1 present; Model 3, M1 null/T1 null vs. M1 present/T1 present; Model 4, all one risk genotypes vs. M1 present/T1 present; Model 5, all risk genotypes vs. M1 present/T1 present; Model 6, M1 null/T1 null vs. M1 present/T1 present + M1 present/T1 null + M1 null/T1 present; HC, healthy control; NBDC, nonblood disease control; AML, acute myeloid leukemia; ALL, acute lymphoblastic leukemia; and CML, chronic myeloid leukemia.

**TABLE 5:** Meta-analysis of the combined effects of GSTM1 present/null and GSTP1 IIe105Val on leukemia risk.

Variable	Sample size	Model 1		Model 2		Model 3		Model 4		Model 5		Model 6	
		OR (95%CI)	P_h_/I^2^(%)	OR (95%CI)	P_h_/I^2^(%)	OR (95%CI)	P_h_/I^2^(%)	OR (95%CI)	P_h_/I^2^(%)	OR (95%CI)	P_h_/I^2^(%)	OR (95%CI)	P_h_/I^2^(%)
Overall	6 (737/995)	0.83 (0.55–1.26)	0.038/57.5	1.16 (0.74–1.84)	0.017/63.9	1.02 (0.74–1.39)	0.063/52.2	1.95 (1.35–2.80)	0.272/21.5	1.19 (0.90–1.58)	0.100/45.9	1.95 (1.37–2.77)	0.208/30.4
Ethnicity													
Indian	4 (492/750)	0.75 (0.39–1.45)	0.015/71.4	1.26 (0.74–2.13)	0.021/69.2	1.05 (0.65–1.68)	0.018/70.2	1.72 (1.10–2.70)	0.211/33.5	1.18 (0.77–1.79)	0.030/66.4	1.65 (1.14–2.40)	0.292/19.6
Type of control													
HC	5 (645/845)	0.74 (0.49–1.12)	0.081/51.9	1.14 (0.65–2.02)	0.008/71.1	0.97 (0.68–1.38)	0.052/57.5	1.82 (1.21–2.74)	0.249/25.9	1.13 (0.83–1.54)	0.097/49.1	1.88 (1.23–2.89)	0.143/41.8
Matching													
Yes	3 (395/395)	0.72 (0.37–1.41)	0.033/70.6	0.82 (0.43–1.57)	0.147/47.8	0.78 (0.51–1.21)	0.142/48.7	1.89 (0.90–3.96)	0.113/54.1	0.99 (0.63–1.56)	0.087/59.0	2.20 (1.25–3.89)	0.204/37.1
No	3 (342/600)	0.97 (0.53–1.76)	0.123/52.3	1.53 (0.91–2.56)	0.097/57.2	1.31 (0.97–1.77)	0.581/0.0	2.07 (1.34–3.20)	0.413/0.0	1.44 (1.08–1.92)	0.771/0.0	1.76 (1.05–2.96)	0.178/42.1
Type of leukemia													
ALL	3 (342/600)	0.83 (0.34–2.03)	0.008/79.5	0.98 (0.70–1.38)	0.403/0.0	0.91 (0.53–1.57)	0.038/69.3	1.86 (1.01–3.43)	0.125/51.9	1.08 (0.63–1.84)	0.028/72.0	1.92 (1.30–2.83)	0.498/0.0
CML	3 (395/395)	0.86 (0.60–1.24)	0.377/0.0	1.34 (0.51–3.48)	0.015/76.1	1.17 (0.83–1.63)	0.306/15.6	2.08 (1.27–3.40)	0.363/1.4	1.34 (1.00–1.92)	0.705/0.0	2.00 (0.90–4.46)	0.055/65.4
Sensitivity analysis													
HWE and Quality score													
≥10	6 (737/995)	0.83 (0.55–1.26)	0.038/57.5	1.16 (0.74–1.84)	0.017/63.9	1.02 (0.74–1.39)	0.063/52.2	1.95 (1.35–2.80)	0.272/21.5	1.19 (0.90–1.58)	0.100/45.9	1.95 (1.37–2.77)	0.208/30.4

Model 1, M1 null/P1 IIe/IIe vs. M1 present/P1 IIe/IIe; Model 2, M1 present/P1 Val* vs. M1 present/P1 IIe/IIe; Model 3, (M1 null/P1 IIe/IIe + M1 present/P1 Val*) vs. M1 present/P1 IIe/IIe; Model 4 = M1 null/P1 Val* vs. M1 present/P1 IIe/IIe; Model 5, All risk genotypes vs. M1 present/P1 IIe/IIe; Model 6, M1 null/P1 Val* vs. (M1 present/P1 IIe/IIe + M1 null/P1 IIe/IIe + M1 Present/P1 Val*); HC, healthy control; NBDC, nonblood disease controls; AML, acute myeloid leukemia; ALL, acute lymphoblastic leukemia; and CML, chronic myeloid leukemia.

**TABLE 6:** Meta-analysis of the combined effects of GSTT1 present/null and GSTP1 IIe105Val on leukemia risk.

Variable	Sample size	Model 1		Model 2		Model 3		Model 4		Model 5		Model 6	
		OR (95%CI)	P_h_/I^2^	OR (95%CI)	P_h_/I^2^	OR (95%CI)	P_h_/I^2^	OR (95%CI)	P_h_/I^2^	OR (95%CI)	P_h_/I^2^	OR (95%CI)	P_h_/I^2^
Overall	5 (645/845)	1.56 (0.76–3.19)	0.009/70.6	1.49 (0.97–2.28)	0.032/62.2	1.50 (1.04–2.15)	0.041/59.8	4.24 (2.49–7.24)	0.596/0.0	1.70 (1.30–2.22)	0.207/32.2	3.31 (1.85–5.91)	0.320/14.8
Ethnicity													
Indian	3 (400/600)	1.90 (0.99–3.66)	0.086/59.3	1.45 (0.72–2.92)	0.006/80.4	1.65 (1.05–2.59)	0.072/61.9	4.39 (2.51–7.68)	0.741/0.0	1.91 (1.45–2.50)	0.365/0.8	3.39 (1.94–5.94)	0.338/7.8
Type of control													
HC	5 (645/845)	1.56 (0.76–3.19)	0.009/70.6	1.49 (0.97–2.28)	0.032/62.2	1.50 (1.04–2.15)	0.041/59.8	4.24 (2.49–7.24)	0.596/0.0	1.70 (1.30–2.22)	0.207/32.2	3.31 (1.85–5.91)	0.320/14.8
Matching													
Yes	3 (395/395)	1.44 (0.48–4.35)	0.032/70.8	1.16 (0.65–2.08)	0.082/60.0	1.18 (0.76–1.83)	0.135/50.1	4.61 (1.64–12.97)	0.301/16.8	1.40 (1.04–1.89)	0.368/0.0	4.15 (0.78–7.37)	0.278/21.9
Type of leukemia													
CML	3 (395/395)	0.88 (0.41–1.88)	0.218/34.3	1.91 (1.35–2.68)	0.441/0.0	1.49 (0.89–2.51)	0.059/64.6	3.29 (1.37–7.89)	0.361/1.9	1.61 (1.05–2.47)	0.133/50.4	2.40 (1.21–14.26)	0.231/31.8
Sensitivity analysis													
HWE and Quality score≥10													
Overall	5 (645/845)	1.56 (0.76–3.19)	0.009/70.6	1.49 (0.97–2.28)	0.032/62.2	1.50 (1.04–2.15)	0.041/59.8	4.24 (2.49–7.24)	0.596/0.0	1.70(1.30–2.22)	0.207/32.2	3.31 (1.85–5.91)	0.320/14.8

Model 1, T1 null/P1 IIe/IIe vs. T1 present/P1 IIe/IIe; Model 2, T1 present/P1 Val* vs. T1 present/P1 IIe/IIe; Model 3, (T1 null/P1 IIe/IIe + T1 present/P1 Val*) vs. T1 present/P1 IIe/IIe; Model 4, T1 null/P1 Val* vs. T1 present/P1 IIe/IIe; Model 5, all risk genotypes vs. T1 present/P1 IIe/IIe; Model 6, T1 null/P1 Val* vs. (T1 present/P1 IIe/IIe + T1 null/P1 IIe/IIe + T1 Present/P1 Val*); HB, hospital-based studies; PB, population-based studies; HC, healthy control; NBDC, nonblood disease controls; AML, acute myeloid leukemia; ALL, acute lymphoblastic leukemia; and CML, chronic myeloid leukemia.

**TABLE 7:** Meta-analysis of the combined effects of GSTM1 present/null, GSTT1 present/null, and GSTP1 IIe105Val on leukemia risk.

Variable	Sample size	Model 1		Model 2		Model 3		Model 4		Model 5		Model 6		Model 7		Model 8		Model 9		Model 10	
		OR (95%CI)	P_h_/I^2^(%)	OR (95%CI)	P_h_/I^2^(%)	OR (95%CI)	P_h_/I^2^(%)	OR (95%CI)	P_h_/I^2^(%)	OR (95%CI)	P_h_/I^2^(%)	OR (95%CI)	P_h_/I^2^(%)	OR (95%CI)	P_h_/I^2^(%)	OR (95%CI)	P_h_/I^2^(%)	OR (95%CI)	P_h_/I^2^(%)	OR (95%CI)	P_h_/I^2^(%)
Overall	7 (1036/1418)	0.93 (0.72–1.21)	0.945/0.0	1.38 (0.92–2.07)	0.261/22.0	1.12 (0.86–1.47)	0.723/0.0	1.07 (0.87–1.33)	0.486/0.0	0.81 (0.23–2.92)	0.000/90.1	1.18 (0.78–1.79)	0.092/44.9	0.95 (0.57–1.61)	0.148/36.8	1.09 (0.71–1.68)	0.006/66.5	2.04 (0.89–4.70)	0.007/66.2	1.87 (0.97–3.62)	0.038/55.1
Sensitivity analysis																					
Quality score																					
>8	7 (1036/1418)	ACT	0.945/0.0	1.38 (0.92–2.07)	0.261/22.0	1.12 (0.86–1.47)	0.723/0.0	1.07 (0.87–1.33)	0.486/0.0	0.81 (0.23–2.92)	0.000/90.1	1.18 (0.78–1.79)	0.092/44.9	0.95 (0.57–1.61)	0.148/36.8	1.09 (0.71–1.68)	0.006/66.5	2.04 (0.89–4.70)	0.007/66.2	1.87 (0.97–3.62)	0.038/55.1
HWE																					
Yes	6 (603/705)	0.90 (0.68–1.18)	0.987/0.0	1.38 (0.83–2.31)	0.179/34.4	1.11 (0.83–1.48)	0.604/0.0	1.05 (0.83–1.32)	0.402/2.2	0.69 (0.16–2.94)	0.000/90.0	1.12 (0.70–1.79)	0.076/49.9	0.81 (0.45–1.44)	0.225/28.0	1.00 (0.62–1.62)	0.009/67.4	1.85 (0.69–4.96)	0.009/67.3	1.79 (0.79–4.08)	0.032/59.1

Model 1 = M1 null/T1 present/P1 IIe/IIe vs. M1 present/T1 present/P1 IIe/IIe, Model 2 = M1 present/T1 null/P1 IIe/IIe vs. M1 present/T1 present/P1 IIe/IIe, Model 3 = M1 present/T1 present/P1 Val 1 vs. M1 present/T1 present/P1 IIe/IIe, Model 4 = all one high-risk genotype vs. M1 present/T1 present/P1 IIe/IIe, Model 5 = M1 null/T1 null/P1 IIe/IIe vs. M1 present/T1 present/P1 IIe/IIe, Model 6 = M1 null/T1 present/P1 Val 1 vs. M1 present/T1 present/P1 IIe/IIe, Model 7 = M1 present/T1 null/P1 Val1 vs. M1 present/T1 present/P1 IIe/IIe, Model 8 = all two high-risk genotype vs. M1 present/T1 present/P1 IIe/IIe, Model 9 = M1 null/T1 null/P1 Val 1 vs. M1 present/T1 present/P1 IIe/IIe, and Model 10 = M1 null/T1 null/P1 Val 1 vs. M1 present/T1 present/P1 IIe/IIe + all one high-risk genotype + all two high-risk genotypes.

### Quantitative synthesis

**FIGURE 2:**
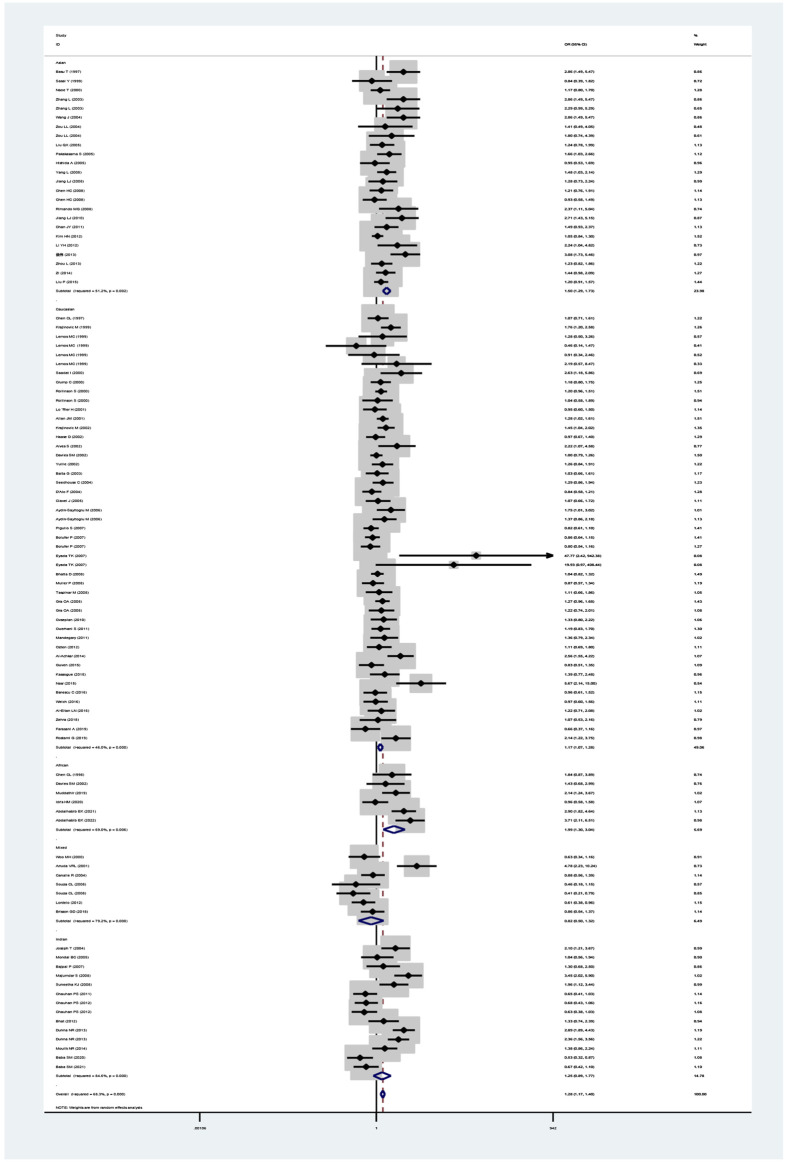
Forest plot for the association between GSTM1 polymorphism and leukemia risk in ethnicity subgroup analysis.

The *GSTM1* null genotype significantly added leukemia risk in the overall analysis (OR = 1.28, 95% CI: 1.17–1.40, Table 1 and Figure 2) of Asians (OR = 1.50, 95% CI: 1.29–1.73), Caucasians (OR = 1.17, 95% CI: 1.07–1.28), and Africans (OR = 1.99, 95% CI: 1.30–3.94). However, it showed that the GSTM1 null genotype did not affect leukemia risk in Indians (OR = 1.25, 95% CI: 0.89–1.77). Moreover, similar association was also found in other subgroup analyses, such as in adult leukemia, child leukemia, AML, ALL, and so on (Table 1).

**FIGURE 3:**
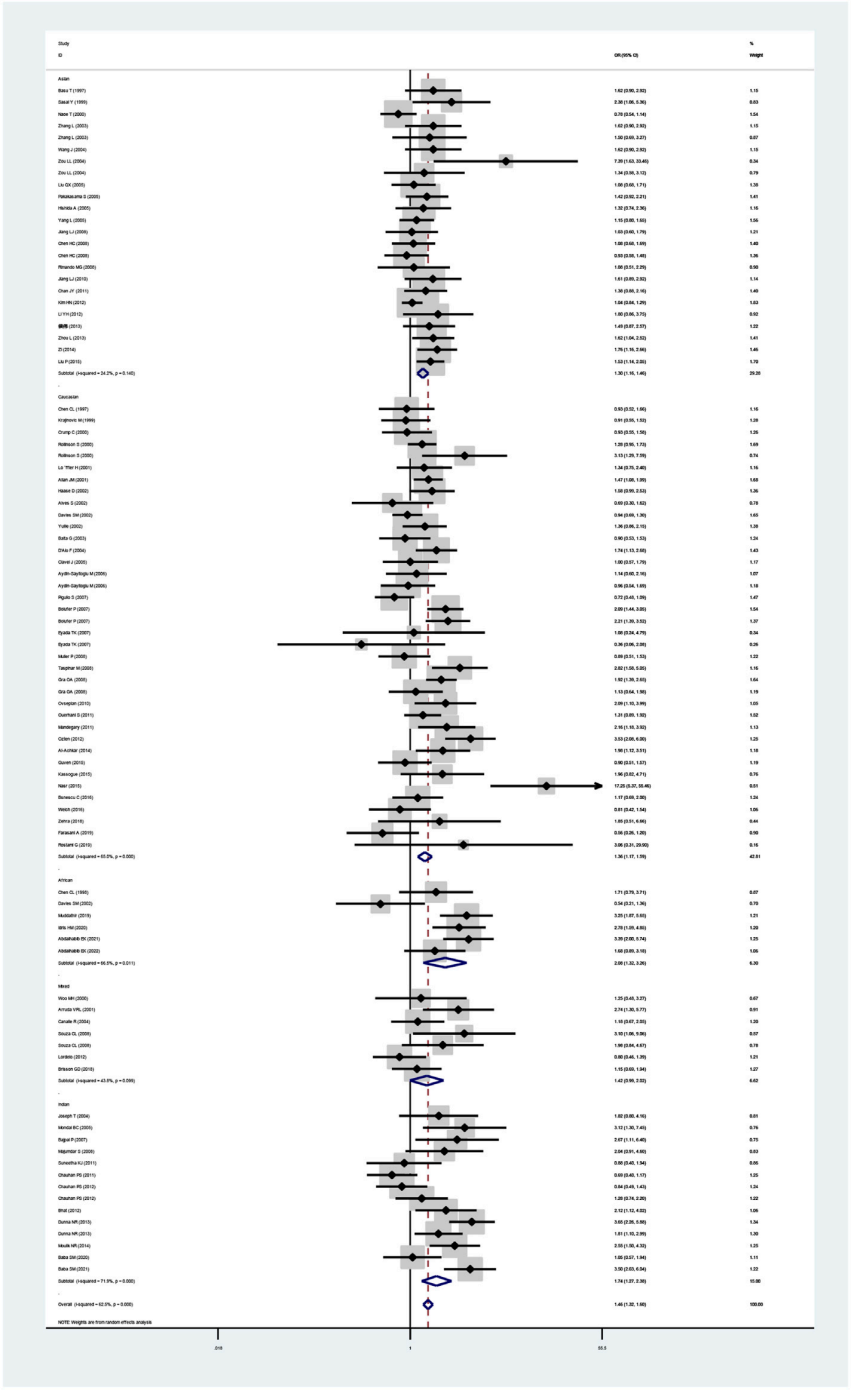
Forest plot for the association between GSTT1 polymorphism and leukemia risk in ethnicity subgroup analysis.

The GSTT1 null genotype added leukemia risk in the overall population (OR = 1.46, 95% CI: 1.32–1.60, Table 2 and Figure 3). Moreover, an increased risk of leukemia was also found in Indians (OR = 1.74, 95% CI: 1.27–2.38), Asians (OR = 1.30, 95% CI: 1.16–1.46), Caucasians (OR = 1.37, 95% CI: 1.17–1.59), and Africans (OR = 2.08, 95% CI: 1.32–3.26) (Table 2; Figure 3). Similarly, the significantly increased risk of leukemia was also observed in adult leukemia, child leukemia, AML, ALL, and CML, and so on (Table 2).

**FIGURE 4:**
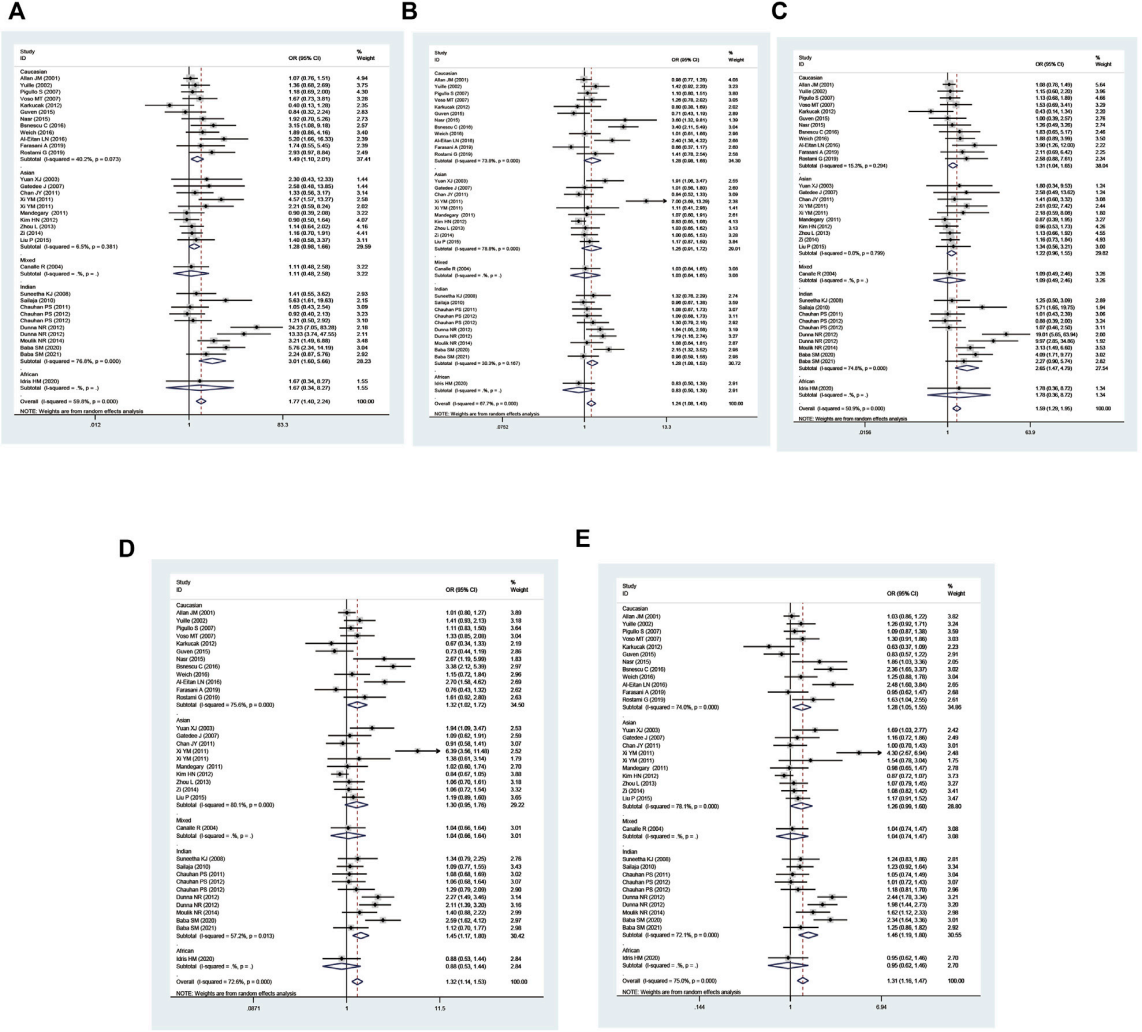
Forest plot for the association between GSTP1 polymorphism and leukemia risk in ethnicity subgroup analysis [**(A)**: Val/Val vs. Ile/Ile; **(B)** Ile/Val vs. Ile/Ile; **(C)** Val/Val vs. Ile/Ile + Ile/Val; **(D)** Val/Val + Ile/Val vs. Ile/Ile; and **(E)** Val vs. Ile].

The GSTP1 IIe105Val polymorphism yielded a significantly increased leukemia risk in overall population (Val/Val vs. IIe/IIe: OR = 1.77, 95% CI = 1.40–2.24; IIe/Val vs. IIe/IIe: OR = 1.24, 95% CI = 1.08–1.43; Val/Val vs. IIe/IIe + IIe/Val: OR = 1.59, 95% CI = 1.29–1.95; Val/Val + IIe/Val vs. IIe/IIe: OR = 1.32, 95% CI = 1.15–1.53; and Val vs. IIe: OR = 1.31, 95% CI = 1.16–1.47, Table 3 and Figure 4). Moreover, the GSTP1 IIe105Val polymorphism was associated with increased leukemia risk in Indians (Val/Val vs. IIe/IIe: OR = 3.01, 95% CI = 1.60–5.66; IIe/Val vs. IIe/IIe: OR = 1.28, 95% CI = 1.08–1.53; Val/Val vs. IIe/IIe + IIe/Val: OR = 2.65, 95% CI = 1.47–4.79; Val/Val + IIe/Val vs. IIe/IIe: OR = 1.45, 95% CI = 1.17–1.80; and Val vs. IIe: OR = 1.47, 95% CI = 1.19–1.80) and in Caucasians (Val/Val vs. IIe/IIe: OR = 1.49, 95% CI = 1.10–2.01; Val/Val vs. IIe/IIe + IIe/Val: OR = 1.31, 95% CI = 1.04–1.65; Val/Val + IIe/Val vs. IIe/IIe: OR = 1.32, 95% CI = 1.02–1.72; and Val vs. IIe: OR = 1.28, 95% CI = 1.05–1.55). Similarly, the significantly increased risk of leukemia was also observed in adult leukemia, child leukemia, AML, ALL, CML, etc. (Table 3).

**FIGURE 5:**
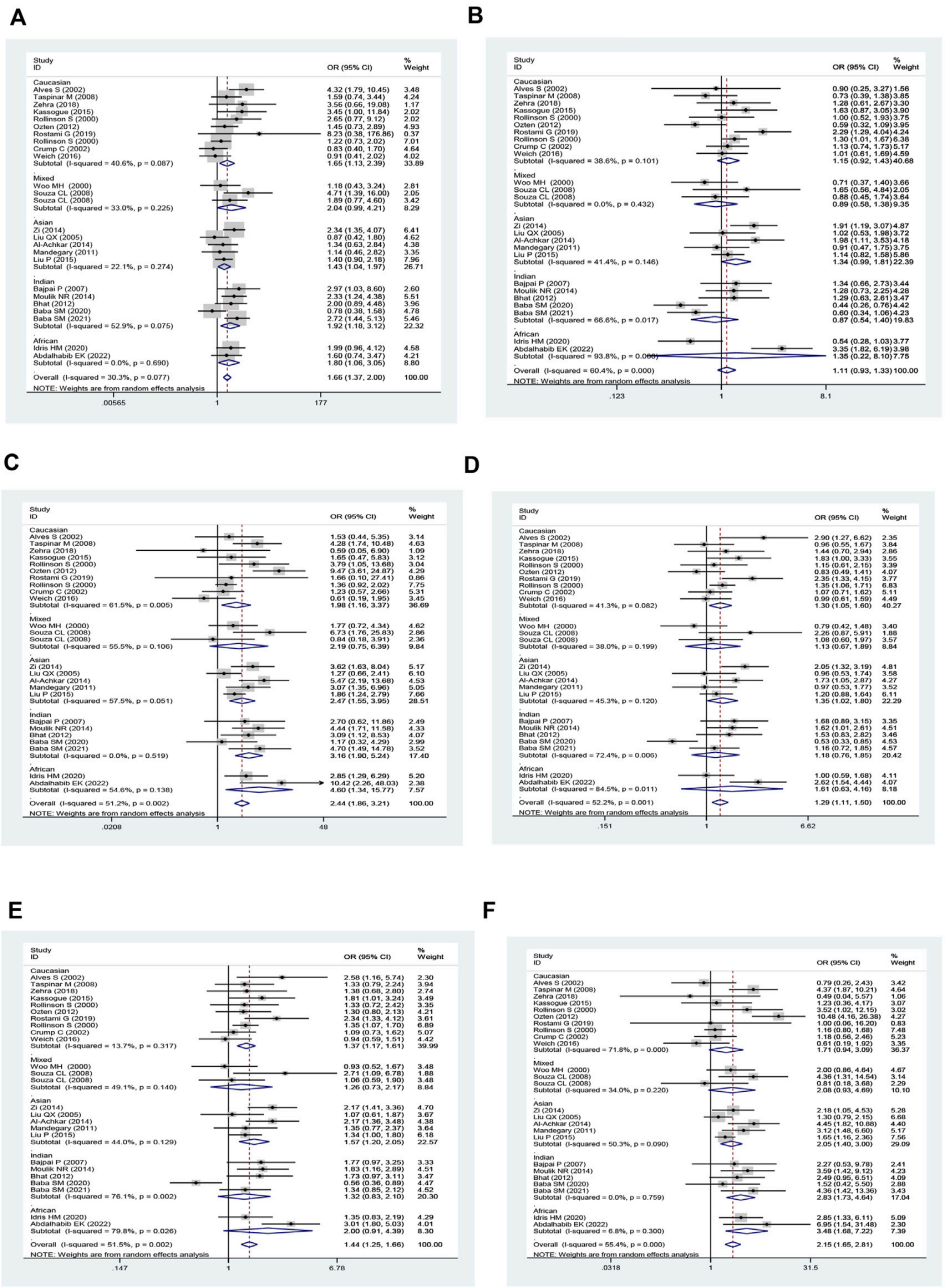
Forest plot of the association between combined effects of GSTM1 present/null and GSTT1 present/null polymorphisms and leukemia risk in ethnicity subgroup analysis [**(A)**: Model 1; **(B)** Model 2; **(C)** Model 3; **(D)** Model 4; **(E)** Model 5; and **(F)** Model 6].

Combined *GSTM1* and *GSTT1* null genotypes were found to significantly increase leukemia risk in the overall analysis (M1 present/T1 null vs. M1 present/T1 present: OR = 1.66, 95% CI = 1.37–2.00; M1 null/T1 null vs. M1 present/T1 present: OR = 2.44, 95% CI = 1.86–3.21; all one risk genotypes vs. M1 present/T1 present: OR = 1.29, 95% CI = 1.11–1.50; all risk genotypes vs. M1 present/T1 present: OR = 1.44, 95% CI = 1.25–1.66; and M1 null/T1 null vs. M1 present/T1 present + M1 present/T1 null + M1 null/T1 present: OR = 2.16, 95% CI = 1.65–2.81; Table 4 and Figure 5). Moreover, there was a significantly increased leukemia risk in Indians (M1 present/T1 null vs. M1 present/T1 present: OR = 1.92, 95% CI = 1.18–3.12; M1 null/T1 null vs. M1 present/T1 present: OR = 3.16, 95% CI = 1.90–5.25; M1 null/T1 null vs. M1 present/T1 present + M1 present/T1 null + M1 null/T1 present: OR = 2.83, 95% CI = 1.73–4.64), Asians (M1 present/T1 null vs. M1 present/T1 present: OR = 1.43, 95% CI = 1.04–1.97; M1 null/T1 null vs. M1 present/T1 present: OR = 2.47, 95% CI = 1.55–3.95; all one risk genotypes vs. M1 present/T1 present: OR = 1.35, 95% CI = 1.02–1.80; all risk genotypes vs. M1 present/T1 present: OR = 1.57, 95% CI = 1.20–2.05; M1 null/T1 null vs. M1 present/T1 present + M1 present/T1 null + M1 null/T1 present: OR = 2.05, 95% CI = 1.40–3.00), and Caucasians (M1 present/T1 null vs. M1 present/T1 present: OR = 1.65, 95% CI = 1.14–2.39; M1 null/T1 null vs. M1 present/T1 present: OR = 1.98, 95% CI = 1.16–3.37; all one risk genotypes vs. M1 present/T1 present: OR = 1.30, 95% CI = 1.05–1.60; all risk genotypes vs. M1 present/T1 present: OR = 1.37, 95% CI = 1.17–1.61). Similar results were found in adult leukemia, AML, ALL, CML, and so on (Table 4).

**FIGURE 6:**
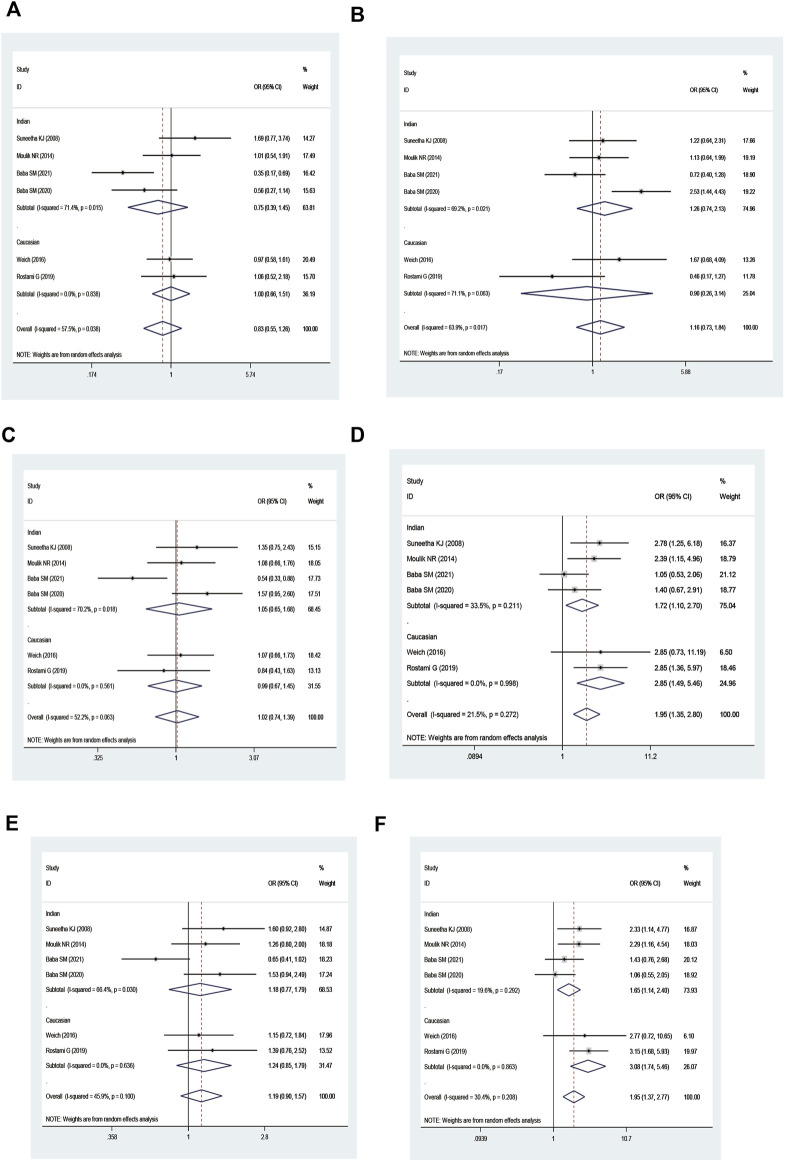
Forest plot of the association between combined effects of GSTM1 present/null and GSTP1 11e105Val polymorphisms and leukemia risk in ethnicity subgroup analysis [**(A)**: Model 1; **(B)** Model 2; **(C)** Model 3; **(D)** Model 4; **(E)** Model 5; and **(F)** Model 6].

An increased risk of leukemia was yielded on the combined GSTM1 and GSTP1 polymorphisms (M1 null/P1 Val* vs. M1 present/P1 IIe/IIe: OR = 1.95, 95% CI = 1.35–2.80; M1 null/P1 Val* vs. M1 present/P1 IIe/IIe + M1 null/P1 IIe/IIe + M1 Present/P1 Val*: OR = 1.95, 95% CI = 1.37–2.77; Table 5 and Figure 6) in overall analysis. Moreover, increased leukemia risk was also demonstrated in Indians (M1 null/P1 Val* vs. M1 present/P1 IIe/IIe: OR = 1.72, 95% CI = 1.10–2.70, M1 null/P1 Val* vs. M1 present/P1 IIe/IIe + M1 null/P1 IIe/IIe + M1 Present/P1 Val*: OR = 1.65, 95% CI = 1.14–2.40). Furthermore, a similar connection was also found in ALL, CML, and so on (Table 5).

**FIGURE 7:**
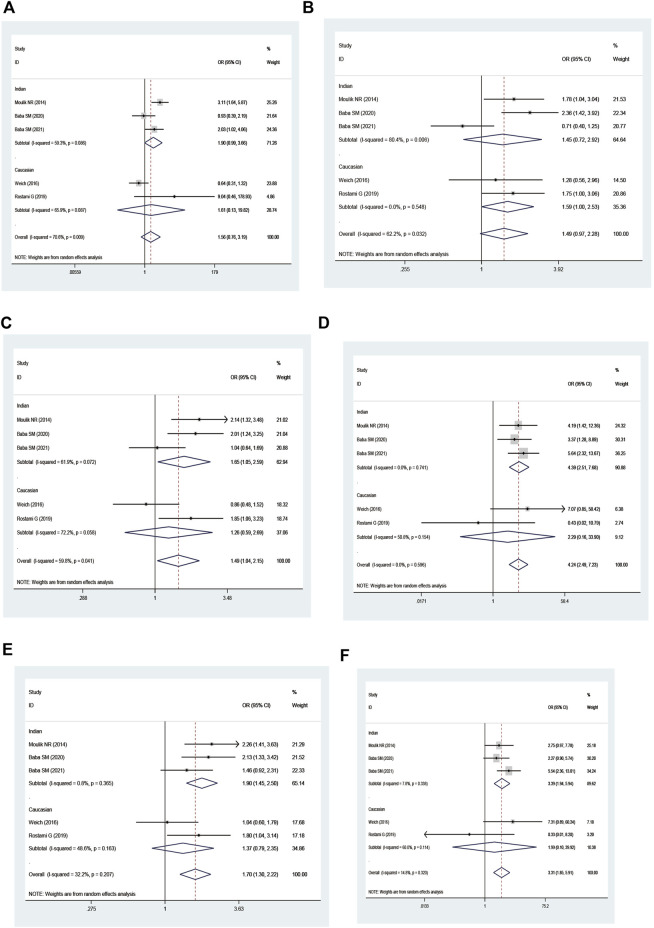
Forest plot of the association between the combined effects of GSTT1 present/null and GSTP1 11e105Val polymorphisms and leukemia risk in ethnicity subgroup analysis [**(A)**: Model 1; **(B)** Model 2; **(C)** Model 3; **(D)** Model 4; **(E)** Model 5; and **(F)** Model 6].

On combining GSTT1 and GSTP1 polymorphisms, there was a strong connection with leukemia risk in the overall analysis ((T1 null/P1 IIe/IIe + T1 present/P1 Val*) vs. T1 present/P1 IIe/IIe: OR = 1.50, 95% CI = 1.04–2.15; T1 null/P1 Val* vs. T1 present/P1 IIe/IIe: OR = 4.24, 95% CI = 2.49–7.24; all risk genotypes vs. T1 present/P1 IIe/IIe: OR = 1.70, 95% CI = 1.30–2.22; and T1 null/P1 Val* vs. (T1 present/P1 IIe/IIe + T1 null/P1 IIe/IIe + T1 Present/P1 Val*): OR = 3.31, 95% CI = 1.85–5.91) and increased risk of leukemia among Indians ((T1 null/P1 IIe/IIe + T1 present/P1 Val*) vs. T1 present/P1 IIe/IIe: OR = 1.65, 95% CI = 1.05–2.59; T1 null/P1 Val* vs. T1 present/P1 IIe/IIe: OR = 4.39, 95% CI = 2.51–7.68; all risk genotypes vs. T1 present/P1 IIe/IIe: OR = 1.91, 95% CI = 1.45–2.50; T1 null/P1 Val* vs. (T1 present/P1 IIe/IIe + T1 null/P1 IIe/IIe + T1 Present/P1 Val*): OR = 3.39, 95% CI = 1.94–5.94; Table 6 and Figure 7).

**FIGURE 8:**
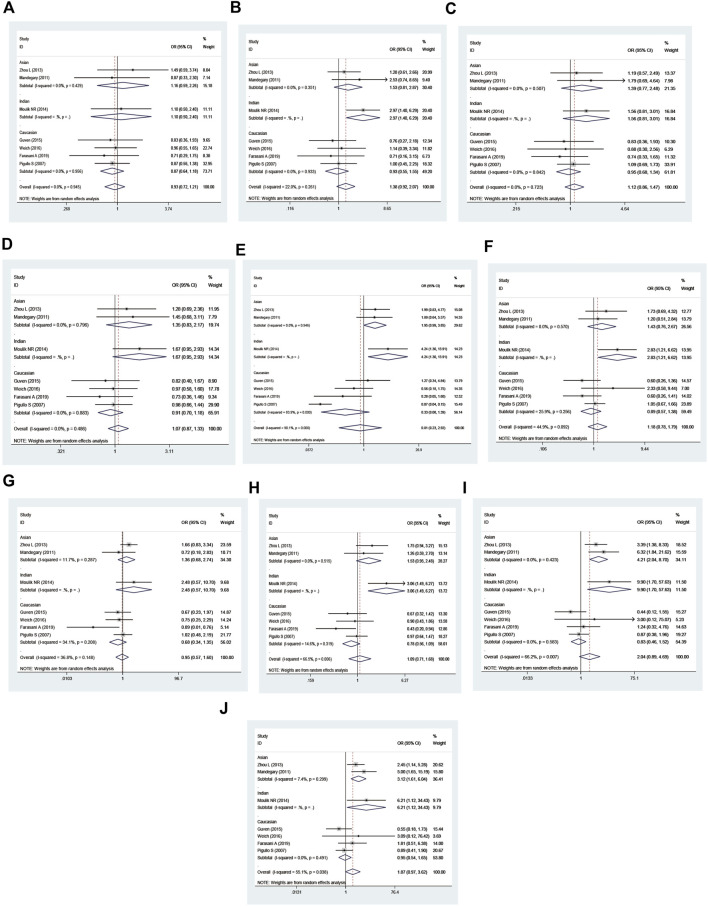
Forest plot of the association between the combined effects of GSTM1 present/null, GSTT1 present/null, and GSTP111e105Val polymorphisms and leukemia risk in the ethnicity subgroup analysis [**(A)**: Model 1; **(B)** Model 2; **(C)** Model 3; **(D)** Model 4; **(E)** Model 5; **(F)** Model 6; **(G)** Model 7; **(H)** Model 8; **(I)** Model 9; and **(J)** Model 10].

No significantly increased leukemia risk was observed in the three combined polymorphisms in the overall populations (Table 7; Figure 8).

### Heterogeneity and sensitivity analyses

The metaregression analysis showed that race (*p* = 0.000) and quality score (*p* = 0.038) were sources of heterogeneity for the *GSTM1* null genotype. For *GSTP1* IIe105Val polymorphism, in Val/Val vs. IIe/IIe + IIe/Val, type of controls (*p* = 0.002), matching studies (*p* = 0.023), and HWE (*p* = 0.005) were the heterogeneity sources. Similar results were observed in Val/Val vs. lle/lle + lle/Val where type of controls (*p* = 0.001), matching studies (*p* = 0.037), and HWE (*p* = 0.007) were the sources of heterogeneity. For the combined *GSTM1* and *GSTT1* polymorphisms, the sample size (model 1: *p* = 0.015) was the source of heterogeneity (Table 8). Three methods were performed to appraise the sensitivity analysis, and all results did not change (Tables 1–7), indicating that the present study was stable.

**TABLE 8:** Heterogeneity analysis in current meta-analysis.

Variables	Type of leukemia	Age group	Ethnicity	Sample size	Type of control	Matching	HWE	Quality score
P								
Genotype								
GSTM1	0.342	0.957	0.000	0.137	0.777	0.137	—	0.038
GSTT1	0.075	0.781	0.974	0.111	0.913	0.052	—	0.930
GSTP1 IIe105Val								
Val/Val vs. lle/lle	0.144	0.546	0.074	0.134	0.002	0.023	0.005	0.617
lle/Val vs. lle/lle	0.385	0.450	0.767	0.892	0.445	0.190	0.280	0.714
Val/Val vs. lle/lle + lle/Val	0.185	0.648	0.081	0.100	0.001	0.037	0.007	0.642
Val/Val+ lle/Val vs. lle/lle	0.341	0.525	0.575	0.706	0.244	0.098	0.142	0.829
Val vs. lle	0.328	0.616	0.463	0.528	0.106	0.064	0.073	0.878
The combined effects of GSTM1 and GSTT1 polymorphisms								
Model 1	0.648	0.067	0.432	0.015	0.622	0.212	—	0.478
Model 2	0.349	0.281	0.071	0.537	0.234	0.532	—	0.886
Model 3	0.702	0.917	0.792	0.686	0.739	0.714	—	0.699
Model 4	0.341	0.979	0.215	0.161	0.721	0.987	—	0.753
Model 5	0.402	0.939	0.124	0.268	0.850	0.974	—	0.644
Model 6	0.882	0.801	0.956	0.361	0.627	0.667	—	0.796

### Publication bias

**FIGURE 9:**
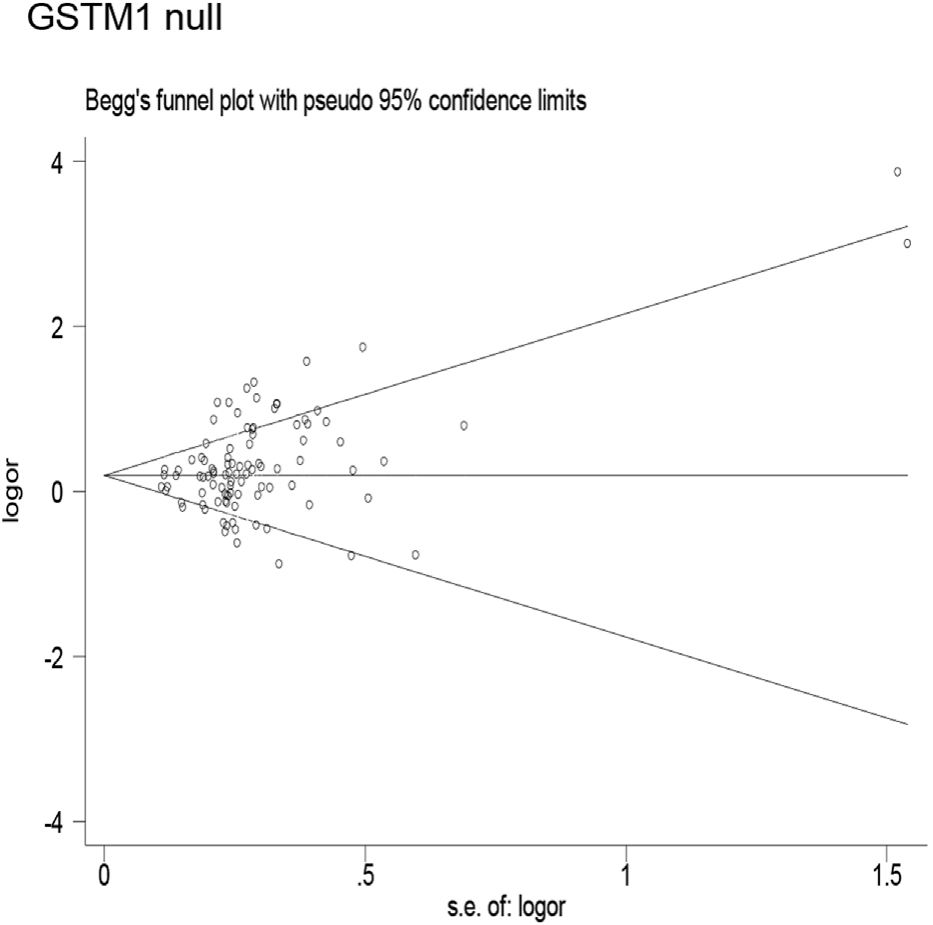
Begg’s funnel plot to assess publication bias.

**FIGURE 10:**
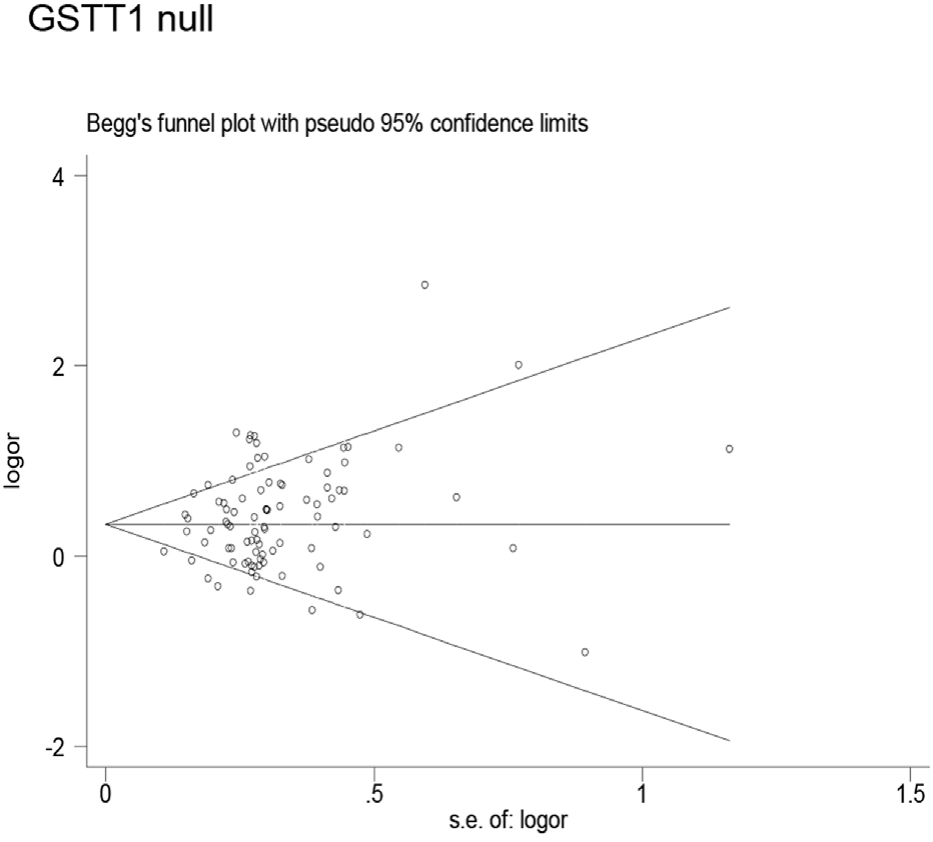
Begg’s funnel plot to assess publication bias.

**FIGURE 11:**
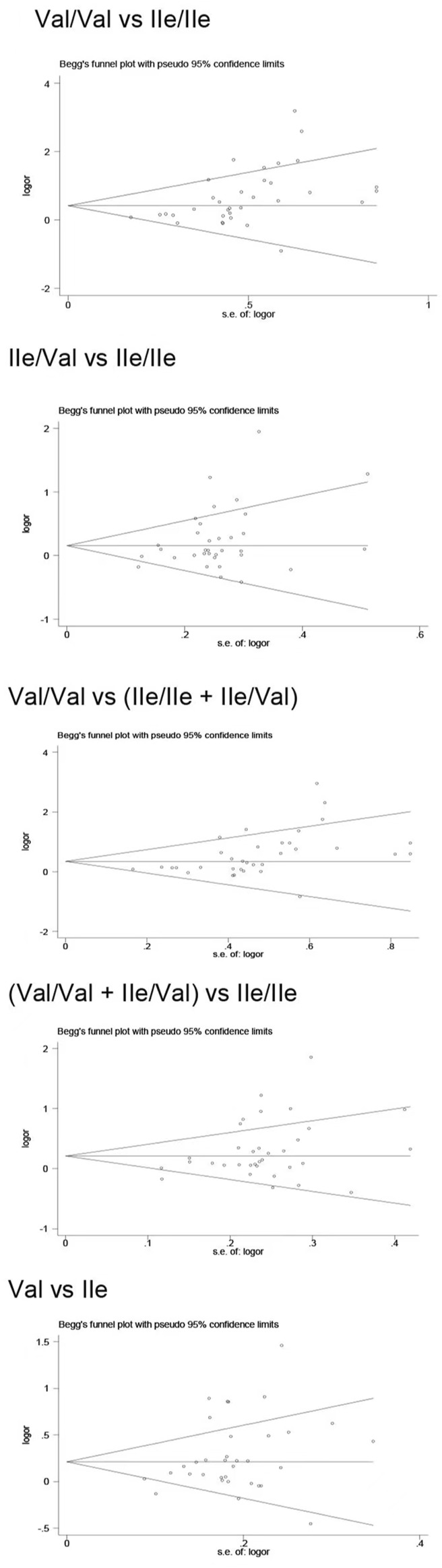
Begg’s funnel plot to assess publication bias.

Publication bias was found for the *GSTM1* null genotype (*p* = 0.003, Figure 9), GSTT1 null genotype (*p* = 0.041, Figure 10), and *GSTP1* IIe105Val (Val/Val vs. IIe/IIe: *p* = 0.001, IIe/Val vs. IIe/IIe: *p* = 0.030, Val/Val vs. IIe/IIe + IIe/Val: *p* = 0.020, Val/Val + IIe/Val vs. IIe/IIe: *p* = 0.022, Val vs. IIe: *p* = 0.033, Figure 11). Then, we used nonparametric “trim and fill” to adjust publication bias, and the results did not change (data not shown).

### Credibility of the positive results

The “reliable results” was defined as the positive results that met the following criteria (Theodoratou et al., 2012). First, these positive results were observed in at least two of the genetic models (exclude individual *GSTM1* and *GSTT1* polymorphisms with the risk of leukemia), second, FPRP <0.2 and BFDP <0.8, third, I^2^ < 50%, and fourth, statistical power >80%. Table 9 lists the credibility of the present meta-analysis on the individual and the composite effects of *GSTM1*, *GSTT1,* and *GSTP1* IIe105Val polymorphisms with the risk of leukemia. Only the GSTT1 null genotype with leukemia risk in Asians was considered as “positive” results (OR = 1.30, 95% CI = 1.16–1.46, I^2^ = 24.2%, statistical power = 0.992, FPRP = 0.009, and BFDP = 0.367). All other important connections were regarded as less-credible results, also shown in Table 9.

**TABLE 9:** Credibility of the current meta-analysis.

Variables	Model	OR (95%CI)	I2 (%)	Statistical power	Credibility	
					Prior probability of 0.001	
					FPRP	BFDP
GSTM1						
Overall	Null vs present	1.28 (1.17–1.40)	68.3	1.000	<0.001	0.006
Asian	Null vs present	1.50 (1.29–1.73)	51.2	0.500	<0.001	0.002
Caucasian	Null vs present	1.17 (1.07–1.28)	46.0	1.000	0.381	0.973
African	Null vs present	1.99 (1.30–3.94)	69.0	0.209	0.996	0.998
Adults	Null vs present	1.26 (1.11–1.43)	65.6	0.997	0.257	0.940
Children	Null vs present	1.42 (1.23–1.64)	64.4	0.772	0.002	0.096
HC	Null vs present	1.29 (1.15–1.44)	66.6	0.996	0.006	0.273
NBDC	Null vs present	1.29 (1.13–1.48)	71.9	0.984	0.222	0.924
Matching	Null vs present	1.36 (1.12–1.65)	77.7	0.840	0.684	0.981
Nonmatching	Null vs present	1.25 (1.14–1.38)	63.7	1.000	0.010	0.408
AML	Null vs present	1.20 (1.04–1.38)	71.1	0.999	0.914	0.997
ALL	Null vs present	1.44 (1.25–1.65)	66.8	0.722	<0.001	0.010
Sensitivity analysis						
Quality score ≥10						
Overall	Null vs present	1.16 (1.05–1.27)	62.2	1.000	0.569	0.986
Asian	Null vs present	1.17 (1.05–1.31)	0.0	1.000	0.866	0.996
Caucasian	Null vs present	1.17 (1.06–1.30)	45.5	1.000	0.777	0.993
African	Null vs present	2.01 (1.23–3.30)	75.1	0.124	0.979	0.990
Adults	Null vs present	1.31 (1.15–1.50)	63.2	0.975	0.087	0.816
Children	Null vs present	1.21 (1.06–1.39)	27.0	0.999	0.876	0.996
HC	Null vs present	1.21 (1.05–1.39)	69.4	0.999	0.876	0.996
Matching	Null vs present	1.26 (1.05–1.52)	73.5	0.966	0.942	0.997
Nonmatching	Null vs present	1.13 (1.02–1.27)	57.7	1.000	0.976	0.999
ALL	Null vs present	1.22 (1.01–1.46)	63.7	0.988	0.968	0.998
GSTT1						
Overall	Null vs present	1.46 (1.32–1.60)	62.5	0.710	<0.001	<0.001
Indian	Null vs present	1.74 (1.27–2.38)	71.9	0.177	0.749	0.934
Asian	Null vs present	1.30 (1.16–1.46)	24.2	0.992	0.009	0.367
Caucasian	Null vs present	1.37 (1.17–1.59)	65.0	0.884	0.037	0.619
African	Null vs present	2.08 (1.32–3.26)	66.5	0.720	0.999	0.971
Adults	Null vs present	1.55 (1.32–1.82)	69.6	0.344	<0.001	0.006
Children	Null vs present	1.24 (1.09–1.43)	37.2	0.996	0.754	0.991
Adults and Children	Null vs present	1.59 (1.27–1.99)	67.1	0.305	0.143	0.655
HC	Null vs present	1.45 (1.28–1.66)	63.7	0.688	<0.001	0.005
NBDC	Null vs present	1.46 (1.26–1.69)	62.7	0.641	0.001	0.024
Matching	Null vs present	1.80 (1.44–2.24)	63.7	0.051	0.003	0.008
Nonmatching	Null vs present	1.35 (1.22–1.49)	51.7	0.982	<0.001	<0.001
AML	Null vs present	1.41 (1.19–1.66)	67.7	0.771	0.046	0.622
ALL	Null vs present	1.33 (1.16–1.53)	53.0	0.954	0.065	0.758
CML	Null vs present	1.88 (1.47–2.41)	64.5	0.037	0.017	0.033
Sensitivity analysis						
Quality score ≥10						
Overall	Null vs present	1.52 (1.34–1.72)	66.9	0.417	<0.001	<0.001
Indian	Null vs present	1.53 (1.08–2.17)	69.6	0.456	0.974	0.996
Asian	Null vs present	1.15 (1.01–1.31)	21.5	1.000	0.973	0.999
Caucasian	Null vs present	1.64 (1.37–1.96)	64.5	0.163	<0.001	0.003
African	Null vs present	2.12 (1.26–3.58)	71.9	0.098	0.981	0.989
Adults	Null vs present	1.58 (1.33–1.89)	71.3	0.285	0.002	0.030
Adults and Children	Null vs present	1.45 (1.14–1.83)	61.5	0.612	0.741	0.978
HC	Null vs present	1.56 (1.31–1.86)	71.0	0.331	0.002	0.038
NBDC	Null vs present	1.45 (1.23–1.72)	56.7	0.651	0.030	0.475
Matching	Null vs present	1.73 (1.37–2.17)	73.6	0.109	0.019	0.093
Nonmatching	Null vs present	1.41 (1.23–1.62)	59.2	0.809	0.002	0.069
AML	Null vs present	1.35 (1.12–1.63)	68.3	0.863	0.676	0.981
ALL	Null vs present	1.49 (1.19–1.88)	64.2	0.522	0.597	0.956
CML	Null vs present	1.93 (1.44–2.59)	64.9	0.047	0.202	0.332
GSTP1						
Overall	Val/Val vs. lle/lle	1.77 (1.40–2.24)	59.8	0.084	0.023	0.089
	lle/Val vs. lle/lle	1.24 (1.08–1.43)	67.7	0.996	0.757	0.991
	Val/Val vs. lle/lle + lle/Val	1.59 (1.29–1.95)	50.9	0.288	0.028	0.273
	Val/Val+lle/Val vs. lle/lle	1.32 (1.15–1.53)	72.6	0.955	0.193	0.905
	Val vs lle	1.31 (1.16–1.47)	75.0	0.989	0.004	0.220
Indian	Val/Val vs. lle/lle	3.01 (1.60–5.66)	76.8	0.015	0.976	0/961
	lle/Val vs. lle/lle	1.28 (1.08–1.53)	30.3	0.959	0.874	0.994
	Val/Val vs. lle/lle +lle/Val	2.65 (1.47–4.79)	74.8	0.030	0.977	0.974
	Val/Val+lle/Val vs. lle/lle	1.45 (1.17–1.80)	57.2	0.621	0.549	0.957
	Val vs lle	1.47 (1.19–1.80)	72.1	0.578	0.250	0.869
Caucasian	Val/Val vs. lle/lle	1.49 (1.10–2.01)	40.2	0.517	0.946	0.994
	Val/Val vs. lle/lle +lle/Val	1.31 (1.04–1.65)	15.3	0.875	0.961	0.997
	Val/Val+lle/Val vs. lle/lle	1.32 (1.02–1.72)	75.6	0.828	0.980	0.998
	Val vs lle	1.28 (1.05–1.55)	74.0	0.948	0.924	0.996
Adults	Val/Val vs. lle/lle	1.39 (1.06–1.82)	34.1	0.710	0.959	0.996
	Val/Val vs.lle/lle + lle/Val	1.27 (1.01–1.61)	20.2	0.915	0.981	0.999
	Val vs lle	1.18 (1.02–1.37)	64.6	0.999	0.968	0.999
Children	Val/Val vs. lle/lle	1.68 (1.10–2.58)	39.6	0.302	0.983	0.996
	Val/Val vs.lle/lle + lle/Val	1.60 (1.11–2.32)	25.8	0.367	0.973	0.995
Adults and Children	Val/Val vs. lle/lle	3.25 (1.61–6.53)	76.8	0.015	0.984	0.974
	lle/Val vs. lle/lle	1.64 (1.16–2.31)	73.7	0.305	0.938	0.989
	Val/Val vs. lle/lle +lle/Val	2.65 (1.41–5.02)	72.9	0.040	0.986	0.986
	Val/Val+lle/Val vs. lle/lle	1.82 (1.29–2.57)	77.3	0.136	0.831	0.945
	Val vs lle	1.72 (1.29–2.30)	80.4	0.176	0.588	0.883
HC	Val/Val vs. lle/lle	2.38 (1.66–3.41)	61.2	0.006	0.278	0.118
	lle/Val vs. lle/lle	1.27 (1.05–1.54)	65.2	0.955	0.940	0.997
	Val/Val vs. lle/lle + lle/Val	2.12 (1.53–2.94)	55.3	0.019	0.259	0.239
	Val/Val+lle/Val vs. lle/lle	1.39 (1.15–1.69)	69.6	0.778	0.552	0.967
	Val vs lle	1.40 (1.19–1.63)	71.5	0.813	0.018	0.419
Nonmatching	Val/Val vs. lle/lle	1.37 (1.07–1.76)	23.1	0.761	0.948	0.996
	Val/Val vs.lle/lle + lle/Val	1.30 (1.03–1.64)	19.2	0.886	0.968	0.998
	Val vs lle	1.13 (1.03–1.24)	6.1	1.000	0.908	0.998
Nonmatching	Val/Val vs. lle/lle	2.13 (1.49–3.06)	69.0	0.029	0.598	0.628
	lle/Val vs. lle/lle	1.36 (1.08–1.71)	78.4	0.799	0.914	0.994
	Val/Val vs. lle/lle + lle/Val	1.86 (1.36–2.54)	60.6	0.088	0.518	0.760
	Val/Val+lle/Val vs. lle/lle	1.47 (1.17–1.86)	81.7	0.567	0.701	0.972
	Val vs lle	1.44 (1.19–1.74)	82.8	0.664	0.193	0.852
AML	Val/Val vs. lle/lle	1.57 (1.10–2.24)	66.4	0.401	0.970	0.995
	lle/Val vs. lle/lle	1.37 (1.02–1.84)	83.1	0.727	0.980	0.998
	Val/Val vs. lle/lle + lle/Val	1.37 (1.01–1.84)	55.1	0.727	0.980	0.998
	Val/Val+lle/Val vs. lle/lle	1.42 (1.06–1.89)	84.8	0.646	0.962	0.996
	Val vs lle	1.34 (1.07–1.68)	85.1	0.836	0.930	0.996
ALL	Val/Val vs. lle/lle	1.90 (1.28–2.81)	52.1	0.118	0.917	0.968
	Val/Val vs. lle/lle +lle/Val	1.77 (1.25–2.53)	44.0	0.182	0.905	0.974
	Val/Val+lle/Val vs. lle/lle	1.26 (1.03–1.53)	51.4	0.961	0.953	0.998
	Val vs lle	1.29 (1.08–1.53)	61.0	0.958	0.782	0.990
CML	Val/Val vs. lle/lle	1.29 (1.08–1.53)	67.3	0.958	0.782	0.990
	Val/Val vs.lle/lle + lle/Val	2.13 (1.08–4.24)	60.1	0.159	0.995	0.997
Sensitivity analysis						
HWE						
Overall	Val/Val vs. lle/lle	1.58 (1.27–1.95)	26.3	0.314	0.061	0.455
	lle/Val vs. lle/lle	1.18 (1.02–1.37)	59.0	0.999	0.968	0.999
	Val/Val vs. lle/lle +lle/Val	1.45 (1.21–1.74)	7.3	0.642	0.092	0.722
	Val/Val+lle/Val vs. lle/lle	1.25 (1.07–1.45)	64.1	0.992	0.764	0.991
	Val vs lle	1.24 (1.10–1.40)	64.4	0.999	0.339	0.959
Indian	Val/Val vs. lle/lle	1.83 (1.11–3.03)	56.3	0.220	0.988	0.996
	lle/Val vs. lle/lle	1.24 (1.01–1.51)	12.7	0.971	0.971	0.998
	Val/Val vs. lle/lle + lle/Val	1.67 (1.05–2.64)	51.3	0.323	0.989	0.997
	Val/Val+lle/Val vs. lle/lle	1.34 (1.06–1.69)	42.1	0.830	0.942	0.996
	Val vs lle	1.33 (1.06–1.66)	61.2	0.856	0.932	0.996
Caucasian	Val/Val vs. lle/lle	1.70 (1.23–2.34)	21.0	0.221	0.837	0.964
	Val/Val vs. lle/lle +lle/Val	1.50 (1.14–1.95)	0.0	0.500	0.831	0.982
	Val/Val+lle/Val vs. lle/lle	1.39 (1.01–1.90)	76.4	0.684	0.983	0.998
	Val vs lle	1.36 (1.08–1.70)	72.8	0.805	0.896	0.993
Adults	Val/Val vs. lle/lle	1.39 (1.07–1.81)	0.0	0.714	0.953	0.996
	Val/Val vs.lle/lle + lle/Val	1.31 (1.02–1.69)	0.0	0.851	0.978	0.998
	Val vs lle	1.22 (1.01–1.46)	67.7	0.988	0.968	0.998
Children	Val/Val vs. lle/lle	1.68 (1.10–2.58)	39.6	0.302	0.983	0.996
	Val/Val vs.lle/lle + lle/Val	1.60 (1.11–2.32)	25.8	0.367	0.973	0.995
Adults and Children	Val/Val+lle/Val vs. lle/lle	1.39 (1.01–1.92)	57.0	0.678	0.985	0.998
HC	Val/Val vs. lle/lle	1.86 (1.38–2.50)	34.2	0.077	0.336	0.591
	Val/Val vs. lle/lle +lle/Val	1.71 (1.33–2.21)	16.0	0.158	0.207	0.603
	Val/Val+lle/Val vs. lle/lle	1.31 (1.05–1.62)	68.9	0.894	0.934	0.996
	Val vs lle	1.31 (1.11–1.55)	68.1	0.943	0.637	0.981
Matching	Val/Val vs. lle/lle	1.51 (1.11–2.05)	0.0	0.483	0.945	0.993
	Val/Val vs. lle/lle + lle/Val	1.43 (1.07–1.93)	0.0	0.623	0.969	0.997
	Val/Val+lle/Val vs. lle/lle	1.17 (1.01–1.35)	0.0	1.000	0.969	0.999
	Val vs lle	1.18 (1.05–1.32)	0.0	1.000	0.792	0.994
Non matching	Val/Val vs. lle/lle	1.66 (1.21–2.27)	47.1	0.263	0.851	0.972
	Val/Val vs. lle/lle + lle/Val	1.50 (1.15–1.94)	28.8	0.500	0.800	0.979
	Val/Val+lle/Val vs. lle/lle	1.31 (1.03–1.66)	76.9	0.869	0.967	0.998
	Val vs lle	1.29 (1.07–1.55)	76.6	0.946	0.874	0.994
ALL	Val/Val vs. lle/lle	1.60 (1.15–2.22)	26.8	0.350	0.933	0.989
	Val/Val vs.lle/lle + lle/Val	1.53 (1.14–2.06)	17.8	0.448	0.919	0.990
	Val vs lle	1.21 (1.03–1.43)	52.6	0.994	0.962	0.998
Quality score≥12						
Overall	Val/Val vs. lle/lle	1.62 (1.25–2.11)	45.7	0.284	0.549	0.910
	Val/Val vs. lle/lle + lle/Val	1.49 (1.18–1.89)	35.6	0.522	0.660	0.964
	Val/Val+lle/Val vs. lle/lle	1.23 (1.05–1.44)	64.0	0.993	0.910	0.996
	Val vs lle	1.23 (1.09–1.40)	65.5	0.999	0.633	0.985
Caucasian	Val/Val vs. lle/lle	1.54 (1.09–2.17)	28.7	0.440	0.969	0.995
	Val/Val vs. lle/lle +lle/Val	1.28 (1.01–1.64)	0.0	0.895	0.983	0.999
	Val/Val+lle/Val vs. lle/lle	1.48 (1.04–2.10)	77.2	0.530	0.981	0.997
	Val vs lle	1.38 (1.08–1.77)	73.1	0.744	0.938	0.995
Indian	Val/Val vs. lle/lle	2.15 (1.22–3.76)	62.6	0.103	0.986	0.992
	Val/Val vs. lle/lle + lle/Val	1.97 (1.16–3.35)	60.3	0.157	0.987	0.995
	Val/Val+lle/Val vs. lle/lle	1.29 (1.03–1.63)	47.1	0.897	0.973	0.998
	Val vs lle	1.32 (1.07–1.64)	61.6	0.876	0.933	0.996
Adults	Val/Val vs. lle/lle	1.42 (1.07–1.88)	33.1	0.649	0.957	0.996
	Val/Val vs. lle/lle + lle/Val	1.29 (1.02–1.64)	17.3	0.891	0.977	0.998
	Val/Val+lle/Val vs. lle/lle	1.24 (1.01–1.51)	68.9	0.971	0.971	0.998
	Val vs lle	1.21 (1.04–1.41)	65.5	0.997	0.936	0.997
HC	Val/Val vs. lle/lle	1.94 (1.37–2.76)	44.2	0.076	0.750	0.874
	Val/Val vs. lle/lle +lle/Val	1.77 (1.29–2.44)	36.9	0.156	0.758	0.930
	Val/Val+lle/Val vs. lle/lle	1.30 (1.05–1.62)	64.8	0.899	0.956	0.997
	Val vs lle	1.31 (1.10–1.55)	64.0	0.943	0.637	0.981
Matching	Val/Val vs. lle/lle	1.45 (1.11–1.90)	16.9	0.597	0.922	0.993
	Val/Val vs.lle/lle + lle/Val	1.41 (1.08–1.83)	18.9	0.679	0.935	0.995
	Val vs lle	1.14 (1.04–1.25)	0.0	1.000	0.841	0.996
Non matching	Val/Val vs. lle/lle	1.81 (1.07–3.06)	67.1	0.242	0.991	0.997
	Val/Val vs.lle/lle + lle/Val	1.58 (1.02–2.46)	55.9	0.409	0.991	0.998
CML	Val/Val vs. lle/lle	3.17 (1.89–5.32)	16.7	0.002	0.845	0.489
	Val/Val vs.lle/lle + lle/Val	2.80 (1.79–4.39)	0.0	0.003	0.688	0.322
	Val vs lle	1.41 (1.05–1.89)	68.3	0.661	0.970	0.997
HWE and Quality score≥12						
Overall	Val/Val vs. lle/lle	1.63 (1.24–2.13)	35.2	0.271	0.559	0.909
	Val/Val vs. lle/lle +lle/Val	1.49 (1.18–1.88)	20.2	0.522	0.597	0.956
	Val/Val+lle/Val vs. lle/lle	1.27 (1.06–1.53)	66.8	0.960	0.925	0.996
	Val vs lle	1.26 (1.08–1.46)	67.7	0.990	0.680	0.986
Indian	Val/Val vs. lle/lle	1.91 (1.07–3.40)	62.6	0.206	0.993	0.997
	Val/Val vs. lle/lle + lle/Val	1.74 (1.03–2.96)	57.8	0.292	0.993	0.998
	Val/Val+lle/Val vs. lle/lle	1.34 (1.02–1.76)	51.7	0.791	0.978	0.998
	Val vs lle	1.34 (1.04–1.74)	67.4	0.801	0.972	0.998
Caucasian	Val/Val vs. lle/lle	1.87 (1.28–2.74)	0.0	0.129	0.911	0.968
	lle/Val vs. lle/lle	1.55 (1.02–2.34)	71.9	0.438	0.988	0.998
	Val/Val vs. lle/lle +lle/Val	1.59 (1.11–2.30)	0.0	0.379	0.973	0.995
	Val/Val+lle/Val vs. lle/lle	1.63 (1.12–2.37)	68.7	0.332	0.969	0.994
	Val vs lle	1.50 (1.17–1.91)	56.4	0.500	0.668	0.964
Adults	Val/Val vs. lle/lle	1.38 (1.05–1.82)	3.2	0.723	0.969	0.997
	Val vs lle	1.24 (1.02–1.52)	70.4	0.967	0.975	0.999
HC	Val/Val vs. lle/lle	1.83 (1.29–2.58)	40.3	0.128	0.815	0.937
	Val/Val vs. lle/lle + lle/Val	1.66 (1.23–2.25)	28.2	0.257	0.809	0.963
	Val/Val+lle/Val vs. lle/lle	1.33 (1.05–1.68)	66.7	0.844	0.952	0.997
	Val vs lle	1.32 (1.09–1.59)	66.8	0.911	0.791	0.989
Matching	Val/Val vs. lle/lle	1.51 (1.11–2.05)	0.0	0.483	0.945	0.993
	Val/Val vs. lle/lle + lle/Val	1.43 (1.07–1.93)	0.0	0.623	0.969	0.997
	Val/Val+lle/Val vs. lle/lle	1.17 (1.01–1.35)	0.0	1.000	0.969	0.999
	Val vs lle	1.18 (1.05–1.32)	0.0	1.000	0.792	0.994
Nonmatching	Val/Val vs. lle/lle	1.81 (1.07–3.06)	67.1	0.242	0.991	0.997
	Val/Val vs. lle/lle +lle/Val	1.58 (1.02–2.46)	55.9	0.409	0.991	0.998
The combined effects of GSTM1 and GSTT1 polymorphisms						
Overall	Model 1	1.66 (1.37–2.00)	30.3	0.143	0.001	0.006
	Model 3	2.44 (1.86–3.21)	51.2	<0.001	0.001	<0.001
	Model 4	1.29 (1.11–1.50)	52.2	0.975	0.489	0.971
	Model 5	1.44 (1.25–1.66)	51.5	0.713	0.001	0.030
	Model 6	2.16 (1.65–2.81)	55.4	0.003	0.003	0.001
Indian	Model 1	1.92 (1.18–3.12)	52.9	0.159	0.981	0.993
	Model 3	3.16 (1.90–5.25)	0.0	0.002	0.816	0.412
	Model 6	2.83 (1.73–4.64)	0.0	0.006	0.863	0.674
Asian	Model 1	1.43 (1.04–1.97)	22.1	0.615	0.979	0.997
	Model 3	2.47 (1.55–3.95)	57.5	0.019	0.896	0.860
	Model 4	1.35 (1.02–1.80)	45.3	0.764	0.982	0.998
	Model 5	1.57 (1.20–2.05)	44.0	0.369	0.713	0.959
	Model 6	2.05 (1.40–3.00)	50.3	0.054	0.803	0.873
Caucasian	Model 1	1.65 (1.14–2.39)	40.6	0.307	0.963	0.993
	Model 3	1.98 (1.16–3.37)	61.5	0.153	0.987	0.994
	Model 4	1.30 (1.05–1.60)	41.3	0.912	0.936	0.996
	Model 5	1.37 (1.17–1.61)	13.7	0.864	0.132	0.843
Adults	Model 1	1.44 (1.18–1.76)	0.0	0.655	0.360	0.923
	Model 2	1.27 (1.04–1.54)	50.7	0.955	0.940	0.997
	Model 3	2.51 (1.71–3.68)	60.0	0.004	0.367	0.131
	Model 4	1.34 (1.15–1.57)	35.3	0.919	0.242	0.919
	Model 5	1.50 (1.29–1.74)	33.0	0.500	< 0.001	0.006
	Model 6	2.26 (1.53–3.33)	65.8	0.019	0.662	0.610
Adults and children	Model 6	1.94 (1.04–4.36)	43.6	0.267	0.998	0.999
HC	Model 1	1.73 (1.31–2.30)	39.2	0.163	0.498	0.835
	Model 3	2.59 (1.71–3.93)	51.1	0.005	0.600	0.310
	Model 5	1.45 (1.16–1.80)	60.3	0.621	0.549	0.957
	Model 6	2.33 (1.54–3.58)	59.7	0.022	0.8361	0.811
NBDC	Model 1	1.60 (1.22–2.10)	25.7	0.321	0.687	0.949
	Model 2	1.29 (1.11–1.50)	0.0	0.975	0.489	0.971
	Model 3	2.31 (1.56–3.43)	55.7	0.016	0.672	0.589
	Model 4	1.36 (1.18–1.57)	1.6	0.909	0.029	0.572
	Model 5	1.49 (1.25–1.78)	33.9	0.529	0.020	0.338
	Model 6	1.86 (1.33–2.61)	46.7	0.107	0.756	0.904
Matching	Model 1	1.60 (1.29–1.99)	0.0	0.281	0.079	0.491
	Model 3	2.57 (1.61–4.12)	64.2	0.463	0.999	0.999
	Model 4	1.31 (1.09–1.58)	41.0	0.922	0.837	0.992
	Model 5	1.46 (1.24–1.73)	33.8	0.623	0.019	0.367
	Model 6	2.33 (1.44–3.76)	69.6	0.036	0.937	0.942
Nonmatching	Model 1	1.67 (1.24–2.27)	48.0	0.247	0.811	0.963
	Model 3	2.38 (1.70–3.33)	37.6	0.004	0.106	0.027
	Model 5	1.43 (1.13–1.80)	62.1	0.658	0.779	0.983
	Model 6	2.07 (1.52–2.81)	36.5	0.019	0.137	0.133
AML	Model 3	2.15 (1.35–3.43)	55.1	0.065	0.953	0.970
	Model 5	1.41 (1.09–1.82)	46.7	0.683	0.924	0.994
	Model 6	1.85 (1.22–2.80)	51.1	0.161	0.957	0.986
ALL	Model 1	2.15 (1.43–3.23)	39.9	0.041	0.846	0.880
	Model 3	2.79 (1.47–5.30)	52.0	0.029	0.983	0.981
	Model 4	1.52 (1.13–2.05)	44.5	0.465	0.929	0.991
	Model 5	1.66 (1.25–2.20)	42.7	0.240	0.636	0.922
	Model 6	2.23 (1.20–4.14)	55.4	0.105	0.991	0.994
CML	Model 1	1.54 (1.18–2.01)	7.2	0.423	0.778	0.973
	Model 3	2.58 (1.57–4.24)	51.4	0.016	0.919	0.880
	Model 5	1.37 (1.06–1.77)	62.5	0.756	0.955	0.996
	Model 6	2.41 (1.45–4.00)	55.8	0.033	0.952	0.953
Sensitivity analysis						
Quality score≥10						
Overall	Model 1	1.56 (1.28–1.91)	26.3	0.352	0.045	0.413
	Model 3	2.41 (1.76–3.29)	55.3	0.001	0.021	0.002
	Model 4	1.27 (1.09–1.48)	48.3	0.983	0.691	0.986
	Model 5	1.42 (1.22–1.65)	51.7	0.763	0.006	0.201
	Model 6	2.17 (1.61–2.94)	57.8	0.009	0.063	0.033
Indian	Model 1	1.92 (1.18–3.12)	52.9	0.159	0.981	0.993
	Model 3	3.16 (1.90–5.25)	0.0	0.002	0.816	0.412
	Model 6	2.83 (1.73–4.64)	0.0	0.006	0.863	0.674
Caucasian	Model 4	1.28 (1.04–1.57)	33.6	0.936	0.950	0.997
	Model 5	1.34 (1.13–1.58)	11.5	0.910	0.354	0.947
Adults	Model 1	1.43 (1.16–1.76)	0.0	0.674	0.521	0.956
	Model 2	1.31 (1.08–1.60)	48.8	0.908	0.899	0.995
	Model 3	2.40 (1.61–3.58)	59.9	0.011	0.626	0.463
	Model 4	1.37 (1.16–1.61)	35.8	0.864	0.132	0.843
	Model 5	1.51 (1.29–1.77)	37.4	0.467	0.001	0.022
	Model 6	2.13 (1.43–3.18)	64.7	0.043	0.834	0.875
Adults and children	Model 6	1.94 (1.04–4.36)	43.6	0.267	0.998	0.999
HC	Model 1	1.63 (1.21–2.18)	34.9	0.288	0.774	0.961
	Model 3	2.56 (1.60–4.10)	55.0	0.013	0.875	0.798
	Model 5	1.41 (1.11–1.80)	63.6	0.690	0.894	0.992
	Model 6	2.39 (1.50–3.80)	58.7	0.024	0.904	0.890
NBDC	Model 1	1.47 (1.12–1.94)	15.7	0.557	0.921	0.992
	Model 2	1.24 (1.06–1.45)	0.0	0.991	0.876	0.995
	Model 3	2.17 (1.43–3.29)	54.9	0.041	0.865	0.893
	Model 4	1.30 (1.12–1.50)	0.0	0.975	0.251	0.931
	Model 5	1.41 (1.19–1.66)	17.2	0.771	0.046	0.622
	Model 6	1.85 (1.27–2.69)	51.3	0.136	0.904	0.967
Matching	Model 1	1.60 (1.29–1.99)	0.0	0.281	0.079	0.491
	Model 3	2.57 (1.61–4.12)	64.2	0.463	0.999	0.999
	Model 4	1.31 (1.09–1.58)	41.0	0.922	0.837	0.992
	Model 5	1.46 (1.24–1.73)	33.8	0.623	0.019	0.367
	Model 6	2.33 (1.44–3.76)	69.6	0.036	0.937	0.942
Nonmatching	Model 1	1.51 (1.04–2.19)	48.0	0.486	0.984	0.997
	Model 3	2.26 (1.45–3.53)	46.7	0.036	0.904	0.915
	Model 5	1.37 (1.03–1.82)	65.1	0.734	0.976	0.998
	Model 6	2.04 (1.40–2.98)	37.7	0.056	0.802	0.876
ALL	Model 1	1.92 (1.28–2.86)	29.6	0.112	0.922	0.969
	Model 3	3.10 (1.48–6.49)	58.1	0.027	0.990	0.988
	Model 4	1.43 (1.07–1.91)	39.3	0.627	0.961	0.996
	Model 5	1.59 (1.18–2.14)	45.8	0.350	0.863	0.980
	Model 6	2.66 (1.38–5.15)	54.1	0.045	0.988	0.989
CML	Model 1	1.55 (1.15–2.09)	16.4	0.415	0.907	0.988
	Model 3	2.39 (1.37–4.16)	54.3	0.050	0.976	0.981
	Model 5	1.38 (1.03–1.83)	66.2	0.719	0.972	0.997
	Model 6	2.21 (1.26–3.87)	27.7	0.088	0.984	0.990
The combined effects of GSTM1 and GSTP1 polymorphisms						
Overall	Model 4	1.95 (1.35–2.80)	21.5	0.078	0.793	0.897
	Model 6	1.95 (1.37–2.77)	30.4	0.071	0.729	0.857
Indian	Model 4	1.72 (1.10–2.70)	33.5	0.276	0.985	0.996
	Model 6	1.65 (1.14–2.40)	19.6	0.309	0.966	0.993
HC	Model 4	1.82 (1.21–2.74)	25.9	0.177	0.959	0.987
	Model 6	1.88 (1.23–2.89)	41.8	0.152	0.964	0.987
Matching	Model 6	2.20 (1.25–3.89)	37.1	0.094	0.986	0.992
Non-matching	Model 4	2.07 (1.34–3.20)	0.0	0.074	0.935	0.964
	Model 5	1.44 (1.08–1.92)	0.0	0.610	0.955	0.995
	Model 6	1.76 (1.05–2.96)	42.1	0.273	0.992	0.997
ALL	Model 4	1.86 (1.01–3.43)	51.9	0.245	0.995	0.998
	Model 6	1.92 (1.30–2.83)	0.0	0.106	0.902	0.960
CML	Model 4	2.08 (1.27–3.40)	1.4	0.096	0.973	0.986
Sensitivity analysis						
HWE and Quality score≥10						
Overall	Model 4	1.95 (1.35–2.80)	21.5	0.078	0.793	0.897
	Model 6	1.95 (1.37–2.77)	30.4	0.071	0.729	0.857
The combined effects of GSTT1 and GSTP1 polymorphisms						
Overall	Model 3	1.50 (1.04–2.15)	59.8	0.500	0.982	0.997
	Model 4	4.24 (2.49–7.24)	0.0	0.000	0.632	0.027
	Model 5	1.70 (1.30–2.22)	32.2	0.179	0.352	0.765
	Model 6	3.31 (1.85–5.91)	14.8	0.004	0.933	0.780
Indian	Model 3	1.65 (1.05–2.59)	61.9	0.339	0.989	0.997
	Model 4	4.39 (2.51–7.68)	0.0	0.000	0.721	0.049
	Model 5	1.91 (1.45–2.50)	0.8	0.039	0.059	0.106
	Model 6	3.39 (1.94–5.94)	7.8	0.002	0.901	0.617
HC	Model 3	1.50 (1.04–2.15)	59.8	0.500	0.982	0.997
	Model 4	4.24 (2.49–7.24)	0.0	0.000	0.632	0.027
	Model 5	1.70 (1.30–2.22)	32.2	0.179	0.352	0.765
	Model 6	3.31 (1.85–5.91)	14.8	0.004	0.933	0.780
Matching	Model 4	4.61 (1.64–12.97)	16.8	0.017	0.996	0.994
	Model 5	1.40 (1.04–1.89)	0.0	0.674	0.976	0.998
CML	Model 2	1.91 (1.35–2.68)	0.0	0.081	0.690	0.849
	Model 4	3.29 (1.37–7.89)	1.9	0.039	0.995	0.995
	Model 5	1.61 (1.05–2.47)	50.4	0.373	0.987	0.997
	Model 6	2.40 (1.21–14.26)	31.8	0.303	0.999	0.999
Sensitivity analysis						
HWE and Quality score≥10						
Overall	Model 3	1.50 (1.04–2.15)	59.8	0.500	0.982	0.997
	Model 4	4.24 (2.49–7.24)	0.0	0.000	0.632	0.027
	Model 5	1.70 (1.30–2.22)	32.2	0.179	0.352	0.765
	Model 6	3.31 (1.85–5.91)	14.8	0.004	0.933	0.780

## Discussion

Leukemia is characterized by abnormal hematopoietic function and malignant cloning of white blood cells (Ouerhani et al., 2011). Gene polymorphisms play a significant role in the development of leukemia, and GST null has been studied by many scholars. Studies demonstrated that complete deletion of *GSTM1*, *GSTT1,* or *GSTP1* polymorphisms brought about diminished gene expression and enzymatic activity (Strange et al., 1998; Strange et al., 2001; Hollman et al., 2016). Thus, it is significant to study the connection between GST polymorphisms and leukemia risk. Many studies have analyzed the roles of M1, T1, and P1 polymorphisms in leukemia risk. Regrettably, no reliable testimony has been obtained to show whether there is an association between them. This may be due to heterogeneities such as ethnicity, small sample size, matching, type of leukemia, etc. Therefore, an updated meta-analysis was generated to explore these issues. At this point, totally 91 articles were finally selected to provide proof for the association between GST polymorphisms and leukemia risk.

Overall, the present study showed that the *GSTM1*, *GSTT1*, and *GSTP1* polymorphisms significantly added the risk of leukemia in the overall and several subgroups. Moreover, with the combined *GSTM1* and *GSTT1*, *GSTM1* and *GSTP1*, and *GSTT1* and *GSTP1* polymorphisms, there were six gene models to explore the association with leukemia risk, and positive results were observed in partial gene models. However, there was no significant contact between the composite effects of these three polymorphisms with leukemia in overall analysis. Furthermore, in sensitivity analysis, when selecting Hardy–Weinberg equilibrium (HWE) and medium and high-quality studies, we had come to a similar conclusion. Finally, in view of the quantities of genomic data being produced currently, we used a more exact Bayesian measure of false-positive found in genetic epidemiological studies in the present study. Using FPRP and BFDP to correct the positive results, in all of these positive results we found previously, only the association between GSTT1 null and leukemia risk was watched in ethnicity (BFDP = 0.367, FPRP = 0.009). Our results indicated that the false-positive associations were common between SNP and disease risk. Moreover, these results further confirmed that the occurrence of leukemia was the result of multiple genes.

Thirteen previous meta-analyses analyzed the links between *GSTM1*, *GSTT1*, and *GSTP1* polymorphisms and the risk of leukemia. Tang et al. (2014), Ye and Song (2005), Wang et al. (2019), Zhang et al. (2017), Das et al. (2009), and He et al. (2014) discussed the association between *GSTM1* and *GSTT1* null genotypes and the risk of leukemia, and their results suggested that there was a significant association between *GSTM1* and *GSTT1* polymorphisms and leukemia risk. The studies of Ma et al. (2014) and Tang et al. (2013) showed that *GSTM1* null genotypes increased the risk of acute leukemia. The results of Moulik et al. (2014) demonstrated that there was a significant connection between *GSTP1* polymorphism with the risk of leukemia; however, Huang et al. (2013) discussed the association between *GSTP1* polymorphism and the risk of leukemia, and the results showed that there was no significant connection. The number of studies and sample sizes in the current study were larger than the published meta-analyses. When comparing to the present meta-analysis, previous studies had several defects. First, none of the previous studies performed quality assessments. Second, HWE was not reported in any published meta-analysis. Third, all previous meta-analyses did not adjust the positive results for multiple comparisons, and only five previous meta-analyses (Ye and Song, 2005; Huang et al., 2013; Tang et al., 2013; Tang et al., 2014; Zhang et al., 2017) conducted subgroup analysis. Fourth, there were no published meta-analyses that performed sensitivity analysis. Moreover, previous meta-analyses had a small sample size; most eligible studies were not assessed for quality assessment; and the reliability of positive results was not evaluated using FPRP, BFDP, and Venice criteria. In addition, they failed to establish a more complete genetic model. Thus, their meta-analyses might have lower credibility.

The current meta-analysis had some advantages over previously published meta-analyses. 1) We explored the credibility by applying the Venice criteria, FPRP, and BFDP. 2) The qualified studies were evaluated for quality. 3) The sample size was larger and the data collected were more detailed over the previous meta-analyses. 4) We conducted more subunit analyses, such as ethnicity, age group, type of control, matching or not, type of leukemia, quality score, and HWE. 5) We established a more complete genetic model. 6) Our study is the first one to explore the combined effects of *GSTM1*, *GSTT1,* and *GSTP1* polymorphisms with leukemia risk. Nonetheless, there are still some potential limitations for this current study. First, in this study, we only studied published research studies, and as we all know, the positive results are more likely to be published than the negative ones. Second, the mechanism of leading to leukemia is greatly sophisticated, and thus a single-gene mutation is not likely to generate remarkably to its development. Third, no consideration was given to if the genotype distribution of *GSTM1* and *GSTT1* polymorphisms in control group was in HWE because we could not calculate the HWE on these two genes. Fourth, the heterogeneity of *GSTM1*, *GSTT1,* and *GSTP1* was large; therefore, the random-effect model was selected, and after subgroup and sensitivity analysis, no source of heterogeneity was found. Hence, the current meta-analysis with a large sample size and enough subgroups will be conducive to confirm our discoveries.

This meta-analysis strongly suggests that only a minority of meaningful associations are credible results. Hence, larger-scale investigations of this topic should be performed in the future to verify or rebut our findings.

## Data Availability

The original contributions presented in the study are included in the article/Supplementary Materials, further inquiries can be directed to the corresponding authors.

## Abbreviations

GSTs, glutathione S-transferases; GSTM1, glutathione S-transferase M1; GSTT1, glutathione S-transferase T1; GSTP1, glutathione S-transferase P1; AML, acute myeloid leukemia; ALL, acute lymphoblastic leukemia; CML, chronic myeloid leukemia; CLL, chronic lymphoblastic leukemia; HC, healthy controls; NBDC, Nonblood disease controls; BFDP, Bayesian false discovery probability; FPRP, false-positive report probability; HWE, Hardy–Weinberg equilibrium; ORs, odds ratios; CIs, confidence intervals.
