# Supplementary material for: Individual and combined effects of the GSTM1, GSTT1, and GSTP1 polymorphisms on leukemia risk: An updated meta-analysis
Source: Front Genet. 2022 Oct 31;13:976673. doi: 10.3389/fgene.2022.976673 (PMC9659912; doi:10.3389/fgene.2022.976673)
Supplement: Supplementary file 1 [file Table1.DOCX]

**Supplemental references**

1. Basu T, Gale RE, Langabeer S, et al. Glutathione S-transferase theta 1 (GSTT1) gene defect in myelodysplasia and acute myeloid leukaemia. Lancet 1997, 349, 1450
2. Chen CL, Liu Q, Pui CH, Rivera GK, Sandlund JT et al. (1997) Higher frequency of glutathione S-transferase deletions in black children with acute lymphoblastic leukemia. Blood 89: 1701-1707. PubMed: 9057653.
3. Krajinovic M, Labuda D, Richer C, Karimi S, Sinnett D (1999) Susceptibility to childhood acute lymphoblastic leukemia: influence of CYP1A1, CYP2D6, GSTM1, and GSTT1 genetic polymorphisms. Blood 93: 1496-1501. PubMed: 10029576
4. Sasai Y, Horike S, Misawa S, et al. Genotype of glutathione s-transferase and other genetic configurations in myelodysplasia. Leukemia Res 1999, 23, 975–981.
5. Lemos MC, Cabrita FJ, Silva HA, et al. Genetic polymorphism of CYP2D6, GSTM1 and NAT2 and susceptibility to haemato-logical neoplasias. Carcinogenesis 1999, 20, 1225–1229.
6. Saadat I, Saadat M (2000) The glutathione S-transferase mu polymorphism and susceptibility to acute lymphocytic leukemia. Cancer Lett 158: 43-45. doi:10.1016/S0304-3835(00)00504-8. PubMed: 10940507.
7. Woo MH, Shuster JJ, Chen C, Bash RO, Behm FG et al. (2000) Glutathione S-transferase genotypes in children who develop treatment-related acute myeloid malignancies. Leukemia 14: 232-237. doi:10.1038/sj.leu.2401660. PubMed: 10673738
8. Crump C, Chen C, Appelbaum FR, et al. Glutathione s-transferase thea 1 gene deletion and risk of acute myeloid leukaemia. Cancer Epidemiol Biomarkers Prev 2000, 9, 457–460.
9. Rollinson S, Roddam P, Kane E, et al. Polymorphic variation within the glutathione S-transferase genes and risk of adult acute leukaemia. Carcinogenesis 2000, 21, 43–47.
10. Naoe T, Takeyama K, Yokozawa T, et al. Analysis of genetic polymorphism in NQO1, GST-M1, GST-T1, and CYP3A4 in 469 Japanese patients with therapy-related leukemia/ myelodysplastic syndrome and de novo acute myeloid leukemia. Clin Cancer Res 2000;6:4091–4095.
11. Lo ¨ffler H, Bergmann J, Hochhaus A, et al. (2001) Reduced risk for chronic myelogenous leukemia in individuals with the cyto- chrome P-450 gene polymorphism CYP1A1*2A. Blood 98:3874–3875
12. Arruda VR, Lima CSP, Grignoli CRE,et al. Increased risk for acute myeloid leukemia individuals with glutathiones-transferase mu 1 (GSTM1) and theta 1 (GSTT1) gene defects. Eur J Haematol 2001,66, 383–388.
13. Allan JM, Wild CP, Rollinson S, et al. Polymorphism in glutathione S-transferase P1 is associated with susceptibility to chemotherapy-induced leukemia. Proc Natl Acad Sci USA 2001;98:11592–11597.
14. Krajinovic M, Labuda D and Sinnett D. Glutathione S-transferase P1 genetic polymorphisms and susceptibility to childhood acute lymphoblastic leukaemia. Pharmacogenetics 12: 655-658.
15. Haase D, Binder C, Bunger J, et al. Increased risk for therapy-associated hematologic malignancies in patients with carcinoma of the breast and combined homozygous gene deletions of glutathi-one transferase M1 and T1. Leukemia Res 2002, 26, 249–254.
16. Alves S, Amorim A, Ferreira F, Norton L, Prata MJ (2002) The GSTM1 and GSTT1 genetic polymorphisms and susceptibility to acute lymphoblastic leukemia in children from north Portugal. Leukemia 16:1565-1567. doi:10.1038/sj.leu.2402543. PubMed: 12145701.
17. Davies SM, Bhatia S, Ross JA, Kiffmeyer WR, Gaynon PS, Radloff GA, Robison LL, Perentesis JP. Glutathione S-transferase genotypes, genetic susceptibility, and outcome of therapy in childhood acute lymphoblastic leukemia. Blood. 2002 Jul 1;100(1):67-71. doi: 10.1182/blood.v100.1.67. PMID: 12070010.
18. Yuille M, Condie A, Hudson C, Kote-Jarai Z, Stone E, Eeles R, Matutes E, Catovsky D, Houlston R. Relationship between glutathione S-transferase M1, T1, and P1 polymorphisms and chronic lymphocytic leukemia. Blood. 2002 Jun 1;99(11):4216-8. doi: 10.1182/blood.v99.11.4216. PMID: 12010828.
19. Balta G, Yuksek N, Ozyurek E, Ertem U, Hicsonmez G et al. (2003) Characterization of MTHFR, GSTM1, GSTT1, GSTP1, and CYP1A1 genotypes in childhood acute leukemia. Am J Hematol 73: 154-160. doi:10.1002/ajh.10339. PubMed: 12827651.
20. Zhang L. Correlation between S- transferase M1 and T1 gene polymorphism and childhood leukemia [D]. Zhengzhou University, 2003.
21. Yuan, X. J. , Gu, L. J. , Xue, H. L. , Tang, J. Y. , & Song, D. L. . (2003). [analysis on gst-pi genetic polymorphism in children with acute leukemia]. National Medical Journal of China, 83(21), 1863-1866.
22. Wang J, Zhang L, Feng JF, et la (2004). Genetic polymorphisms analysis of Glutathione s-transferase m1 and ti in children with acute lymphoblastic leukemia. JHuazhong University of Science and Technology [Med Sci], 24, 243-4 (in Chinese)
23. Zou LL, Lin GF, Ma QW, Zhang DS, Shen JH (2004). Glutathione S-transferase T1, M1 genetic polymorphisms in leukemia patients in Shanghai area. Chin J Oncol, 11, 649-51 (in Chinese)
24. Canalle R, Burim RV, Tone LG, Takahashi CS (2004) Genetic polymorphisms and susceptibility to childhood acute lymphoblastic leukemia. Environ Mol Mutagen 43: 100-109. doi:10.1002/em.20003.PubMed: 14991750
25. Seedhouse C, Faulkner R, Ashraf N, et al. Polymorphisms in genes involved in homologous recombination repair interacts to increase the risk of developing acute myeloid leukaemia. Clinical Cancer Res 2004, 10, 2675–2680.
26. Joseph T, Kusumakumary P, Chacko P, Abraham A, Radhakrishna Pillai M (2004) Genetic polymorphism of CYP1A1, CYP2D6, GSTM1 and GSTT1 and susceptibility to acute lymphoblastic leukaemia in Indian children. Pediatr Blood Cancer 43: 560-567. doi:10.1002/pbc. 20074. PubMed: 15382273.
27. D’Alo F, Voso MT, Guidi F, et al. Polymorphisms of CYP1A1 and glutathione S-transferase and susceptibility to adult acute myeloid leukemia. Haematologica 2004;89:664–670..
28. Liu QX, Chen HC, Liu XF, et al. (2005). Study on the relationship between polymorphisms of CYP1A1, GSTM1, GSTT1 genes and the susceptibility to acute leukemia in the general population of Hunan province. Chin J Epidemiol, 12, 975-79 (in Chinese).
29. Mondal BC, Paria N, Majumdar S, et al. (2005) Glutathione S-transferase M1 and T1 null genotype frequency in chronic myeloid leukaemia. Eur J Cancer Prev 14:281–284.
30. Clavel J, Bellec S, Rebouissou S, Ménégaux F, Feunteun J et al. (2005) Childhood leukaemia, polymorphisms of metabolism enzyme genes, and interactions with maternal tobacco, coffee and alcohol consumption during pregnancy. Eur J Cancer Prev 14: 531-540. doi: 10.1097/00008469-200512000-00007. PubMed: 16284498
31. Pakakasama S, Mukda E, Sasanakul W, Kadegasem P, Udomsubpayakul U et al. (2005) Polymorphisms of drug-metabolizing enzymes and risk of childhood acute lymphoblastic leukemia. Am J Hematol 79: 202-205. doi:10.1002/ajh.20404. PubMed: 15981231
32. Hishida A, Terakura S, Emi N, et al. (2005) GSTT1 and GSTM1 deletions, NQO1 C609T polymorphism and risk of chronic myelogenous leukemia in Japanese. Asian Pac J Cancer Prev 6:251–255
33. Yang L, Zhang Y, Zhang MR, et al. [Relationship between GSTT1, GSTM1 and NQO1 gene polymorphism and acute myeloid leukemia and recurrent chromosome translocations].Zhonghua Yi Xue Za Zhi 2005;85:2312–2316.
34. Aydin-Sayitoglu M, Hatirnaz O, Erensoy N, Ozbek U (2006) Role of CYP2D6, CYP1A1, CYP2E1, GSTT1, and GSTM1 genes in the susceptibility to acute leukemias. Am J Hematol 81: 162-170. doi: 10.1002/ajh.20434. PubMed: 16493615
35. Bajpai P, Tripathi AK, Agrawal D (2007) Increased frequencies of glutathione-S-transferase (GSTM1 and GSTT1) null genotypes in Indian patients with chronic myeloid leukemia. Leuk Res 31:1359–1363.
36. Pigullo S, Haupt R, Dufour C, Di Michele P, Valsecchi MG et al. (2007) Are genotypes of glutathione S-transferase superfamily a risk factor for childhood acute lymphoblastic leukemia? Results of an Italian case-control study. Leukemia 21: 1122-1124. PubMed: 17315021
37. Bolufer P, Collado M, Barragan E, et al. The po-tential effect of gender in combination with common genetic polymorphisms of drug-metabolizing en-zymes on the risk of developing acute leukemia. Haematologica. 2007;92(3):308-314.
38. Voso MT, Fabiani E, D'Alo' F, Guidi F, Di Ruscio A, Sica S, Pagano L, Greco M, Hohaus S, Leone G. Increased risk of acute myeloid leukaemia due to polymorphisms in detoxification and DNA repair enzymes. Ann Oncol. 2007 Sep;18(9):1523-8. doi: 10.1093/annonc/mdm191. PMID: 17761709
39. Eyada TK, El Ghonemy EG, El Ghoroury EA, et al. Study of genetic polymorphism of xenobiotic enzymes in acute leuke-mia. Blood Coagul Fibrinolysis 2007;18:489–495.
40. Gatedee J, Pakakassama S, Muangman S, Pongstaporn W. Glutathione S-transferase P1 genotypes, genetic susceptibility and outcome of therapy in thai childhood acute lymphoblastic leukemia. Asian Pac J Cancer Prev. 2007;8(2):294-296.
41. Bhatla D, Gerbing RB, Alonzo TA, et al. DNA repair polymorphisms and outcome of chemotherapy for acute myelogenous leukemia: a report from the Children’s Oncology Group. Leukemia 2008;22:265– 72.
42. Majumdar S, Mondal BC, Ghosh M, et al. Asso-ciation of cytochrome P450, glutathione S-transferase and N-acetyl transferase 2 gene polymor- phisms with incidence of acute myeloid leukemia. Eur J Cancer Prev. 2008;17(2):125-132.
43. Muller P, Asher N, Heled M, et al. Polymorphisms in transporter and phase II metabolism genes as potential modifiers of the predisposition to and treatment outcome of de novo acute myeloid leukemia in Israeli ethnic groups. Leuk Res 2008;32:919–929.
44. Jiang LJ, Chen M, Tan GF (2008). The association between polymorphism of cytochrome P4501A1 and glutathione S-transferase M1, T1 genes and acute lymphoblastic leukemia. JYoujiang Medical College for Nationalities, 5, 721-3 (in Chinese)
45. Suneetha KJ, Nancy KN, Rajalekshmy KR, Sagar TG, Rajkumar T (2008). Role of GSTM1 (Present/Null) and GSTP1 (Ile105V al) polymorphisms in susceptibility to acute lymphoblastic leukemia among the South Indian population. Asian Pac J Cancer Prev, 9, 733-6
46. Chen HC, Hu WX, Liu QX, et al. (2008) Genetic polymorphisms of metabolic enzymes CYP1A1, CYP2D6, GSTM1 and GSTT1 and leukemia susceptibility. Eur J Cancer Prev 17:251–258
47. Taspinar M, Aydos SE, Comez O, et al. (2008) CYP1A1, GST gene polymorphisms and risk of chronic myeloid leukemia. Swiss Med Wkly 138:12–17.
48. Rimando MG, Chua MN, Y uson E, de Castro-Bernas G, Okamoto T.Prevalence of GSTT1, GSTM1 and NQO1 (609C>T) in Filipino children with all (acute lymphoblastic leukaemia). Biosci Rep.2008;28:117–24
49. Gra OA, Glotov AS, Kozhekbaeva Z, Makarova OV, Nasedkina TV. Genetic polymorphism in GST, NAT2, and MTRR and susceptibility to childhood acute leukemia. Mol Biol (Mosk) 2008;42:214–25.
50. Souza CL, Barbosa CG, Moura NetoI JP, et al. (2008) Poly-morphisms in the glutathione S-transferase theta and mu genes and susceptibility to myeloid leukemia in Brazilian patients. Genet Mol Biol 31:39–41
51. Jiang LJ, Tan GF. Association between the polymorphisms of cyto-chrome p4501a1 and glutathione S-transferase M1, T1 genes and childhood acute lymphocytic leukemia. Chin J New Clin Med. 2010;3:810–4
52. Sailaja K, Surekha D, Rao DN, Rao DR, Vishnupriya S. Association of the GSTP1 gene (Ile105Val) polymorphism with chronic myeloid leukemia. Asian Pac J Cancer Prev. 2010;11(2):461-464
53. Ovsepian VA, EIu V, Sherstneva E S. Cytochrome P4501A1, glutathione S-transferase M1 and T1 gene polymorphisms in chronic myeloid leukemia[J]. Genetika, 2010, 46(10): 1360-1362.
54. Sailaja K, Surekha D, Rao DN, Rao DR, Vishnupriya S. Association of the GSTP1 gene (Ile105Val) polymorphism with chronic myeloid leukemia. Asian Pac J Cancer Prev. 2010;11(2):461-464
55. Chan JY, Ugrasena DG, Lum DW, Lu Y, Yeoh AE (2011) Xenobiotic and folate pathway gene polymorphisms and risk of childhood acute lymphoblastic leukaemia in Javanese children. Hematol Oncol 29:116-123. doi:10.1002/hon.965. PubMed: 20824655.
56. Suneetha KJ, Nancy KN, Rajalekshmy KR, et al. (2011). Role of glutathione-s-transferase and CYP1A1*2A polymorphisms in the therapy outcome of south Indian acute lymphoblastic leukemia patients. Indian J Med Paediatr Oncol, 32, 25-9
57. Ouerhani S, Nefzi MA, Menif S, Safra I, Douzi K, Fouzai C, Ben Ghorbel G, Ben Bahria I, Ben Ammar Elgaaied A, Abbes S. Influence of genetic polymorphisms of xenobiotic metabolizing enzymes on the risk of developing leukemia in a Tunisian population. Bull Cancer. 2011 Dec;98(12):95-106. doi: 10.1684/bdc.2011.1502. PMID: 22146408
58. Xi YM, Shi XE, Zhang H, et al. Relation of GSTP1 and CYP2E1 polymorphisms with susceptibility to acute leukemia. Zhongguo Shi Yan Xue Ye Xue Za Zhi 2011;19:589– 93.
59. Mandegary A, Rostami S, Alimoghaddam K, Ghavamzadeh A, Ghahremani MH. Gluthatione-S-transferase T1-null genotype predisposes adults to acute promyelocytic leukemia; a case-control study. Asian Pac J Cancer Prev. 2011;12(5):1279-82. PMID: 21875282
60. Chauhan PS, Ihsan R, Yadav DS, et al. Association of glutathione S-transferase, EPHX, and p53 codon 72 gene polymorphisms with adult acute myeloid leukemia. DNA Cell Biol 2011;30:39– 46.
61. Chauhan PS, Ihsan R, Mishra AK, et al. High order interactions of xenobiotic metabolizing genes and P53 codon 72 polymorphisms in acute leukemia. Environ Mol Mutagen 2012;53:619–30
62. Kim HN, Kim NY , Y u L, et al (2012). Association of GSTT1 polymorphism with acute myeloid leukemia risk is dependent on smoking status. Leuk Lymphoma, 53, 681-7.
63. Li YH, Wen FQ, Xiao ZH, et al. (2012). Genetic polymorphism of GST gene in children with infectious mononucleosis and acute lymphoblastic leukemia. Chin J Contemp Pediatr, 4, 260-63.
64. Bhat G, Bhat A, Wani A, et al. Polymorphic variation in glutathione-S-transferase genes and risk of chronic myeloid leukaemia in the Kashmiri population. Asian Pac J Cancer Prev. 2012;13(1):69-73. doi:10.7314/apjcp.2012.13.1.0691
65. Lordelo GS, Miranda-Vilela AL, Akimoto AK, Alves PC, Hiragi CO, Nonino A, Daldegan MB, Klautau-Guimarães MN, Grisolia CK. Association between methylene tetrahydrofolate reductase and glutathione S-transferase M1 gene polymorphisms and chronic myeloid leukemia in a Brazilian population. Genet Mol Res. 2012 Apr 19;11(2):1013-26. doi: 10.4238/2012.April.19.6. PMID: 22576927
66. Özten N, Sunguroğlu A, Bosland MC. Variations in glutathione-S-transferase genes influence risk of chronic myeloid leukemia. Hematol Oncol. 2012 Sep;30(3):150-5. doi: 10.1002/hon.1018. Epub 2011 Oct 4. PMID: 21969307
67. Karkucak M, Yakut T, Gulten T, et al. Investigation of GSTP1 (Ile105Val) gene polymorphism in chronic myeloid leukaemia patients[J]. International Journal of Human Genetics, 2012, 12(3): 145-149.
68. Dunna NR, Vuree S, Kagita S, et al. Association of GSTP1 gene (I105V) polymorphism with acute leukaemia. J Genet 2012;91:e60– 3.
69. Dunna NR, Vure S, Sailaja K, et al (2013). Deletion of GSTM1 and T1 genes as a risk factor for development of acute leukemia. Asian Pac J Cancer Prev, 14, 2221-4
70. Hou W, Chen BL. The relationship between GSTT1, GSTM1 and MDR1 gene polymorphisms and susceptibility to childhood acute lymphoblastic leukemia and chemotherapy response. Journal of Hainan medical college. 2013; 19( 11) : 1493 －1496
71. Zhou L, Zhu YY, Zhang XD, Li Y, Liu ZG. Risk effects of GST gene polymorphisms in patients with acute myeloid leukemia: a prospective study. Asian Pac J Cancer Prev 2013;14:3861– 4.
72. Moulik NR，Parveen F，Kumar A，et al． Glutathione － S － transfer-ase polymorphism and acute lymphoblastic leukemia ( ALL) in north Indian children: a case － control study and meta － analysis． Journal of Human Genetics， 2014; 59( 9) : 526 －529
73. Al-Achkar W, Azeiz G, Moassass F, Wafa A. Influence of CYP1A1, GST polymorphisms and susceptibility risk of chronic myeloid leukemia in Syrian population. Med Oncol. 2014 May;31(5):889. doi: 10.1007/s12032-014-0889-4. Epub 2014 Mar 27. PMID: 24671854
74. Zi Y, Wu S, Ma D, Yang C, Yang M, Huang Y, Yang SJ. Association of GSTTI and GSTM1 variants with acute myeloid leukemia risk. Genet Mol Res. 2014 May 9;13(2):3681-5. doi: 10.4238/2014.May.9.11. PMID: 24854448.
75. Guven M, Unal S, Erhan D, Ozdemir N, Baris S, Celkan T, Bostancı M, Batar B. Role of glutathione S-transferase M1, T1 and P1 gene polymorphisms in childhood acute lymphoblastic leukemia susceptibility in a Turkish population. Meta Gene. 2015 Jun 17;5:115-9. doi: 10.1016/j.mgene.2015.06.002. PMID: 26137447; PMCID: PMC4484718
76. Kassogue Y, Dehbi H, Quachouh M, Quessar A, Benchekroun S, Nadifi S. Association of glutathione S-transferase (GSTM1 and GSTT1) genes with chronic myeloid leukemia. Springerplus. 2015 May 1;4:210. doi: 10.1186/s40064-015-0966-y. PMID: 25969820; PMCID: PMC4417468
77. Nasr AS, Sami RM, Ibrahim NY, Darwish DO. Glutathione S transferase (GSTP 1, GSTM 1, and GSTT 1) gene polymorphisms in Egyptian patients with acute myeloid leukemia. Indian J Cancer. 2015 Oct-Dec;52(4):490-5. doi: 10.4103/0019-509X.178408. PMID: 26960454.
78. Liu P, Zhang M, Xie X, Jin J, Holman CD. Green tea consumption and glutathione S-transferases genetic polymorphisms on the risk of adult leukemia. Eur J Nutr. 2017 Mar;56(2):603-612. doi: 10.1007/s00394-015-1104-x. Epub 2015 Nov 17. PMID: 26578531.
79. Bănescu C, Iancu M, Trifa AP, Cândea M, Benedek Lazar E, Moldovan VG, Duicu C, Tripon F, Crauciuc A, Dobreanu M. From Six Gene Polymorphisms of the Antioxidant System, Only GPX Pro198Leu and GSTP1 Ile105Val Modulate the Risk of Acute Myeloid Leukemia. Oxid Med Cell Longev. 2016;2016:2536705. doi: 10.1155/2016/2536705. Epub 2015 Dec 28. PMID: 26823947; PMCID: PMC4707325.
80. Weich N, Ferri C, Moiraghi B, Bengió R, Giere I, Pavlovsky C, Larripa IB, Fundia AF. GSTM1 and GSTP1, but not GSTT1 genetic polymorphisms are associated with chronic myeloid leukemia risk and treatment response. Cancer Epidemiol. 2016 Oct;44:16-21. doi: 10.1016/j.canep.2016.07.008. Epub 2016 Jul 25. PMID: 27454607.
81. Al-Eitan LN, Rababaʼh DM, Alkhatib RQ, Khasawneh RH, ALjarrah OA. GSTM1 and GSTP1 Genetic Polymorphisms and Their Associations With Acute Lymphoblastic Leukemia Susceptibility in a Jordanian Population. J Pediatr Hematol Oncol. 2016 Oct;38(7):e223-9. doi: 10.1097/MPH.0000000000000609. PMID: 27299594.
82. Zehra A, Zehra S, Ismail M, Azhar A. Glutathione S-Transferase M1 and T1 Gene Deletions and Susceptibility to Acute Lymphoblastic Leukemia (ALL) in adults. Pak J Med Sci. 2018 May-Jun;34(3):666-670. doi: 10.12669/pjms.343.14911. PMID: 30034435; PMCID: PMC6041525
83. Brisson GD, de Almeida Lopes B, Andrade FG, Dos Santos Bueno FV, Sardou-Cezar I, de Aguiar Gonçalves BA, Terra-Granado E, Paraguassú-Braga FH, Pombo-de-Oliveira MS. EPHX1 rs1051740 T>C (Tyr113His) is strongly associated with acute myeloid leukemia and KMT2A rearrangements in early age. Arch Toxicol. 2018 Jun; 92(6):2001-2012. doi: 10.1007/s00204-018-2198-8. Epub 2018 Mar 31. PMID: 29605894.
84. Farasani A. Genetic variants of glutathione S-transferase and the risk of acute myeloid leukemia in a Saudi population. Saudi J Biol Sci. 2019 Nov;26(7):1525-1530. doi: 10.1016/j.sjbs.2018.12.011. Epub 2018 Dec 21. PMID: 31762621; PMCID: PMC6864205
85. Muddathir ARM, Abdallah EI, Khabour OF, Abdelgader RE, Elgari MM. Age- and gender-independent association of glutathione S-transferase null polymorphisms with chronic myeloid leukemia. Bosn J Basic Med Sci. 2019 Apr 15;19(4):350-354. doi: 10.17305/bjbms.2019.4176. PMID: 30995900; PMCID: PMC6868479
86. Rostami G, Assad D, Ghadyani F, Hamid M, Karami A, Jalaeikhoo H, Kalahroodi RA. Influence of glutathione S-transferases (GSTM1, GSTT1, and GSTP1) genetic polymorphisms and smoking on susceptibility risk of chronic myeloid leukemia and treatment response. Mol Genet Genomic Med. 2019 Jul;7(7):e00717. doi: 10.1002/mgg3.717. Epub 2019 May 20. PMID: 31111691; PMCID: PMC6625153
87. Baba SM, Shah ZA, Pandith AA, Geelani SA, Mir MM, Bhat JR, Gul A, Bhat GM. Glutathione S-transferase gene polymorphic sequence variations: Association with risk and response to Imatinib among Chronic Myeloid Leukemia patients of Kashmir. Int J Lab Hematol. 2021 Oct;43(5):1000-1008. doi: 10.1111/ijlh.13471. Epub 2021 Jan 20. PMID: 33470551.
88. Idris HM, Elderdery AY, Khalil HB, Mills J. Genetic Polymorphism of GSTP1, GSTM1 and GSTT1 Genes and Susceptibility to Chronic Myeloid Leukaemia. Asian Pac J Cancer Prev. 2020 Feb 1;21(2):499-503. doi: 10.31557/APJCP.2020.21.2.499. PMID: 32102530; PMCID: PMC7332153
89. Baba SM, Pandith AA, Shah ZA, Geelani SA, Bhat JR, Gul A, Guru SA, El-Serehy HA, Koul AM, Mansoor S. GSTT1 null and rs156697 Polymorphism in GSTO2 Influence the Risk and Therapeutic Outcome of B-Acute Lymphoblastic Leukemia Patients. Front Oncol. 2021 Oct 14;11:714421. doi: 10.3389/fonc.2021.714421. PMID: 34722260; PMCID: PMC8552530.
90. Abdalhabib EK, Jackson DE, Alzahrani B, Elfaki EM, Hamza A, Alanazi F, Ali EI, Algarni A, Ibrahim IK, Saboor M. Combined GSTT1 Null, GSTM1 Null and XPD Lys/Lys Genetic Polymorphisms and Their Association with Increased Risk of Chronic Myeloid Leukemia. Pharmgenomics Pers Med. 2021 Dec 22;14:1661-1667. doi: 10.2147/PGPM.S342625. PMID: 34992428; PMCID: PMC8710912.
91. Abdalhabib EK, Alzahrani B, Alanazi F, Algarni A, Ibrahim IK, Mohamed HA, Hamali HA, Mobarki AA, Dobie G, Saboor M. Increased Risk of Acute Lymphoblastic Leukemia in Adult Patients with GSTM1 Null Genetic Polymorphism. Pharmgenomics Pers Med. 2022 Mar 15;15:227-234. doi: 10.2147/PGPM.S356302. PMID: 35313604; PMCID: PMC8934168.
