# Supplementary material for: Individual and combined effects of the GSTM1, GSTT1, and GSTP1 polymorphisms on leukemia risk: An updated meta-analysis
Source: Front Genet. 2022 Oct 31;13:976673. doi: 10.3389/fgene.2022.976673 (PMC9659912; doi:10.3389/fgene.2022.976673)
Supplement: Supplementary file 2 [file Table2.DOCX]

**Supplemental Table1** Main characteristics and Quality score of studies included.

| **First Author/Year** | **Type of leukemia** | **Age group** | **Ethnicity** | **Sample size** | **Type of controls** | **Matching** | **Genotypes of GSTM1** | | | | **score** |
| --- | --- | --- | --- | --- | --- | --- | --- | --- | --- | --- | --- |
|  |  |  |  |  |  |  | **Cases** | | **Controls** | |  |
|  |  |  |  |  |  |  | null | present | null | present |  |
| Basu T [1] (1997) | ALL | Children | Asian | 67/146 | Healthy controls | NO | 51 | 16 | 77 | 69 | 9 |
| Chen CL [2] (1997) | ALL | Children | Caucasian | 163/213 | Healthy controls | NO | 90 | 73 | 114 | 99 | 8 |
| Chen CL [2] (1997) | ALL | Children | African | 34/203 | Healthy controls | NO | 14 | 20 | 56 | 147 | 8 |
| Krajinovic M [3] (1999) | ALL | Children | Caucasian | 177/304 | Healthy controls | NO | 113 | 61 | 156 | 148 | 9 |
| Sasai Y [4] (1999) | AML | Adults | Asian | 65/43 | Healthy controls | NO | 32 | 33 | 23 | 20 | 8 |
| Lemos MC [5] (1999) | ALL | Adults+Children | Caucasian | 22/128 | Healthy controls | NO | 14 | 8 | 74 | 54 | 7 |
| Lemos MC [5] (1999) | CLL | Adults+Children | Caucasian | 13/128 | Healthy controls | NO | 5 | 8 | 74 | 54 | 7 |
| Lemos MC [5] (1999) | AML | Adults+Children | Caucasian | 18/128 | Healthy controls | NO | 10 | 8 | 74 | 54 | 7 |
| Lemos MC [5] (1999) | CML | Adults+Children | Caucasian | 11/128 | Healthy controls | NO | 9 | 3 | 74 | 54 | 7 |
| Saadat I [6] (2000) | ALL | Children | Caucasian | 38/75 | Healthy controls | NO | 21 | 17 | 24 | 51 | 8 |
| Woo MH [7] (2000) | AML | Children | Mixed | 57/245 | ALL | NO | 18 | 39 | 104 | 141 | 9 |
| Crump C [8] (2000) | AML | Adults | Caucasian | 297/152 | Non-blood disease controls | NO | 159 | 138 | 75 | 77 | 13 |
| Rollinson S [9] (2000) | AML | Adults | Caucasian | 479/827 | Non-blood disease controls | Age and sex | 258 | 221 | 407 | 420 | 13 |
| Rollinson S [9] (2000) | ALL | Adults | Caucasian | 71/114 | Non-blood disease controls | Age and sex | 35 | 36 | 55 | 59 | 11 |
| Naoe T [10] (2000) | AML | Adults | Asian | 411/150 | Healthy controls | NO | 227 | 184 | 77 | 73 | 10 |
| Lo ¨ffler H [11] (2001) | CML | Adults | Caucasian | 141/150 | Healthy controls | NO | 77 | 64 | 84 | 66 | 9 |
| Arruda VRL [12] (2001) | AML | Adults | Mixed | 38/276 | Non-blood disease controls | NO | 28 | 10 | 102 | 174 | 8 |
| Allan JM [13] (2001) | AML | Adults | Caucasian | 420/1022 | Non-blood disease controls | Age and sex | 229 | 188 | 496 | 523 | 12 |
| Krajinovic M [14] (2002) | ALL | Children | Caucasian | 269/301 | Healthy controls | NO | 151 | 118 | 141 | 160 | 10 |
| Haase D [15] (2002) | AML | Adults | Caucasian | 213/239 | Healthy controls | NO | 107 | 106 | 122 | 117 | 10 |
| Alves S [16] (2002) | ALL | Children | Caucasian | 47/102 | Healthy controls | NO | 32 | 15 | 50 | 52 | 8 |
| Davies SM [17] (2002) | ALL | Children | Caucasian | 616/532 | Non-blood disease controls | NO | 331 | 285 | 286 | 246 | 11 |
| Davies SM [17] (2002) | ALL | Children | African | 35/201 | Non-blood disease controls | NO | 14 | 21 | 64 | 137 | 10 |
| Yuille M [18] (2002) | CLL | Adults | Caucasian | 138/280 | Non-blood disease controls | NO | 77 | 61 | 135 | 135 | 10 |
| Balta G [19] (2003) | ALL | Children | Caucasian | 139/185 | Healthy controls | NO | 77 | 62 | 101 | 84 | 9 |
| Zhang L [20] (2003) | ALL | Children | Asian | 67/146 | Healthy controls | NO | 51 | 16 | 77 | 69 | 8 |
| Zhang L [20] (2003) | AML | Children | Asian | 32/146 | Healthy controls | NO | 23 | 9 | 77 | 69 | 7 |
| Wang J [22] (2004) | ALL | Children | Asian | 67/146 | Healthy controls | NO | 51 | 16 | 77 | 69 | 9 |
| Zou LL [23] (2004) | ALL | Adults+Children | Asian | 16/183 | Healthy controls | NO | 10 | 6 | 99 | 84 | 8 |
| Zou LL [23] (2004) | AML | Adults | Asian | 25/183 | Healthy controls | NO | 17 | 8 | 99 | 84 | 9 |
| Canalle R [24] (2004) | ALL | Children | Mixed | 113/221 | Non-blood disease controls | NO | 48 | 65 | 101 | 120 | 9 |
| Seedhouse C [25] (2004) | AML | Adults+Children | Caucasian | 200/177 | Non-blood disease controls | NO | 101 | 99 | 78 | 99 | 11 |
| Joseph T [26] (2004) | ALL | Children | Indian | 118/118 | Non-blood disease controls | Age and sex | 48 | 70 | 29 | 89 | 10 |
| D’Alo F [27] (2004) | AML | Adults | Caucasian | 193/73 | Healthy controls | NO | 82 | 111 | 128 | 145 | 10 |
| Liu QX [28] (2005) | ALL | Adults+Children | Asian | 112/204 | Healthy controls | NO | 68 | 44 | 113 | 91 | 10 |
| Mondal BC [29] (2005) | CML | Adults+Children | Indian | 81/123 | Non-blood disease controls | NO | 23 | 58 | 34 | 89 | 9 |
| Clavel J [30] (2005) | ALL | Children | Caucasian | 191/105 | Non-blood disease controls | NO | 94 | 97 | 50 | 55 | 9 |
| Pakakasama S [31] (2005) | ALL | Children | Asian | 107/320 | Healthy controls | NO | 76 | 31 | 191 | 129 | 9 |
| Hishida A [32] (2005) | CML | Adults | Asian | 51/476 | Healthy controls | NO | 26 | 25 | 249 | 227 | 11 |
| Yang L [33] (2005) | AML | Adults | Asian | 228/241 | Healthy controls | Age and sex | 142 | 86 | 127 | 114 | 12 |
| Aydin-Sayitoglu M [34] (2006) | AML | Adults+Children | Caucasian | 94/140 | Non-blood disease controls | NO | 64 | 30 | 77 | 63 | 9 |
| Aydin-Sayitoglu M [34] (2006) | ALL | Adults+Children | Caucasian | 155/140 | Non-blood disease controls | NO | 97 | 58 | 77 | 63 | 9 |
| Bajpai P [35] (2007) | CML | Adults | Indian | 80/105 | Healthy controls | NO | 24 | 56 | 26 | 79 | 11 |
| Pigullo S [36] (2007) | ALL | Children | Caucasian | 323/384 | Non-blood disease controls | NO | 152 | 171 | 200 | 184 | 9 |
| Bolufer P [37] (2007) | AML | Adults+Children | Caucasian | 302/454 | Non-blood disease controls | NO | 143 | 159 | 232 | 222 | 12 |
| Bolufer P [37] (2007) | ALL | Adults+Children | Caucasian | 141/454 | Non-blood disease controls | NO | 64 | 77 | 232 | 222 | 12 |
| Eyada TK [39] (2007) | AML | unknown | Caucasian | 19/11 | Healthy controls | NO | 13 | 6 | 0 | 11 | 7 |
| Eyada TK [39] (2007) | ALL | unknown | Caucasian | 13/11 | Healthy controls | NO | 6 | 7 | 0 | 11 | 7 |
| Bhatla D [41] (2008) | AML | Children | Caucasian | 461/646 | Healthy controls | NO | 218 | 243 | 299 | 347 | 12 |
| Majumdar S [42] (2008) | AML | Adults+Children | Indian | 110/143 | Healthy controls | NO | 57 | 53 | 34 | 109 | 11 |
| Muller P [43] (2008) | AML | Adults | Caucasian | 136/217 | Non-blood disease controls | NO | 70 | 66 | 119 | 98 | 8 |
| Jiang LJ [44] (2008) | ALL | Adults | Asian | 88/120 | Healthy controls | Age and sex | 53 | 35 | 65 | 55 | 12 |
| Suneetha KJ [45] (2008) | ALL | Adults+Children | Indian | 92/150 | Non-blood disease controls | NO | 36 | 56 | 37 | 113 | 8 |
| Chen HC [46] (2008) | ALL | Adults+Children | Asian | 120/204 | Healthy controls | NO | 72 | 48 | 113 | 91 | 10 |
| Chen HC [46] (2008) | CML | Adults+Children | Asian | 108/204 | Healthy controls | NO | 58 | 50 | 113 | 91 | 10 |
| Taspinar M [47] (2008) | CML | Adults | Caucasian | 107/130 | Healthy controls | NO | 48 | 59 | 55 | 75 | 8 |
| Rimando MG [48] (2008) | ALL | Children | Asian | 60/60 | Healthy controls | NO | 43 | 17 | 31 | 29 | 7 |
| Gra OA[49] (2008) | ALL | Children | Caucasian | 332/490 | Healthy controls | NO | 181 | 151 | 238 | 252 | 10 |
| Gra OA [49] (2008) | AML | Children | Caucasian | 71/490 | Healthy controls | NO | 38 | 33 | 238 | 252 | 10 |
| Souza CL[50] (2008) | AML | Adults+Children | Mixed | 23/304 | Non-blood disease controls | NO | 7 | 16 | 100 | 104 | 10 |
| Souza CL [50] (2008) | CML | Adults+Children | Mixed | 53/304 | Non-blood disease controls | NO | 15 | 38 | 100 | 104 | 10 |
| Jiang LJ [51] (2010) | ALL | Children | Asian | 89/90 | Healthy controls | NO | 68 | 21 | 49 | 41 | 9 |
| Ovsepian [53] (2010) | CML | Adults | Caucasian | 83/205 | Healthy controls | NO | 44 | 39 | 94 | 111 | 8 |
| Chan JY [55] (2011) | ALL | Children | Asian | 185/177 | Healthy controls | NO | 142 | 43 | 122 | 55 | 10 |
| Ouerhani S [57] (2011) | leukemia | Adults+Children | Caucasian | 193/309 | Healthy controls | NO | 110 | 83 | 163 | 146 | 13 |
| Mandegary [59] (2011) | AML | Adults | Caucasian | 114/99 | Healthy controls | NO | 64 | 50 | 48 | 51 | 10 |
| Chauhan PS [60] (2011) | AML | Adults | Indian | 120/202 | Healthy controls | Age and sex | 45 | 75 | 97 | 105 | 12 |
| Chauhan PS [61] (2012) | AML | Adults+Children | Indian | 131/199 | Healthy controls | Age and sex | 50 | 81 | 95 | 104 | 12 |
| Chauhan PS [61] (2012) | ALL | Adults+Children | Indian | 99/199 | Healthy controls | Age and sex | 36 | 63 | 95 | 104 | 12 |
| Kim HN [62] (2012) | AML | Adults | Asian | 415/1700 | Non-blood disease controls | NO | 230 | 185 | 923 | 777 | 10 |
| Li YH [63] (2012) | ALL | Children | Asian | 41/100 | Non-blood disease controls | NO | 28 | 13 | 49 | 51 | 8 |
| Bhat G [64] (2012) | CML | Adults | Indian | 75/124 | Non-blood disease controls | Age and gender | 31 | 44 | 43 | 81 | 12 |
| Lordelo [65] (2012) | CML | Adults | Mixed | 105/273 | Healthy controls | NO | 55 | 50 | 176 | 97 | 9 |
| Ozten N [66] (2012) | CML | Adults | Caucasian | 106/190 | Healthy controls | Age and gender | 48 | 58 | 81 | 109 | 11 |
| Dunna NR [69] (2013) | AML | Adults+Children | Indian | 142/251 | Non-blood disease controls | Age and sex | 90 | 52 | 94 | 157 | 9 |
| Dunna NR [69] (2013) | ALL | Adults+Children | Indian | 152/251 | Non-blood disease controls | Age and sex | 89 | 63 | 94 | 157 | 9 |
| Hou W [70] (2013) | ALL | Children | Asian | 100/112 | Non-blood disease controls | NO | 72 | 28 | 51 | 61 | 9 |
| Zhou L [71] (2013) | AML | unknown | Asian | 163/204 | Non-blood disease controls | Age | 86 | 77 | 97 | 107 | 10 |
| Moulik NR [72] (2014) | ALL | Children | Indian | 100/300 | Healthy controls | NO | 35 | 65 | 84 | 216 | 10 |
| Al-Achkar [73] (2014) | CML | Adults | Caucasian | 126/172 | Non-blood disease controls | NO | 54 | 72 | 39 | 133 | 10 |
| Zi Y [74] (2014) | AML | unknown | Asian | 206/231 | Non-blood disease controls | NO | 114 | 92 | 107 | 124 | 9 |
| Guven [75] (2015) | ALL | Children | Caucasian | 95/190 | Healthy controls | NO | 45 | 50 | 99 | 91 | 9 |
| Kassogue Y [76] (2015) | CML | Adults | Caucasian | 92/93 | Healthy controls | NO | 45 | 47 | 38 | 55 | 10 |
| Nasr AS [77] (2015) | AML | Adults | Caucasian | 50/50 | Healthy controls | Age and sex | 24 | 26 | 7 | 43 | 10 |
| Liu P [78] (2015) | leukemia | Adults | Asian | 442/442 | Non-blood disease controls | gender | 248 | 176 | 227 | 193 | 12 |
| Bsnescu C [79] (2016) | AML | Adults | Caucasian | 102/303 | Healthy controls | NO | 59 | 43 | 178 | 125 | 11 |
| Weich [80] (2016) | CML | Adults | Caucasian | 141/141 | Healthy controls | Age and sex | 56 | 85 | 57 | 84 | 11 |
| Al-Eitan LN [81] (2016) | ALL | Children | Caucasian | 88/176 | Healthy controls | NO | 58 | 30 | 108 | 68 | 9 |
| Zehra A [82] (2018) | ALL | Adults | Caucasian | 62/62 | Healthy controls | Age and gender | 29 | 33 | 28 | 34 | 10 |
| Brisson GD [83] (2018) | AML | Children | Mixed | 95/397 | Healthy controls | NO | 35 | 60 | 160 | 237 | 12 |
| Farasani A [84] (2019) | AML | Adults | Caucasian | 100/100 | Healthy controls | NO | 34 | 66 | 44 | 56 | 9 |
| Muddathir ARM [85] (2019) | CML | Adults | African | 115/104 | Healthy controls | NO | 68 | 47 | 42 | 62 | 10 |
| Rostami G [86] (2019) | CML | Adults | Caucasian | 104/104 | Healthy controls | Age and sex | 70 | 34 | 51 | 53 | 10 |
| Baba SM [87] (2020) | CML | Adults+Children | Indian | 150/150 | Healthy controls | NO | 36 | 114 | 56 | 94 | 10 |
| Idris HM [88] (2020) | CML | unknown | African | 200/100 | Healthy controls | Age | 68 | 132 | 35 | 65 | 10 |
| Baba SM [89] (2021) | ALL | Adults+Children | Indian | 150/150 | Healthy controls | Age and gender | 43 | 107 | 56 | 94 | 11 |
| Abdalhabib EK [90] (2021) | CML | Adults | African | 150/150 | Healthy controls | Age and gender | 92 | 58 | 53 | 97 | 12 |
| Abdalhabib EK [91] (2022) | ALL | Adults | African | 128/128 | Healthy controls | Age | 59 | 69 | 24 | 104 | 13 |
