# Supplementary material for: Individual and combined effects of the GSTM1, GSTT1, and GSTP1 polymorphisms on leukemia risk: An updated meta-analysis
Source: Front Genet. 2022 Oct 31;13:976673. doi: 10.3389/fgene.2022.976673 (PMC9659912; doi:10.3389/fgene.2022.976673)
Supplement: Supplementary file 3 [file Table5.DOCX]

**Supplemental Table 4** Scale for quality assessment of molecular association studies of leukemia.

| Criterion | Score |
| --- | --- |
| Source of case | |
| Selected from population or cancer registry | 2 |
| Selected from hospital | 1 |
| Not described | 0 |
| Source of control | |
| Population-based | 2 |
| Hospital-based | 1 |
| Not described | 0 |
| Ascertainment of cancer | |
| Histological or pathological confirmation | 2 |
| Diagnosis of leukemia by patient medical record | 1 |
| Not described | 0 |
| Ascertainment of control | |
| Controls were tested to screen out leukemia | 2 |
| Controls were subjects who did not report blood disease, no objective testing | 1 |
| Not described | 0 |
| Matching | |
| Controls matched with cases by age and sex | 2 |
| Controls matched with cases only by age or sex | 1 |
| Not matched or not described | 0 |
| Genotyping examination | |
| Genotyping done blindly and quality control | 2 |
| Only genotyping done blindly or quality control | 1 |
| Unblinded and without quality control | 0 |
| HWE | |
| HWE in the control group | 2 |
| Hardy-Weinberg disequilibrium in the control group | 0 |
| Association assessment | |
| Assess association between genotypes and leukemia with appropriate statistics and adjustment for confounders | 2 |
| Assess association between genotypes and leukemia with appropriate statistics without adjustment for confounders | 1 |
| Inappropriate statistics used | 0 |
| Total sample size |  |
| ≥200 | 2 |
| <200 | 0 |

HWE: Hardy-Weinberg equilibrium
