# Supplementary material for: Individual and combined effects of the GSTM1, GSTT1, and GSTP1 polymorphisms on leukemia risk: An updated meta-analysis
Source: Front Genet. 2022 Oct 31;13:976673. doi: 10.3389/fgene.2022.976673 (PMC9659912; doi:10.3389/fgene.2022.976673)
Supplement: Supplementary file 4 [file Table3.DOCX]

**Supplemental Table 2** Main characteristics and Quality score of studies included.

| **First Author/Year** | **Type of leukemia** | **Age group** | **Ethnicity** | **Sample size** | **Type of controls** | **Matching** | **Genotypes of GSTT1** | | | | | **score** |
| --- | --- | --- | --- | --- | --- | --- | --- | --- | --- | --- | --- | --- |
|  |  |  |  |  |  |  | **Cases** | | | **Controls** | |  |
|  |  |  |  |  |  |  | null | present | | null | present |  |
| Basu T [1] (1997) | ALL | Children | Asian | 67/146 | Healthy controls | NO | 41 | | 26 | 72 | 74 | 9 |
| Chen CL [2] (1997) | ALL | Children | Caucasian | 163/213 | Healthy controls | NO | 23 | | 140 | 32 | 181 | 8 |
| Chen CL [2] (1997) | ALL | Children | African | 34/203 | Healthy controls | NO | 12 | | 22 | 49 | 154 | 8 |
| Krajinovic M [3] (1999) | ALL | Children | Caucasian | 177/304 | Healthy controls | NO | 28 | | 148 | 47 | 227 | 9 |
| Sasai Y [4] (1999) | AML | Adults | Asian | 65/43 | Healthy controls | NO | 33 | | 32 | 13 | 30 | 8 |
| Woo MH [7] (2000) | AML | Children | Mixed | 57/245 | ALL | NO | 6 | | 51 | 21 | 224 | 9 |
| Crump C [8] (2000) | AML | Adults | Caucasian | 297/152 | Non-blood disease controls | NO | 48 | | 249 | 26 | 126 | 13 |
| Rollinson S [9] (2000) | AML | Adults | Caucasian | 479/827 | Non-blood disease controls | Age and sex | 89 | | 390 | 125 | 702 | 13 |
| Rollinson S [9] (2000) | ALL | Adults | Caucasian | 71/114 | Non-blood disease controls | Age and sex | 15 | | 56 | 9 | 105 | 11 |
| Naoe T [10] (2000) | AML | Adults | Asian | 411/150 | Healthy controls | NO | 197 | | 214 | 81 | 69 | 10 |
| Lo ¨ffler H [11] (2001) | CML | Adults | Caucasian | 141/150 | Healthy controls | NO | 31 | | 110 | 26 | 124 | 9 |
| Arruda VRL [12] (2001) | AML | Adults | Mixed | 38/276 | Non-blood disease controls | NO | 13 | | 25 | 44 | 232 | 8 |
| Allan JM [13] (2001) | AML | Adults | Caucasian | 420/1022 | Non-blood disease controls | Age and sex | 79 | | 338 | 140 | 879 | 12 |
| Haase D [15] (2002) | AML | Adults | Caucasian | 213/239 | Healthy controls | NO | 49 | | 164 | 38 | 201 | 10 |
| Alves S [16] (2002) | ALL | Children | Caucasian | 47/102 | Healthy controls | NO | 9 | | 38 | 26 | 76 | 8 |
| Davies SM [17] (2002) | ALL | Children | Caucasian | 616/532 | Non-blood disease controls | NO | 96 | | 520 | 87 | 445 | 11 |
| Davies SM [17] (2002) | ALL | Children | African | 35/201 | Non-blood disease controls | NO | 6 | | 29 | 56 | 145 | 10 |
| Yuille M [18] (2002) | CLL | Adults | Caucasian | 138/280 | Non-blood disease controls | NO | 41 | | 97 | 66 | 212 | 10 |
| Balta G [19] (2003) | ALL | Children | Caucasian | 139/185 | Healthy controls | NO | 29 | | 110 | 42 | 143 | 9 |
| Zhang L [20] (2003) | ALL | Children | Asian | 67/146 | Healthy controls | NO | 41 | | 26 | 72 | 74 | 8 |
| Zhang L [20] (2003) | AML | Children | Asian | 32/146 | Healthy controls | NO | 19 | | 13 | 72 | 74 | 7 |
| Wang J [22] (2004) | ALL | Children | Asian | 67/146 | Healthy controls | NO | 41 | | 26 | 72 | 74 | 9 |
| Zou LL [23] (2004) | ALL | Adults+Children | Asian | 16/183 | Healthy controls | NO | 14 | | 2 | 89 | 94 | 8 |
| Zou LL [23] (2004) | AML | Adults | Asian | 25/183 | Healthy controls | NO | 14 | | 11 | 89 | 94 | 9 |
| Canalle R [24] (2004) | ALL | Children | Mixed | 113/221 | Non-blood disease controls | NO | 25 | | 88 | 43 | 178 | 9 |
| Joseph T [26] (2004) | ALL | Children | Indian | 118/118 | Non-blood disease controls | Age and sex | 17 | | 101 | 10 | 108 | 10 |
| D’Alo F [27] (2004) | AML | Adults | Caucasian | 193/73 | Healthy controls | NO | 56 | | 137 | 52 | 221 | 10 |
| Liu QX [28] (2005) | ALL | Adults+Children | Asian | 112/204 | Healthy controls | NO | 57 | | 55 | 100 | 104 | 10 |
| Mondal BC [29] (2005) | CML | Adults+Children | Indian | 81/123 | Non-blood disease controls | NO | 16 | | 65 | 9 | 114 | 9 |
| Clavel J [30] (2005) | ALL | Children | Caucasian | 191/105 | Non-blood disease controls | NO | 42 | | 149 | 23 | 82 | 9 |
| Pakakasama S [31] (2005) | ALL | Children | Asian | 107/320 | Healthy controls | NO | 50 | | 57 | 122 | 198 | 9 |
| Hishida A [32] (2005) | CML | Adults | Asian | 51/476 | Healthy controls | NO | 29 | | 22 | 238 | 238 | 11 |
| Yang L [33] (2005) | AML | Adults | Asian | 228/241 | Healthy controls | Age and sex | 110 | | 118 | 108 | 133 | 12 |
| Aydin-Sayitoglu M [34] (2006) | AML | Adults+Children | Caucasian | 94/140 | Non-blood disease controls | NO | 21 | | 73 | 28 | 111 | 9 |
| Aydin-Sayitoglu M [34] (2006) | ALL | Adults+Children | Caucasian | 155/140 | Non-blood disease controls | NO | 31 | | 124 | 29 | 111 | 9 |
| Bajpai P [35] (2007) | CML | Adults | Indian | 80/105 | Healthy controls | NO | 16 | | 64 | 9 | 96 | 11 |
| Pigullo S [36] (2007) | ALL | Children | Caucasian | 323/384 | Non-blood disease controls | NO | 44 | | 279 | 69 | 315 | 9 |
| Bolufer P [37] (2007) | AML | Adults+Children | Caucasian | 302/454 | Non-blood disease controls | NO | 74 | | 228 | 61 | 393 | 12 |
| Bolufer P [37] (2007) | ALL | Adults+Children | Caucasian | 141/454 | Non-blood disease controls | NO | 36 | | 105 | 61 | 393 | 12 |
| Eyada TK [39] (2007) | AML | unknown | Caucasian | 19/11 | Healthy controls | NO | 9 | | 10 | 5 | 6 | 7 |
| Eyada TK [39] (2007) | ALL | unknown | Caucasian | 13/11 | Healthy controls | NO | 3 | | 10 | 5 | 6 | 7 |
| Majumdar S [42] (2008) | AML | Adults+Children | Indian | 110/143 | Healthy controls | NO | 16 | | 94 | 11 | 132 | 11 |
| Muller P [43] (2008) | AML | Adults | Caucasian | 136/217 | Non-blood disease controls | NO | 25 | | 111 | 44 | 173 | 8 |
| Jiang LJ [44] (2008) | ALL | Adults | Asian | 88/120 | Healthy controls | Age and sex | 44 | | 44 | 59 | 61 | 12 |
| Chen HC [46] (2008) | ALL | Adults+Children | Asian | 120/204 | Healthy controls | NO | 61 | | 59 | 100 | 104 | 10 |
| Chen HC [46] (2008) | CML | Adults+Children | Asian | 108/204 | Healthy controls | NO | 51 | | 57 | 100 | 104 | 10 |
| Taspinar M [47] (2008) | CML | Adults | Caucasian | 107/130 | Healthy controls | NO | 43 | | 64 | 25 | 105 | 8 |
| Rimando MG [48] (2008) | ALL | Children | Asian | 60/60 | Healthy controls | NO | 21 | | 39 | 20 | 40 | 7 |
| Gra OA[49] (2008) | ALL | Children | Caucasian | 332/490 | Healthy controls | NO | 104 | | 228 | 94 | 396 | 10 |
| Gra OA[49] (2008) | AML | Children | Caucasian | 71/490 | Healthy controls | NO | 21 | | 50 | 94 | 252 | 10 |
| Souza CL[50] (2008) | AML | Adults+Children | Mixed | 23/304 | Non-blood disease controls | NO | 5 | | 18 | 25 | 279 | 10 |
| Souza CL[50] (2008) | CML | Adults+Children | Mixed | 53/304 | Non-blood disease controls | NO | 8 | | 45 | 25 | 279 | 10 |
| Jiang LJ [51] (2010) | ALL | Children | Asian | 89/90 | Healthy controls | NO | 54 | | 35 | 44 | 46 | 9 |
| Ovsepian [53] (2010) | CML | Adults | Caucasian | 83/205 | Healthy controls | NO | 20 | | 63 | 27 | 178 | 8 |
| Chan JY [55] (2011) | ALL | Children | Asian | 185/177 | Healthy controls | NO | 64 | | 121 | 49 | 128 | 10 |
| Suneetha KJ [56] (2011) | ALL | Adults+Children | Indian | 92/150 | Non-blood disease controls | NO | 11 | | 81 | 20 | 130 | 10 |
| Ouerhani S [57] (2011) | leukemia | Adults+Children | Caucasian | 193/309 | Healthy controls | NO | 66 | | 127 | 88 | 221 | 13 |
| Mandegary [59] (2011) | AML | Adults | Caucasian | 114/99 | Healthy controls | NO | 45 | | 69 | 23 | 76 | 10 |
| Chauhan PS [60] (2011) | AML | Adults | Indian | 120/202 | Healthy controls | Age and sex | 26 | | 94 | 58 | 144 | 12 |
| Chauhan PS [61] (2012) | AML | Adults+Children | Indian | 131/199 | Healthy controls | Age and sex | 27 | | 104 | 47 | 152 | 12 |
| Chauhan PS [61] (2012) | ALL | Adults+Children | Indian | 99/199 | Healthy controls | Age and sex | 28 | | 71 | 47 | 152 | 12 |
| Kim HN [62] (2012) | AML | Adults | Asian | 415/1700 | Non-blood disease controls | NO | 214 | | 201 | 859 | 841 | 10 |
| Li YH [63] (2012) | ALL | Children | Asian | 41/100 | Non-blood disease controls | NO | 24 | | 17 | 44 | 56 | 8 |
| Bhat G [64] (2012) | CML | Adults | Indian | 75/124 | Non-blood disease controls | Age and gender | 27 | | 48 | 26 | 98 | 12 |
| Lordelo [65] (2012) | CML | Adults | Mixed | 105/273 | Healthy controls | NO | 21 | | 84 | 65 | 208 | 9 |
| Ozten N [66] (2012) | CML | Adults | Caucasian | 106/190 | Healthy controls | Age and gender | 47 | | 59 | 35 | 155 | 11 |
| Dunna NR [69] (2013) | AML | Adults+Children | Indian | 142/251 | Non-blood disease controls | Age and sex | 57 | | 85 | 39 | 212 | 9 |
| Dunna NR [69] (2013) | ALL | Adults+Children | Indian | 152/251 | Non-blood disease controls | Age and sex | 38 | | 114 | 39 | 212 | 9 |
| Hou W [70] (2013) | ALL | Children | Asian | 100/112 | Non-blood disease controls | NO | 59 | | 41 | 55 | 57 | 9 |
| Zhou L [71] (2013) | AML | unknown | Asian | 163/204 | Non-blood disease controls | Age | 61 | | 102 | 55 | 149 | 10 |
| Moulik NR [72] (2014) | ALL | Children | Indian | 100/300 | Healthy controls | NO | 31 | | 69 | 45 | 255 | 10 |
| Al-Achkar [73] (2014) | CML | Adults | Caucasian | 126/172 | Non-blood disease controls | NO | 34 | | 92 | 27 | 145 | 10 |
| Zi Y [74] (2014) | AML | unknown | Asian | 206/231 | Non-blood disease controls | NO | 73 | | 133 | 55 | 176 | 9 |
| Guven [75] (2015) | ALL | Children | Caucasian | 95/190 | Healthy controls | NO | 24 | | 71 | 52 | 138 | 9 |
| Kassogue Y [76] (2015) | CML | Adults | Caucasian | 92/93 | Healthy controls | NO | 16 | | 76 | 9 | 84 | 10 |
| Nasr AS [77] (2015) | AML | Adults | Caucasian | 50/50 | Healthy controls | Age and sex | 30 | | 20 | 4 | 46 | 10 |
| Liu P [78] (2015) | leukemia | Adults | Asian | 442/442 | Non-blood disease controls | gender | 154 | | 270 | 114 | 306 | 12 |
| Bsnescu C [79] (2016) | AML | Adults | Caucasian | 102/303 | Healthy controls | NO | 24 | | 78 | 63 | 240 | 11 |
| Weich [80] (2016) | CML | Adults | Caucasian | 141/141 | Healthy controls | Age and sex | 20 | | 121 | 24 | 117 | 11 |
| Zehra A [82] (2018) | ALL | Adults | Caucasian | 62/62 | Healthy controls | Age and gender | 7 | | 55 | 4 | 58 | 10 |
| Brisson GD [83] (2018) | AML | Children | Mixed | 95/397 | Healthy controls | NO | 24 | | 71 | 90 | 307 | 12 |
| Farasani A [84] (2019) | AML | Adults | Caucasian | 100/100 | Healthy controls | NO | 13 | | 87 | 21 | 79 | 9 |
| Muddathir ARM [85] (2019) | CML | Adults | African | 115/104 | Healthy controls | NO | 76 | | 39 | 39 | 65 | 10 |
| Rostami G [86] (2019) | CML | Adults | Caucasian | 104/104 | Healthy controls | Age and sex | 3 | | 101 | 1 | 103 | 10 |
| Baba SM [87] (2020) | CML | Adults+Children | Indian | 150/150 | Healthy controls | NO | 25 | | 125 | 24 | 126 | 10 |
| Idris HM [88] (2020) | CML | unknown | African | 200/100 | Healthy controls | Age | 85 | | 115 | 21 | 79 | 10 |
| Baba SM [89] (2021) | ALL | Adults+Children | Indian | 150/150 | Healthy controls | Age and gender | 60 | | 90 | 24 | 126 | 11 |
| Abdalhabib EK [90] (2021) | CML | Adults | African | 150/150 | Healthy controls | Age and gender | 64 | | 86 | 27 | 123 | 12 |
| Abdalhabib EK [91] (2022) | ALL | Adults | African | 128/128 | Healthy controls | Age | 29 | | 99 | 19 | 109 | 13 |
