# Supplementary material for: Individual and combined effects of the GSTM1, GSTT1, and GSTP1 polymorphisms on leukemia risk: An updated meta-analysis
Source: Front Genet. 2022 Oct 31;13:976673. doi: 10.3389/fgene.2022.976673 (PMC9659912; doi:10.3389/fgene.2022.976673)
Supplement: Supplementary file 5 [file Table4.DOCX]

**Supplemental Table 3** Main characteristics and Quality score of studies included.

| **First Author/Year** | **Type of leukemia** | **Age group** | **Ethnicity** | **Sample size** | **Type of controls** | **Matching** | **GSTP1** | | | | | | **HWE** | **score** |
| --- | --- | --- | --- | --- | --- | --- | --- | --- | --- | --- | --- | --- | --- | --- |
|  |  |  |  |  |  |  | **cases** | | | **controls** | | |  |  |
|  |  |  |  |  |  |  | **lle/lle** | **Val/Val** | **lle/Val** | **lle/lle** | **Val/Val** | **lle/Val** |  |  |
| Allan JM [13] (2001) | AML | Adults | Caucasian | 420/1022 | Non-blood disease controls | Age and sex | 202 | 151 | 61 | 497 | 378 | 140 | 0 | 12 |
| Yuille [18] (2002) | CLL | Adults | Caucasian | 138/280 | Non-blood disease controls | NO | 59 | 63 | 16 | 140 | 105 | 28 | 0.2147 | 12 |
| Yuan XJ [21] (2003) | AL | Children | Asian | 120/85 | Healthy controls | NO | 63 | 52 | 5 | 58 | 25 | 2 | 0.7166 | 10 |
| Canalle R [24] (2004) | ALL | Children | Mixed | 113/221 | Non-blood disease controls | NO | 50 | 53 | 10 | 100 | 103 | 18 | 0.2289 | 11 |
| Pigullo S [36] (2007) | ALL | Children | Caucasian | 323/384 | Non-blood disease controls | NO | 162 | 130 | 31 | 203 | 148 | 33 | 0.4188 | 11 |
| Voso MT [38] (2007) | AML | Adults | Caucasian | 159/159 | Non-blood disease controls | Age and sex | 80 | 59 | 16 | 92 | 54 | 11 | 0.4329 | 13 |
| Gatedee J [40] (2007) | ALL | Children | Asian | 100/100 | Healthy controls | NO | 59 | 36 | 5 | 61 | 37 | 2 | 0.1766 | 10 |
| Suneetha KJ [45] (2008) | ALL | Adults+Children | Indian | 92/150 | Non-blood disease controls | NO | 43 | 40 | 9 | 81 | 57 | 12 | 0.6591 | 10 |
| Sailaja K [52] (2010) | CML | Adults | Indian | 260/248 | Healthy controls | Age and sex | 141 | 102 | 17 | 140 | 105 | 3 | 0.0006 | 12 |
| Chan JY [55] (2011) | ALL | Children | Asian | 185/177 | Healthy controls | NO | 97 | 50 | 13 | 99 | 61 | 10 | 0.8819 | 12 |
| Xi YM [58] (2011) | AML | Adults+Children | Asian | 102/150 | Non-blood disease controls | NO | 46 | 46 | 10 | 126 | 18 | 6 | 0 | 9 |
| Xi YM [58] (2011) | ALL | Adults+Children | Asian | 48/150 | Non-blood disease controls | NO | 38 | 6 | 4 | 126 | 18 | 6 | 0 | 8 |
| Mandegary A [59] (2011) | AML | Adults | Asian | 114/99 | Healthy controls | NO | 56 | 41 | 13 | 54 | 37 | 14 | 0.072 | 12 |
| Chauhan PS [60] (2011) | AML | Adults | Indian | 120/202 | Healthy controls | Age and sex | 59 | 52 | 9 | 103 | 84 | 15 | 0.7065 | 14 |
| Chauhan PS [61] (2012) | AML | Adults+Children | Indian | 131/199 | Healthy controls | Age and sex | 66 | 55 | 10 | 103 | 79 | 17 | 0.7382 | 14 |
| Chauhan PS [61] (2012) | ALL | Adults+Children | Indian | 99/199 | Healthy controls | Age and sex | 45 | 45 | 9 | 103 | 79 | 17 | 0.7382 | 14 |
| Kim HN [62] (2012) | AML | Adults | Asian | 415/1700 | Non-blood disease controls | NO | 274 | 118 | 14 | 1077 | 558 | 61 | 0.278 | 12 |
| Karkucak M [67] (2012) | CML | Adults | Caucasian | 71/67 | Non-blood disease controls | Age | 45 | 21 | 5 | 36 | 21 | 10 | 0.032 | 8 |
| Dunna NR [68] (2012) | AML | Adults+Children | Indian | 143/248 | Healthy controls | NO | 52 | 64 | 27 | 140 | 105 | 3 | 0.0006 | 11 |
| Dunna NR [68] (2012) | ALL | Adults+Children | Indian | 147/248 | Healthy controls | NO | 56 | 75 | 16 | 140 | 105 | 3 | 0.0006 | 11 |
| Zhou L [71] (2013) | AML | unknown | Asian | 163/204 | Non-blood disease controls | Age | 73 | 59 | 31 | 94 | 74 | 35 | 0.0037 | 10 |
| Moulik NR [72] (2014) | ALL | Children | Indian | 100/300 | Healthy controls | NO | 57 | 28 | 15 | 195 | 89 | 16 | 0.1731 | 12 |
| Zi Y [74] (2014) | AML | unknown | Asian | 206/231 | Non-blood disease controls | NO | 90 | 71 | 45 | 104 | 82 | 45 | 0.0003 | 9 |
| Guven M [75] (2015) | ALL | Children | Caucasian | 95/190 | Healthy controls | NO | 48 | 40 | 7 | 81 | 95 | 14 | 0.0503 | 11 |
| Nasr AS [77] (2015) | AML | Adults | Caucasian | 50/50 | Healthy controls | Age and sex | 20 | 18 | 12 | 32 | 8 | 10 | 0 | 10 |
| Liu P [78] (2015) | leukemia | Adults | Asian | 442/442 | Non-blood disease controls | gender | 288 | 122 | 12 | 302 | 109 | 9 | 0.8194 | 14 |
| Bsnescu C [79] (2016) | AML | Adults | Caucasian | 102/303 | Healthy controls | NO | 39 | 57 | 6 | 205 | 88 | 10 | 0.8827 | 13 |
| Weich [80] (2016) | CML | Adults | Caucasian | 141/141 | Healthy controls | Age and sex | 62 | 58 | 21 | 67 | 62 | 12 | 0.6582 | 13 |
| Al-Eitan LN [81] (2016) | ALL | Children | Caucasian | 88/176 | Healthy controls | NO | 45 | 34 | 9 | 130 | 41 | 5 | 0.4271 | 11 |
| Farasani A [84] (2019) | AML | Adults | Caucasian | 100/100 | Healthy controls | NO | 53 | 37 | 10 | 46 | 49 | 5 | 0.075 | 11 |
| Rostami G [86] (2019) | CML | Adults | Caucasian | 104/104 | Healthy controls | Age and sex | 54 | 38 | 12 | 66 | 33 | 5 | 0.7399 | 12 |
| Baba SM [87] (2020) | CML | Adults+Children | Indian | 150/150 | Healthy controls | NO | 57 | 68 | 25 | 92 | 51 | 7 | 0.984 | 12 |
| Idris HM [88] (2020) | CML | unknown | African | 200/100 | Healthy controls | Age | 130 | 63 | 7 | 62 | 36 | 2 | 0.2113 | 12 |
| Baba SM [89] (2021) | ALL | Adults+Children | Indian | 150/150 | Healthy controls | Age and gender | 88 | 47 | 15 | 92 | 51 | 7 | 0.984 | 13 |
